# A Framework for Improving the Reliability of Black-box Variational Inference

**Published:** 2024

**Authors:** Manushi Welandawe, Michael Riis Andersen, Aki Vehtari, Jonathan H. Huggins

**Affiliations:** Department of Mathematics & Statistics, Boston University, USA; DTU Compute, Technical University of Denmark, Denmark; Department of Computer Science, Aalto University, Finland; Department of Mathematics & Statistics, Faculty of Computing & Data Sciences, Boston University, USA

**Keywords:** black-box variational inference, symmetrized KL divergence, stochastic optimization, fixed-learning rate

## Abstract

Black-box variational inference (BBVI) now sees widespread use in machine learning and statistics as a fast yet flexible alternative to Markov chain Monte Carlo methods for approximate Bayesian inference. However, stochastic optimization methods for BBVI remain unreliable and require substantial expertise and hand-tuning to apply effectively. In this paper, we propose *robust and automated black-box VI* (RABVI), a framework for improving the reliability of BBVI optimization. RABVI is based on rigorously justified automation techniques, includes just a small number of intuitive tuning parameters, and detects inaccurate estimates of the optimal variational approximation. RABVI adaptively decreases the learning rate by detecting convergence of the fixed–learning-rate iterates, then estimates the symmetrized Kullback–Leibler (KL) divergence between the current variational approximation and the optimal one. It also employs a novel optimization termination criterion that enables the user to balance desired accuracy against computational cost by comparing (i) the predicted relative decrease in the symmetrized KL divergence if a smaller learning were used and (ii) the predicted computation required to converge with the smaller learning rate. We validate the robustness and accuracy of RABVI through carefully designed simulation studies and on a diverse set of real-world model and data examples.

## 1. Introduction

A core strength of the Bayesian approach is that it is conceptually straightforward to carry out inference in *arbitrary* models, which enables the user to employ whatever model is most appropriate for the problem at hand. The flexibility and uncertainty quantification provided by Bayesian inference have led to its widespread use in statistics (Robert, 2007; Gelman et al., 2013) and machine learning (Bishop, 2006; Murphy, 2012), including in deep learning (for example, Kingma and Welling, 2014; Rezende et al., 2014; Gal and Ghahramani, 2016; Maddox et al., 2019; Saatchi and Wilson, 2017; Johnson et al., 2016; Burda et al., 2016). Using Bayesian methods in practice, however, typically requires using approximate inference algorithms to estimate quantities of interest such as posterior functionals (for example, means, covariances, predictive distributions, and tail probabilities) and measures of model fit (for example, marginal likelihoods and cross-validated predictive accuracy). Therefore, a core challenge in modern Bayesian statistics is the development of *general-purpose* (or *black-box*) algorithms that can accurately approximate these quantities for whatever model the user chooses. In machine learning, rather than using Markov chain Monte Carlo (MCMC), *black-box variational inference* (BBVI) has become the method of choice because of its scalability and wide-applicability (Wainwright and Jordan, 2008; Blei et al., 2017; Kingma and Welling, 2014; Rezende et al., 2014; Burda et al., 2016). BBVI is implemented in many software packages for general-purpose inference such as Stan, Pyro, PyMC3, and TensorFlow Probability, which have seen widespread adoption by applied data analysts, statisticians, and data scientists.

Variational inference methods aim to minimize a measure of discrepancy between a parameterized family of distributions and the posterior distribution, with the Kullback–Leibler divergence being the canonical choice of discrepancy. Conventional approaches to variational inference leverage conditional conjugacy and other model-specific structure to derive optimization algorithms. BBVI, on the other hand, uses stochastic optimization to avoid the need for model-specific derivations, thereby significantly broadening the applicability of variational methods. Ensuring the efficiency and reliability of the BBVI optimization requires careful selection of optimization method and the stochastic estimator of the discrepancy gradient. For example, using adaptive optimization procedures like Adam, RMSProp, and Adagrad can ensure stability (Hinton and Tieleman, 2012; Kingma and Ba, 2015; Duchi et al., 2011). Due to its relatively small variance, the most common gradient estimator is the reparameterization gradient (Salimans and Knowles, 2013; Kingma and Welling, 2014; Rezende et al., 2014; Ruiz et al., 2016; Bamler et al., 2017; Domke, 2019). However, sometimes alternatives such as the score function gradient are employed (Paisley et al., 2012; Ranganath et al., 2014). No matter the choice of gradient estimator, various variance reduction strategies like control variates have been introduced to stabilize and speed up optimization (Miller et al., 2017; Roeder et al., 2017; Geffner and Domke, 2018, 2020; Boustati et al., 2020; Wang et al., 2023a,b). Recent research has furthered our understanding of convergence behavior, tackling theoretical challenges in stochastic optimization and providing new convergence guarantees (Domke et al., 2023; Kim et al., 2023).

While some recent progress has been made in developing tools for assessing the accuracy of variational approximations (Yao et al., 2018; Huggins et al., 2020; Wang et al., 2023c), stochastic optimization methods for BBVI remain unreliable and require substantial hand-tuning of the number of iterations and optimizer tuning parameters. Moreover, there are few tools available for determining whether the variational parameters estimated by these frameworks are close to optimal in any meaningful sense and, if not, how to address the problem; More iterations? A different learning rate schedule? A smaller initial or final learning rate? Agrawal et al. (2020) demonstrate the absence of a reliable and coherent optimization methodology for BBVI. The authors synthesize and compare recent advances such as normalizing flows and gradient estimators using 30 benchmarked models. Despite the fact that these models vary greatly in the complexity and dimensionality of the posteriors, Agrawal et al. (2020) run each optimization for a fixed number of iterations (30,000) and for 5 different step sizes because the existing literature does not provide any compelling guidance for how to automate the choice of step size and reliably determine when the optimization has converged.

### 1.1 Contributions

In view of the significant limitations of existing BBVI optimization methodology, in this paper we aim to provide a practical, cohesive, and theoretically well-grounded optimization framework for BBVI. To ensure reliability and wide applicability, we develop a framework that is (1) automated, (2) intuitively adjustable by the user, and (3) robust to failure and tuning parameter selection. Our approach builds on a recent line of work inspired by Pflug (1990), which uses a fixed learning rate *γ* that is adaptively decreased by a multiplicative factor *ρ* once the optimization iterates, which form a homogenous Markov chain, have converged (Chee and Toulis, 2018; Yaida, 2019; Pesme et al., 2020; Chee and Li, 2020; Zhang et al., 2020; Dhaka et al., 2020). A benefit of this approach is that, for a given learning rate, a dramatically more accurate estimate of the optimal variational parameter can be obtained by using *iterate averaging* (Ruppert, 1988; Polyak and Juditsky, 1992; Dieuleveut et al., 2020). However, as we have shown in previous work, existing convergence checks can be unreliable and stop too early (Dhaka et al., 2020). Since the learning rate is decreased by a constant multiplicative factor, decreasing it too early can slow down the optimization by an order of magnitude or more. Hence, it is crucial to develop methods that do not prematurely declare convergence. On the other hand, an optimization framework must also provide a termination criterion that triggers when it is no longer worthwhile to decrease the learning rate further, either because the current variational approximation is sufficiently accurate or because further optimization would be too time-consuming.

**Figure 1:**
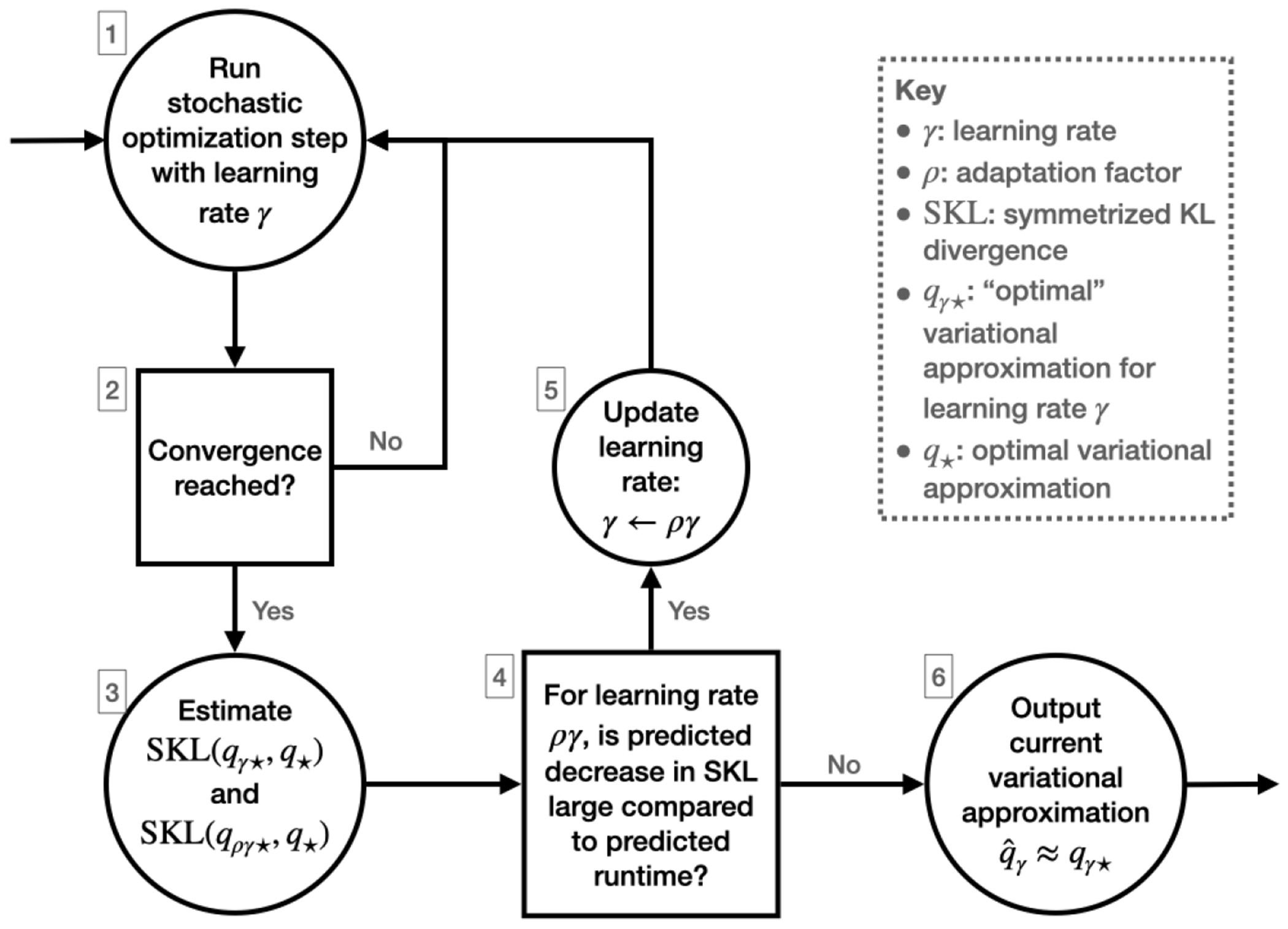
Schematic of the high-level logic of our proposed *robust and automated BBVI* (RABVI) framework.

The key idea that informs our solutions to these challenges is that we want $q_{\gamma^{*}}$, the target variational approximation for learning rate γ, to be close to the optimal variational approximation $q_{*}$. We measure closeness in terms of *symmetrized Kullback–Leibler (KL) divergence* $\text{SKL}\left(q_{*},q_{\gamma_{*}}\right)$ and show that closeness in symmetrized KL divergence can be translated into bounds on other widely used accuracy metrics like Wasserstein distance (Huggins et al., 2020; Bolley and Villani, 2005). Figure 1 summarizes our proposed framework, which we call *robust and automated black-box VI (RABVI)*. The primary contributions of this paper are in steps 2, 3, and 4. In step 2, to determine convergence at a fixed step size, we build upon our approach in Dhaka et al. (2020), where we establish that the scale-reduction factor $\hat{R}$ (Gelman and Rubin, 1992; Gelman et al., 2013; Vehtari et al., 2021), which is widely used to determine convergence of Markov chain Monte Carlo algorithms, can be combined with a Monte Carlo standard error (MCSE) (Geyer, 1992; Vehtari et al., 2021) cutoff to construct a convergence criteria. We improve upon our previous proposal by:

- adaptively finding the size of the convergence window, which may need to be large for challenging or high-dimensional distributions over the model parameters, and
- developing a new rigorous MCSE cutoff condition that guarantees the symmetrized KL divergence between $q_{\rho \gamma *}$ and the estimate of $q_{\rho \gamma *}$ obtained via iterate averaging will be small.

**Figure 2:**
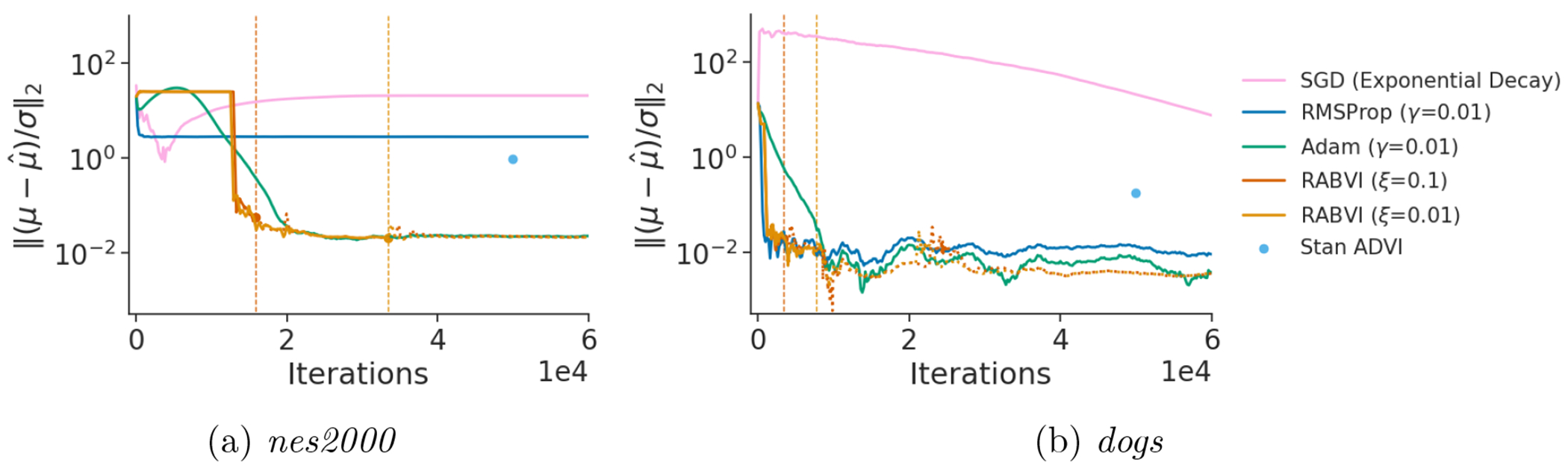
Accuracy comparison of variational inference algorithms using two data sets/models from the posteriordb package (see Section 7.2 for details). Accuracy is measured in terms of relative mean error $\|(\mu -\overset{ˆ}{\mu })/\sigma {\|}_{2}$, where *μ* and *σ* are, respectively, the posterior mean and standard deviation vectors and $\overset{ˆ}{\mu }$ is the variational approximation to *μ*. The vertical lines indicate the termination points for RABVI, which uses averaged Adam (see Section 4.3). The fixed-learning rate algorithms have a learning rate *γ* and RABVI has a user-specified accuracy threshold ξ. For SGD exponential decay, we use an initial learning rate of 0.01 for *dogs* but a smaller initial learning rate of 0.001 for *nes2000* due to optimization instability.

In step 3, we leverage recent results that characterize the bias of the stationary distribution of stochastic gradient descent (SGD) with a fixed learning rate (Dieuleveut et al., 2020) to estimate $\text{SKL}\left(q_{*},q_{\gamma *}\right)$ and $\text{SKL}\left(q_{*},q_{\rho \gamma *}\right)$ without access to $q_{*}$. In step 4, these estimates enable the use of a termination criterion that compares (i) the predicted relative decrease in the KL divergence if the smaller learning ργ were used and (ii) the predicted computation required to converge with the learning rate ργ. By trading off between (i) and (ii), the criterion enables the user to balance desired accuracy against computational cost. Figure 2 provides an example of the faster convergence, higher accuracy, and greater reliability achievable using RABVI compared to alternative optimization algorithms and demonstrates how the user can trade off accuracy and computation by adjusting the accuracy threshold ξ.

In summary, by drawing on recent developments in theory and methods for fixed–learning-rate stochastic optimization, tools from MCMC methodology and results from functional analysis, RABVI delivers a number of benefits:

- it relies on rigorously justified automation techniques, including automatic learning rate adaptation;
- it has an interpretable, user-adjustable accuracy parameter along with a small number of additional intuitive tuning parameters;
- it detects inaccurate estimates of the optimal variational approximation; and
- it can flexibly incorporate additional or future methodological improvements related to variational inference and stochastic optimization.

We demonstrate through synthetic comparisons and real-world model and data examples that RABVI provides high-quality black-box approximate inferences. We make RABVI available as part of the open source Python package VIABEL.^1^

## 2. Preliminaries and Background

In this section, we briefly review relevant background about Bayesian and variational inference.

### 2.1 Bayesian Inference

Let $\theta \in R^{d}$ denote a parameter vector of interest, and let *x* denote observed data. A Bayesian model consists of a prior density $\pi_{0}(d\theta )$ and a likelihood ℓ(x;θ). Together, the prior and likelihood define a joint distribution over the data and parameters. The Bayesian posterior distribution *π* is the conditional distribution of θ given fixed data *x*, with *x* suppressed in the notation since it is always fixed throughout this work. To write this conditional, we define the unnormalized posterior density $\pi^{u}(\theta ):=\ell (x;\theta )\pi_{0}(d\theta )$ and the marginal likelihood, or evidence, $Z:=\int \pi^{u}(d\theta )$. Then the posterior is $\pi :=\pi^{u}/Z$. Typically, practitioners report posterior summaries, such as point estimates and uncertainties, rather than the full posterior. For ϑ∼π, typical summaries include the mean $m_{\pi }:=E(\vartheta )$, the covariance $\Sigma_{\pi }:=E\{\left(\vartheta -m_{\pi }\right){\left(\vartheta -m_{\pi }\right)}^{⊤}\}$, and [*a, b*] interval probability $I_{\pi ,i,a,b}:=P\left(\vartheta_{i}\in [a,b]\right)=E\{𝟙\left(\vartheta_{i}\in [a,b]\right)\}$, where 𝟙(C) is equal to one when *C* is true and zero otherwise.

### 2.2 Variational Inference

In most Bayesian models, it is infeasible to efficiently compute quantities of interest such as posterior means, variances, and quantiles. Therefore, one must use an approximate inference method that produces an approximation *q* to the posterior π. The summaries of *q* may in turn be used as approximations to the summaries of π. One approach, *variational inference*, is widely used in machine learning. Variational inference aims to minimize some measure of discrepancy ${𝒟}_{\pi }(\cdot )$ over a tractable family $𝒬=\left\{q_{\lambda }:\lambda \in R^{m}\right\}$ of approximating distributions (Wainwright and Jordan, 2008; Blei et al., 2017):

$$q_{\lambda_{\text{*}}}=\underset{q_{\lambda }\in 𝒬}{\text{arg min}}{𝒟}_{\pi }\left(q_{\lambda }\right).$$

The variational family 𝒬 is chosen to be tractable in the sense that, for any q∈𝒬, we are able to efficiently calculate relevant summaries either analytically or using independent and identically distributed samples from *q*.

In variational inference, the standard choice for the discrepancy ${𝒟}_{\pi }(\cdot )$ is the *Kullback–Leibler (KL) divergence* KL(q∣π):=∫log(dq/dπ)dq (Bishop, 2006). Note that the KL divergence is asymmetric in its arguments. The direction ${𝒟}_{\pi }(q)=KL(q|\pi )$ is most typical in variational inference, largely out of convenience; the unknown marginal likelihood *Z* appears in an additive constant that does not influence the optimization and computing gradients require estimating expectations only with respect to q∈𝒬, which is chosen to be tractable. Minimizing KL(q∣π) is equivalent to maximizing the *evidence lower bound* (ELBO; Bishop, 2006):

$$\text{ELBO}(q):=\int \text{log}\left(\frac{\text{d}\pi^{u}}{\text{d}q}\right)\text{d}q.$$

While numerous other divergences have been used in the literature (for example, Hernáandez-Lobato et al., 2016; Li and Turner, 2016; Bui et al., 2017; Dieng et al., 2017; Wang et al., 2018; Wan et al., 2020), we focus on KL(q∣π) since it is the most common choice; the default or only choice in widely used frameworks such as Stan, Pyro, and PyMC3; and easiest to estimate when using simple Monte Carlo sampling to approximate the gradient (Dhaka et al., 2021).

### 2.3 Black-box Variational Inference

Black-box variational inference (BBVI) methods have greatly extended the applicability of variational inference by removing the need for model-specific derivations (Cornebise et al., 2008; Ranganath et al., 2014; Kucukelbir et al., 2015; Titsias and Lázaro-Gredilla, 2014; Mohamed et al., 2020) and enabling the use of more flexible approximation families (Kingma and Welling, 2014; Salimans et al., 2015; Papamakarios et al., 2021). This flexibility is a result of using simple Monte Carlo (and automatic differentiation) to approximate the (gradients of the) expectations that define common choices of the discrepancy objective (Papamakarios et al., 2021; Mohamed et al., 2020). To estimate the optimal variational parameter λ_*_, BBVI methods commonly use stochastic optimization schemes which at iteration *k* are of the form

(1) $${\text{λ}}^{\left(k+1\right)}\leftarrow {\text{λ}}^{\left(k\right)}-{\text{γ}}^{\left(k\right)}d^{\left(k\right)},$$

where $d^{\left(k\right)}\in R^{m}$ is the *descent direction* and $\gamma^{\left(k\right)}>0$ is the *learning rate* (also called the *step size*). Standard stochastic gradient descent corresponds to taking $d^{\left(k\right)}={\overset{ˆ}{g}}^{\left(k\right)}$, a (usually unbiased) stochastic estimate of the gradient $g(\lambda^{\left(k\right)}):=\nabla_{\lambda^{\left(k\right)}}D_{\pi }\left(q_{\lambda }(k)\right)$. We are particularly interested in adaptive stochastic optimization methods (for example, Duchi et al., 2011; Hinton and Tieleman, 2012; Kingma and Ba, 2015) that use a smoothed and/or rescaled version of ${\overset{ˆ}{g}}^{\left(k\right)}$ as the descent direction. For example, RMSProp (Hinton and Tieleman, 2012) tracks an exponential moving average of the squared gradient, $\nu^{\left(k+1\right)}=\beta \nu^{\left(k\right)}+(1-\beta ){\overset{ˆ}{g}}^{\left(k\right)}⊙{\overset{ˆ}{g}}^{\left(k\right)}$, which is used to rescale the current stochastic gradient: $d^{\left(k\right)}={\overset{ˆ}{g}}^{\left(k+1\right)}/\sqrt{\nu^{\left(k\right)}}$. Or, Adam (Kingma and Ba, 2015) tracks an exponential moving average of the gradient $m^{\left(k+1\right)}=\alpha m^{\left(k\right)}+(1-\alpha ){\overset{ˆ}{g}}^{\left(k\right)}$ as well as the squared gradient $\nu^{\left(k+1\right)}$ and uses both to rescale the current stochastic gradient: $d^{\left(k\right)}=m^{\left(k\right)}/\sqrt{\nu^{\left(k\right)}}$. The benefits of adaptive algorithms include that they tend to be more stable and are scale invariant, so the learning rate can be set in a problem-independent manner.

### 2.4 Fixed–Learning-Rate Stochastic Optimization

If the learning rate is fixed so that $\gamma^{\left(k\right)}=\gamma$, then we can view the iterates $\lambda^{\left(1\right)},\lambda^{\left(2\right)},\ldots$ produced by Eq. (1) as a homogenous Markov chain, which under certain conditions will have a stationary distribution $\mu_{\gamma }$ (Dieuleveut et al., 2020; Gitman et al., 2019; Pflug, 1990; Chee and Toulis, 2018; Yaida, 2019). Stochastic optimization with a fixed learning rate exhibits two distinct phases: a transient (a.k.a. warm-up) phase during which iterates make rapid progress toward the optimum, followed by a stationary (a.k.a. mixing) phase during which iterates oscillate around the mean of the stationary distribution, ${\overset{‾}{\lambda }}_{\gamma }:=\int \lambda \mu_{\gamma }(d\lambda )$ (Gelman and Rubin, 1992).

The mean ${\overset{‾}{\lambda }}_{\gamma }$ is a natural target because even if the variance of the individual iterates $\lambda^{\left(k\right)}$ means they are far from $\lambda_{*}$, ${\overset{‾}{\lambda }}_{\gamma }$ can be a much more accurate approximation to $\lambda_{*}$. For example, the following result quantifies the bias of standard fixed–learning-rate SGD (Gitman et al. (2019) provide similar results for momentum-based SGD algorithms):

**Theorem 1** (Dieuleveut et al. (2020, **Theorem 4))** *Under regularity conditions on the objective function and the unbiased stochastic gradients, there exist constant vectors* $A,B\in R^{m}$ *such that*^2^

(2) $${\bar{\lambda }}_{\gamma }{-\lambda }_{\text{*}}=A\gamma +B\gamma^{2}+o\left(\gamma^{2}\right)$$

*and a matrix* $A^{\prime }\in R^{m\times m}$ *such that*

$$\int \left({\lambda -\lambda }_{\text{*}}\right){\left({\lambda -\lambda }_{\text{*}}\right)}^{⊤}\mu_{\gamma }(\text{d}\lambda )=A^{'}\gamma +O\left(\gamma^{2}\right).$$

**Remark 2** *The regularity conditions required by Theorem 1 are mostly mild. For example, the stochastic gradients must be unbiased and have finite variance that does not grow too quickly away from the optimum. However, it does require the stronger assumptions that the objective function is smooth and strongly convex. While these conditions do not hold globally, we do not view it be a significant problem in practice because near the optimum we expect the objective function to be locally smooth and strongly convex*.

Theorem 1 shows that, at stationarity, a single iterate will satisfy $\lambda^{\left(k\right)}-\lambda_{*}=O\left(\gamma^{1/2}\right)$ (with high probability) while its expectation will satisfy ${\overset{‾}{\lambda }}_{\gamma }-\lambda_{*}=O(\gamma )$. Therefore, when the learning rate is small, ${\overset{‾}{\lambda }}_{\gamma }$ is a substantially better estimator for $\lambda_{*}$ than $\lambda^{\left(k\right)}$. In practice the *iterate average* (that is, the sample mean)

(3) $${\hat{\lambda }}_{\gamma }:=\frac{1}{k^{\text{avg}}}\overset{k^{\text{avg}}-1}{\underset{k=0}{\sum }}\lambda^{\left(k^{\text{conv}}+k\right)}$$

provides an estimate of ${\overset{‾}{\lambda }}_{\gamma }$, where $k^{\text{conv}}$ is the iteration at which the optimization has reached the stationary phase and $k^{\text{avg}}$ is the number of iterations used to compute the average. Using ${\overset{ˆ}{\lambda }}_{\gamma }$ as an estimate of $\lambda_{*}$ is known as Polyak–Ruppert averaging (Polyak and Juditsky, 1992; Ruppert, 1988; Bach and Moulines, 2013).

When using iterate averaging, it is crucial to ensure the iterate average accurately approximates ${\overset{‾}{\lambda }}_{\gamma }$. Considering a stationary Markov chain, we can compute the Monte Carlo estimate of the mean of the Markov Chain at stationarity. Then the notion of *effective sample size* (ESS) aids in quantifying the accuracy of this Monte Carlo estimate. Further, the effective sample size can also be used to define the *Monte Carlo standard error* (MCSE) when the Markov chain satisfies a central limit theorem. We can efficiently estimate the ESS and also approximate the MCSE (see Appendix B.1 for details). We denote the estimates of ESS and MCSE for the *i*th component using iterates $\lambda_{i}^{\left(k^{\text{conv}}:k\right)}$ as $\hat{ESS}(\lambda_{i}^{\left(k^{\text{conv}}:k\right)})$ and $\hat{MCSE}(\lambda_{i}^{\left(k^{\text{conv}}:k\right)})$ respectively. Dhaka et al. (2020) use the conditions $m^{-1}\sum_{i=1}^{m}\hat{MCSE}(\lambda_{i}^{\left(k^{\text{conv}}:k\right)})<0.02$ and $\hat{ESS}(\lambda_{i}^{\left(k^{\text{conv}}:k\right)})>20$ to determine when to stop iterate averaging. However, no rigorous justification is given for the MCSE threshold.

### 2.5 Automatically Scheduling Learning Rate Decreases

A benefit of using a fixed learning rate is that it can be adaptively and automatically decreased once the iterates reach stationarity. For example, if the initial learning rate is $\gamma_{0}$, after reaching stationarity the learning rate can decrease to $\gamma_{1}:=\rho \gamma_{0}$, where ρ∈(0,1) is a user-specified adaptation factor. The process can be repeated: when stationarity is reached at learning rate $\gamma_{t}$, the learning rate can decrease to $\gamma_{t+1}:=\rho \gamma_{t}$. In this way the learning rate is not decreased too early (when the iterates are still making fast progress toward the optimum) or too late (when the accuracy of the iterates is no longer improving). Compare this adaptive approach to the canonical one of setting a schedule such as $\gamma^{\left(k\right)}=\Delta /(◯+k{)}^{◻}$, which requires the choice of three tuning parameters. These tuning parameters can have a dramatic effect on the speed of convergence, particularly when $\lambda^{\left(0\right)}$ is far from $\lambda_{*}$.

The question of how to determine when the stationary phase has been reached has a long history with recent renewed attention (Pesme et al., 2020; Zhang et al., 2020; Chee and Li, 2020; Lang et al., 2019; Yaida, 2019; Pflug, 1990; Chee and Toulis, 2018; Dhaka et al., 2020). One line of work (Yaida, 2019; Lang et al., 2019; Zhang et al., 2020) is based on finding an invariant function that has expectation zero under the stationary distribution of the iterates, then using a test for whether the empirical mean of the invariant function is sufficiently close to zero. An alternative approach developed in Dhaka et al. (2020) makes use of the potential scale reduction factor $\hat{R}$, perhaps the most widely used MCMC diagnostic for detecting stationarity (Gelman and Rubin, 1992; Gelman et al., 2013; Vehtari et al., 2021). The standard approach to computing $\hat{R}$ is to use multiple Markov chains. If we have J≥2 chains and K≫1 iterates in each chain, then $\hat{R}:=(\overset{ˆ}{V}/\overset{ˆ}{W}{)}^{1/2}$, where $\overset{ˆ}{V}$ and $\overset{ˆ}{W}$ are estimates of, respectively, the between-chain and within-chain variances. In the split-$\hat{R}$ version, each chain is split into two before carrying out the computation above, which helps with detecting non-stationarity (Gelman et al., 2013; Vehtari et al., 2021) and allows for use even when *J* = 1. Let ${\hat{R}}_{i}(W)$ denote the split-$\hat{R}$ value computed from $\lambda_{i}^{\left(k-W+1\right)},\ldots ,\lambda_{i}^{\left(k\right)}$, the *i*th dimension of the last *W* iterates. Dhaka et al. (2020) uses the stationarity condition ${max}_{i}{\hat{R}}_{i}(100)<1.1$,

## 3. Methodological Criteria

We now summarize our criteria when designing a robust and automatic optimization framework for BBVI.

### Robustness.

A robust method should not be too sensitive to the choice of tuning parameters. It should also work well on a wide range of “typical” problems. To achieve this we design an adaptive methods for setting parameters (such as the window size for detecting convergence) that are problem-dependent.

### Automation.

An automatic method should require minimal input from the user. Any inputs that are required should be clearly necessary (for example, the model and the data) or be intuitive to an applied user who is not an expert in variational inference and optimization. Therefore, we require the parameters of any adaptation scheme to either be intuitive or not require adjustment by the user. Examples of intuitive parameters include the maximum number of iterations, maximum runtime, and, when defined appropriately, accuracy.

We ensure these criteria are satisfied when designing the two core components of a BBVI stochastic optimization framework with automated learning rate scheduling:

1. A **termination rule** for stopping the optimization once the final approximation is close to the optimal approximation (Section 4).
2. A **learning rate scheduler**, which must detect stationarity and determine how many iterates to average before decreasing the learning rate (Section 5).

## 4. Termination Rule

The development of our termination rule will proceed in three steps. First, we will select an appropriate discrepancy measure between distributions. Next, we will design an idealized termination rule based on this discrepancy measure. Finally, we will develop an implementable version of the idealized termination rule that satisfies the criteria from Section 3.

### 4.1 Choice of Accuracy Measure

To develop a termination rule, we must specify a measure of how close a variational approximation returned by the optimization algorithm, ${\overset{ˆ}{q}}_{*}$, is to the optimal variational approximation $q_{*}:=q_{\lambda_{*}}$. But the answer to this question depends upon choosing an appropriate measure of the discrepancy between $q_{*}$ and the posterior π. Based on the discussion in Section 2.1, the goal should be for quantities such as $m_{q_{*}}$, $\Sigma_{q_{*}}$, and $I_{q_{*},i,a,b}$ to be close to, respectively, $m_{\pi }$, $\Sigma_{\pi }$, and $I_{\pi ,i,a,b}$. The interval probabilities are already on an interpretable scale, so ensuring that $\left|I_{q,i,a,b}-I_{\pi ,i,a,b}\right|$ is much less than 1 is an intuitive notion of accuracy. Since $\Sigma_{\pi }^{1/2}$ establishes the relevant scale of the problem for means and standard deviations, so it is appropriate to ensure that ${\left\|\Sigma_{\pi }^{-1/2}\left(m_{\pi }-m_{q_{*}}\right)\right\|}_{2}$ and ${\left\|\Sigma_{\pi }^{-1}\left(\Sigma_{\pi }-\Sigma_{q_{*}}\right)\right\|}_{2}={\left\|I-\Sigma_{\pi }^{-1}\Sigma_{q_{*}}\right\|}_{2}$ are much less than 1.

While we want to choose a discrepancy measure that guarantees the accuracy of mean, covariance, and interval probabilities, ideally it would also guarantee other plausible expectations of interest (for example, predictive densities) are accurately approximated. The *Wasserstein distance* provides one convenient metric for accomplishing this goal, and is widely used in the analysis of MCMC algorithms and in large-scale data asymptotics (for example, Joulin and Ollivier, 2010; Madras and Sezer, 2010; Rudolf and Schweizer, 2018; Durmus et al., 2019; Durmus and Moulines, 2019; Vollmer et al., 2016; Eberle and Majka, 2019). For p≥1 and a positive-definite matrix $\Sigma \in R^{d\times d}$, we define the (*p*, Σ)-*Wasserstein distance* between distributions η and ζ as

$${𝒲}_{p,\Sigma }\left(\eta ,\zeta \right):=\underset{\omega }{\text{inf}}{\left\{\int {\left\|\Sigma^{-1/2}\left(\theta -\theta^{\prime }\right)\right\|}_{2}^{p}\omega \left(\text{d}\theta ,\text{d}\theta^{\prime }\right)\right\}}^{1/p},$$

where the infimum is over the set of *couplings* between η and ζ; that is, Borel measures ω on $R^{d}\times R^{d}$ such that $\eta =\omega \left(\cdot ,R^{d}\right)$ and $\zeta =\omega \left(R^{d},\cdot \right)$ (Villani, 2009, Defs. 6.1 & 1.1). Small (*p*, Σ)-Wasserstein distance implies many functionals of the two distributions are close relative to the scale determined by $\Sigma^{1/2}$.

Specifically, we have the following result, which is an immediate corollary of Huggins et al. (2020, Theorem 3.4).

**Proposition 3** *If* ${𝒲}_{p,\Sigma }(\eta ,\zeta )\leq \varepsilon$ *for any* p≥1, *then*

$${\left\|{\text{Σ}}^{-1/2}\left(m_{\eta }-m_{\zeta }\right)\right\|}_{2}\leq \varepsilon$$

*If* ${𝒲}_{2,\Sigma }(\eta ,\zeta )\leq \varepsilon$, *then, for* $\varrho :=min\left\{{\left\|\Sigma^{-1}\Sigma_{\eta }\right\|}_{2}^{1/2},{\left\|\Sigma^{-1}\Sigma_{\zeta }\right\|}_{2}^{1/2}\right\}$,

$${\left\|{\text{Σ}}^{-1}\left({\text{Σ}}_{\eta }-{\text{Σ}}_{\zeta }\right)\right\|}_{2}<2\varepsilon (\varrho +\varepsilon ).$$

More generally, small (*p*, Σ)-Wasserstein distance for any p≥1 guarantees the accuracy of expectations for any function *f* with small Lipschitz constant with respect the metric $d_{\Sigma }\left(\theta ,\theta^{\prime }\right):={\left\|\Sigma^{-1/2}\left(\theta -\theta^{\prime }\right)\right\|}_{2}$; that is, when ${sup}_{\theta \neq \theta^{\prime }}\left|f(\theta )-f\left(\theta^{\prime }\right)\right|/d_{\Sigma }\left(\theta ,\theta^{\prime }\right)$ is small.

While the Wasserstein distance controls the error in mean and covariance estimates, it does not provide strong control on interval probability estimates. The KL divergence, however, does, since for distributions η and ζ, $\left|I_{\eta ,i,a,b}-I_{\zeta ,i,a,b}\right|\leq \sqrt{KL(\eta |\zeta )/2}$ for all *a < b* and *i* (see Appendix B.2). As we show next, in many scenarios we can bound the Wasserstein distance by the KL divergence and therefore enjoy the benefits of both. Our result is based on the following definition, which makes the notion of the scale of a distribution precise:

**Definition 4** *For* p≥1 *and a positive-definite matrix* $\Sigma \in R^{d\times d}$, *the distribution* η *is said to be* (*p*, Σ)-exponentially controlled *if*

(4) $$\underset{\theta^{\prime }}{\text{inf}}\;\text{log}\int e^{{\left\|\Sigma^{-1/2}\left(\theta -\theta^{'}\right)\right\|}_{2}^{p}}\eta (\text{d}\theta )\leq d/2.$$

Specially, $\Sigma^{1/2}$ establishes the appropriate scale for uncertainty with respect to η. For example, if η=𝒩(m,V), then it is a straightforward exercise to confirm that η is (2, 1.78^2^*V*)-exponentially controlled.

The following result establishes the relevant link between the KL divergence and the Wasserstein distance via Definition 4.

**Proposition 5** *If η is* (*p*, Σ)-*exponentially controlled, then for all* ζ *absolutely continuous with respect to* η,

$${𝒲}_{p,\text{Σ}}(\zeta ,\eta )\leq (3+d){𝒦}_{p}(\zeta |\eta ),$$

*where* ${𝒦}_{p}(\zeta |\eta ):=KL(\zeta |\eta {)}^{\frac{1}{p}}+\{KL(\zeta |\eta )/2{\}}^{\frac{1}{2p}}$.

**Proof** The result follows from Bolley and Villani (2005, Corollary 2.3) after the change-of-variable $\theta \mapsto \Sigma^{-1/2}\theta$ and using the fact that the KL divergence is invariant under diffeomorphisms, then applying Eq. (4).∎

If ζ and η could operate over different scales, then we can use the *symmetrized KL divergence* SKL(ζ,η):=KL(ζ∣η)+KL(η∣ζ). Indeed, it follows from Proposition 5 that if SKL(ζ,η) is small, then the (*p*, Σ)-Wasserstein distance is small whenever *either* η or ζ is (*p*, Σ)-exponentially controlled.

### 4.2 An Idealized Termination Rule

Based on our developments in Section 4.1, we will define our termination rule in terms of the symmetrized KL divergence. Recall that $q_{*}$ denotes the optimal variational approximation to π and ${\overset{ˆ}{q}}_{*}$ denotes an estimate of $q_{*}$. Since the total variation and (1, Σ)-Wasserstein distances are controlled by the square root of the KL divergence, we focused on the square root of the symmetrized KL divergence. For the current learning rate γ>0, let $q_{\gamma *}:=q_{{\overset{‾}{\lambda }}_{\gamma }}$ denote the target γ–learning-rate variational approximation. The termination rule we propose is based on the trade-off between the improved accuracy of the approximation if the learning rate were reduced to ργ and the time required to reach that improved accuracy. To quantify the improved accuracy, we introduce a user-chosen target accuracy target ξ for $\text{SKL}{\left(q_{*},{\overset{ˆ}{q}}_{*}\right)}^{1/2}$. If the user expects $KL\left(\pi |q_{*}\right)$ to be large, then setting ξ to a moderate value such as 1 or 10 could give acceptable performance. If the user expects $KL\left(\pi |q_{*}\right)$ to be small, then setting ξ to a value such as 0.1 or 0.01 might be more appropriate. Using ξ, define the *relative SKL improvement*

$$\text{RSKL}:=\frac{\text{SKL}{\left(q_{*},q_{\rho \gamma *}\right)}^{1/2}+\xi }{\text{SKL}{\left(q_{*},q_{\gamma_{*}}\right)}^{1/2}},$$

where the first term measures the relative improvement of the approximation if the learning rate were reduced and the second term measures the current accuracy relative to the desired accuracy. To quantify the time to obtain the relative accuracy improvement, we use the number of iterations to reach convergence for the fixed learning rate ργ. Letting $K_{\gamma *}$ denote the number of iterations required to reach the target γ–learning-rate variational approximation, we define the *relative iteration increase*

$$\text{RI}:=\frac{K_{\rho \gamma^{*}}}{K_{\gamma^{*}}+K_{0}},$$

where $K_{0}$ denotes the number of iterations the user would consider “small”. Combining RSKL and RI, we obtain the *inefficiency index* ℐ=RSKL×RI, the relative improvement in accuracy times the relative increase in runtime. Thus, we can interpret ℐ as quantifying how much greater the increase in runtime cost (above a baseline of $K_{0}$ iterations) will be compared to the reduction in error (down to a target error of ξ). For example, ℐ=2 means the increase in runtime cost is twice as large as the reduction in error. Our *idealized SKL inefficiency termination rule* triggers when ℐ>τ, where τ is a user-specified inefficiency threshold that allows the user to trade off accuracy with computation, but only up to the point where $SKL{\left(q_{*},q_{\gamma *}\right)}^{1/2}\approx \xi$.

### 4.3 An Implementable Termination Rule

The idealized SKL inefficiency termination rule cannot be directly implemented since $q_{*}$ is unknown; and if it were known, it would be unnecessary to run the optimization algorithm. However, we will show that it is possible to obtain a good estimate of the symmetrized KL divergence between the approximation obtained with a given learning rate $\gamma^{\prime }$ and the optimal approximation without access to $q_{*}$.

Recall that $q_{\gamma *}$ denotes the target γ–learning-rate variational approximation. With a slight abuse of notation, we let the optimal zero–learning-rate approximation refer to the optimal approximation: $q_{0*}:={lim}_{\gamma \to 0}q_{\gamma *}=q_{*}$. Our approach is motivated by Theorem 1 and in particular the form of the bias ${\overset{‾}{\lambda }}_{\gamma }-\lambda_{*}$ in Eq. (2). We first consider the still-common setting when 𝒬 is the family of mean-field Gaussian distributions, where the parameter $\lambda =(\tau ,\psi )\in R^{2d}$ corresponds to the distribution $q_{\lambda }=𝒩\left(\tau ,diage^{2\psi }\right)$.

**Proposition 6** *Let* 𝒬 *be the family of mean-field Gaussian distributions. If* Eq. (2) *holds and* $\gamma^{\prime }=O(\gamma )$, *then there is a constant* C>0 *depending only on A and* $\lambda_{*}$ *such that*

$$\text{SKL}\left(q_{\gamma *},q_{\gamma^{\prime }\text{*}}\right)=C{\left(\gamma -\gamma^{\prime }\right)}^{2}+o\left(\gamma^{2}\right).$$

See Appendix A.1 for the proof. Assuming that the current learning rate is γ, then the previous learning rate was γ/ρ. Let $\delta_{\gamma }:=SKL(q_{\gamma *},q_{\gamma /\rho *})$ denote the symmetrized KL divergence between the optimal variational approximations obtained at each of these learning rates. In principle we can use Proposition 6 to estimate *C* by

$$\hat{C}=\delta_{\gamma }\rho^{2}/\left\{\gamma^{2}{\left(1-\rho \right)}^{2}\right\},$$

and then estimate that

$$\text{SKL}\left(q_{\gamma \text{*}},q_{\text{*}}\right)\approx \hat{C}\gamma^{2}=\delta_{\gamma }\rho^{2}/{\left(1-\rho \right)}^{2}.$$

There are, however, two problems with the tentative approach just outlined. The first problem is that Theorem 1 only holds for standard SGD; however, adaptive SGD algorithms are widely used in practice. Indeed, we observe empirically that $SKL\left(q_{\gamma *},q_{\gamma^{\prime }*}\right)=\Theta (|\gamma -{\left.\gamma^{\prime }\right|}^{\kappa /2}$) with κ≈1 for RMSProp (Fig. C.6) and κ∈(1,1.6) (with a point estimate at 1.2) for Adam Fig. C.7). Hence RMSProp and Adam both appear to have larger errors than SGD when the step size is small. To get the accuracy of SGD but also adaptivity, we modify the adaptive gradient methods to behave asymptotically (in the number of iterations) like SGD. In the cases of RMSProp and Adam, we propose *averaged RMSProp* (avgRMSProp) and *averaged Adam* (avgAdam), which use the squared gradient update

$$\nu^{\left(k+1\right)}=\beta_{k}\nu^{\left(k\right)}+\left(1-\beta_{k}\right){\hat{g}}^{\left(k\right)}⊙{\hat{g}}^{\left(k\right)},$$

for $\beta_{k}=1-1/k$. Hence, $\nu^{\left(k+1\right)}=(k+1{)}^{-1}\sum_{k^{\prime }=0}^{k}{\overset{ˆ}{g}}^{\left(k\right)}⊙{\overset{ˆ}{g}}^{\left(k\right)}$ is the averaged squared gradient over all iterations (Mukkamala and Hein, 2017, §4). As long as the SGD Markov chain is ergodic and $E\left[{\left\|{\overset{ˆ}{g}}^{\left(k\right)}\right\|}_{2}^{2}\right]<\infty$ at stationarity, $\nu^{\left(k\right)}$ converges almost surely to a constant and hence the SGD bias analysis also applies to avgRMSProp and avgAdam.

The second problem is that Proposition 6 only holds for the mean-field Gaussian variational family. However, other variational families such as normalizing flows are of substantial practical interest. Therefore, we consider the weaker assumption that either Eq. (2) holds *or* there exist constant vectors Λ, $A\in R^{m}$ and a constant κ∈[1/2,1) such that

(5) $${\bar{\lambda }}_{\gamma }={\text{λ}}_{\text{*}}+{\Lambda \gamma }^{\kappa }+A\text{γ}+o\left({\text{γ}}^{2\kappa }\right).$$

Adding the latter assumption, we have the following generalization of Proposition 6, which holds for any sufficiently regular variational family:

**Proposition 7** *Let* 𝒬 *be the variational approximation family. If (i)* Eq. (5) *holds for some* κ∈[1/2,1) *or* Eq. (2) *holds (in which case let κ = 1), (ii)* $\gamma^{\prime }=O(\gamma )$, *and (iii) for all* $\theta \in R^{d}$, $log\;q_{\lambda }(\theta )$ *is three-times continuously differentiable with respect to* λ, *then for some* C≥0,

$$\text{SKL}\left(q_{\gamma *},q_{\gamma^{\prime }*}\right)=C{\left\{{\text{γ}}^{\kappa }-{\left(\gamma^{\prime }\right)}^{\kappa }\right\}}^{2}+o({\text{γ}}^{2\kappa }).$$

*Moreover, C depends on only* $\lambda_{*}$ *and either* Λ *(if* κ∈[1/2,1)) *or* *A* *(if* κ=1*)*.

See Appendix A.2 for the proof. Using Proposition 7 and omitting $o\left(\gamma^{2\kappa }\right)$ terms, we have $\delta_{\gamma }\approx C\gamma^{2\kappa }{\left(1/\rho^{\kappa }-1\right)}^{2}$. To improve the reliability of the estimates based on Proposition 7, we propose to use the symmetrized KL estimates between the variational approximations obtained at successive fixed learning rates. Let $\gamma_{0}$ denote the initial learning rate, so that after *t* learning rate decreases, the learning rate is $\gamma_{t}:=\gamma_{0}\rho^{t}$. Let $\delta_{t}:=SKL\left(q_{\gamma_{t}*},q_{\gamma_{t-1}*}\right)$ and assume the current learning rate is $\gamma_{T}$.

Depending on the optimization algorithm, we can estimate *κ* (or set *κ* = 1 if using modified adaptive SGD algorithm with a mean-field Gaussian variation family) and *C* using a regression model of the form

(6) $$\text{log}\;\delta_{t}=\text{log}\;C+2\;\text{log}\left(1/\rho^{\kappa }-1\right)+2\kappa \;\text{log}\gamma_{t}+\eta_{t},\;t=1,\ldots ,T,$$

where $\eta_{t}∼𝒩\left(0,\sigma^{2}\right)$. Given the estimate $\overset{ˆ}{C}$ for *C* and the estimate $\overset{ˆ}{\kappa }$ for *κ* (or $\overset{ˆ}{\kappa }=1$) , we obtain the estimated relative SKL,

$$\hat{\text{RSKL}}=\rho^{\hat{k}}+\frac{\xi }{{\hat{C}}^{1/2}\gamma_{t}^{\hat{k}}}.$$

Because we use the regression model in Eq. (6) in a low-data setting, we place (weak) priors on log *C* and *σ*:

$$\text{log}\;C∼\text{Cauchy}(0,10),\;\sigma ∼{\text{Cauchy}}^{+}(0,10),$$

where Cauchy^+^ is the Cauchy distribution truncated to nonnegative values. If we use an adaptive stochastic optimization algorithm then we also place a prior on *κ*:

κ∼Unif(0,1).

Also, because we expect early SKL estimates to be less informative about *C* (and *κ*) due to the influence of $o\left(\gamma^{2\kappa }\right)$ terms, we use a weighted regression with the likelihood term for ($\delta_{t},\gamma_{t}$) having weight

(7) $$w_{t}={\left\{1+{\left(T-t\right)}^{2}/3^{2}\right\}}^{-1/4}.$$

The weight formula enables the amplification of the significance of the most recent observations, with down-weighting becomes more significant after there are about 3 additional observations. On the other hand, the power of 1/4 ensures a gradual reduction in weight, preventing a steep drop-off in importance.

**Figure 3:**
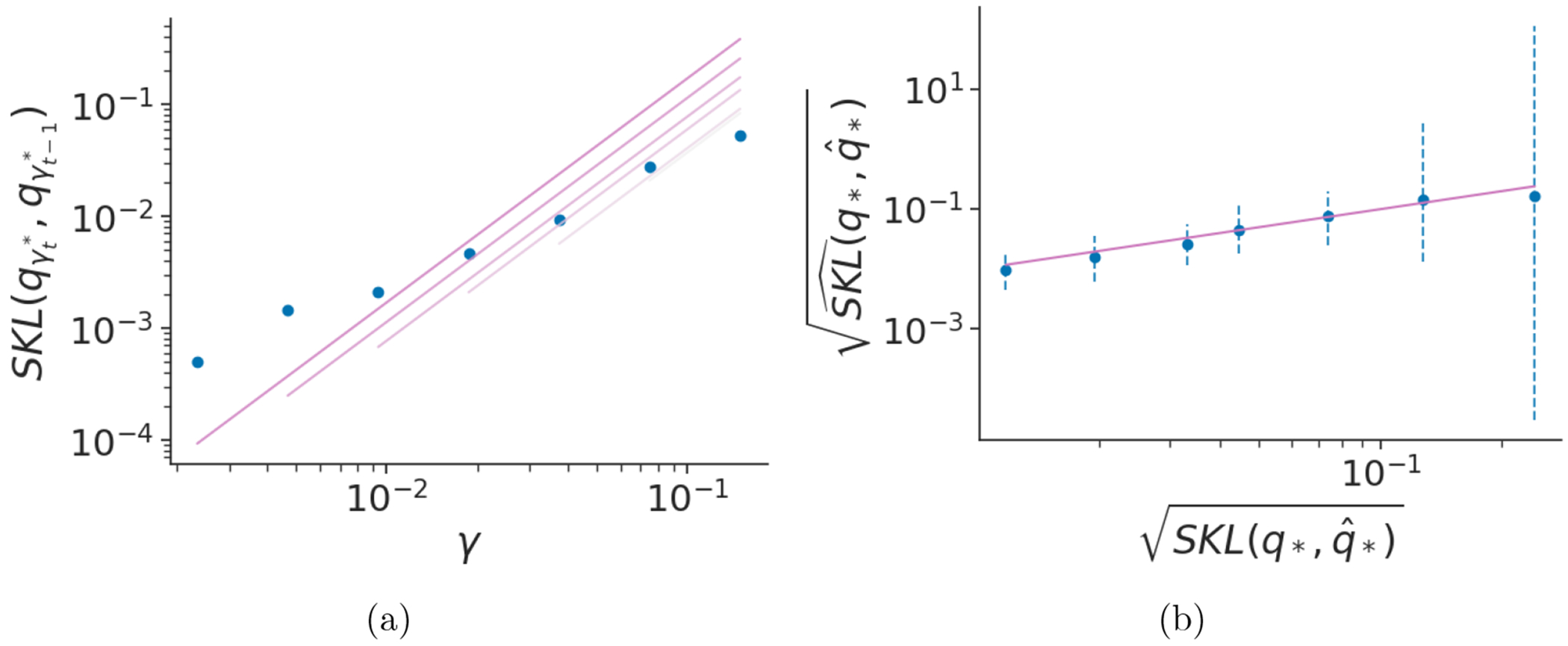
Results for estimating the symmetrized KL divergence with avgAdam in the case of a Gaussian distribution 𝒩(0,V) with *d* = 100 and $V_{ij}=j𝟙[i=j]$ (diagonal non-identity covariance). **(a)** Learning rate versus symmetrized KL divergence of adjacent iterate averaged estimates of the optimal variational distribution. The lines indicate the linear regression fits, with setting *κ* = 1. **(b)** Square root of true symmetrized KL divergence versus the estimated value with 95% credible interval. The uncertainty of the estimates decreases and remains well-calibrated as the learning rate decreases.

We use the posterior mean(s) to estimate $\overset{ˆ}{C}$ (and $\overset{ˆ}{\kappa }$). Figure 3 validates that, in the case of avgAdam, the log of the learning rate and symmetrized KL divergence have approximately a linear relationship and that our regression approach to estimating *C* leads to reasonable estimates of $SKL\left(q_{\gamma *},q_{*}\right)$. See Fig. C.2 for similar results for other target distributions with avgAdam.

**Figure 4:**
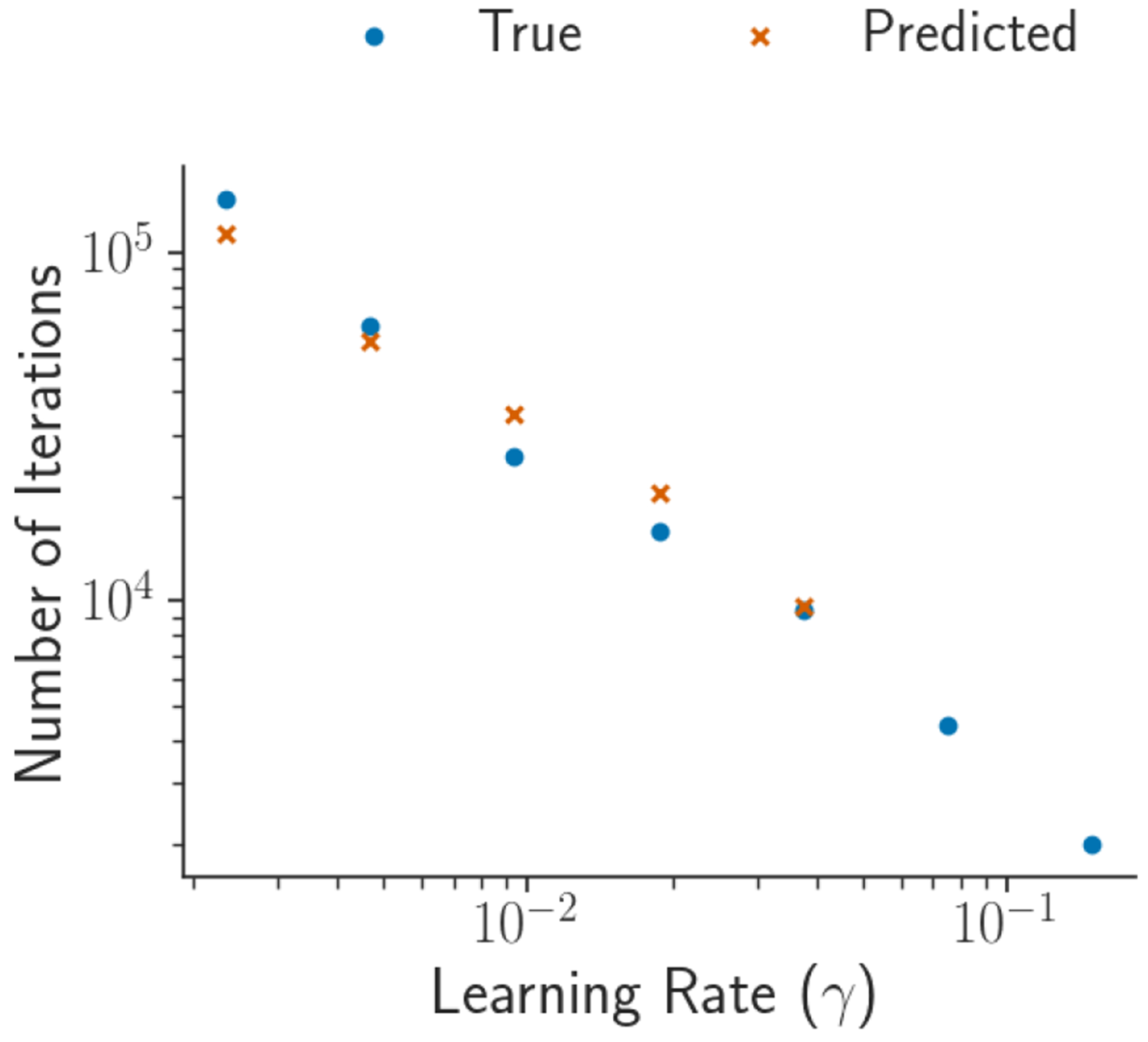
Results for predicting the number of iterations needed to reach convergence at each learning rate decrease in the case of Gaussian distribution 𝒩(0,V) with *d* = 100 and $V_{ij}=j𝟙[i=j]$ (diagonal non-identity covariance). The blue points (orange crosses) represent the true (predicted) number of iterations needed to reach convergence.

To estimate the relative iteration increase RI, we need to estimate the number of iterations to reach convergence at the next learning rate $\gamma_{t+1}$. It is reasonable to assume that there is exponential growth in the number of iterations to reach convergence as the learning rate decreases since stochastic gradient algorithms to converge at a polynomial rate (Bubeck, 2015). Recall that $K_{\gamma_{t}}$ is the number of iterations to reach convergence at the current learning rate. We fit a weighted least square regression model of the form

(8) $$\text{log}\;K_{\gamma_{t}}=\alpha \;\text{log}\;\gamma_{t}+\beta +\nu_{t},\;t=1,\ldots ,T,$$

where $\nu_{t}∼𝒩\left(0,\sigma_{t}^{2}\right)$. We then use the coefficient estimates $\overset{ˆ}{\alpha }$ and $\overset{ˆ}{\beta }$ to predict the number of iterations required for convergence at the next learning rate to be ${\hat{K}}_{\gamma_{t+1}}:=\gamma_{t+1}^{\overset{ˆ}{\alpha }}e^{\overset{ˆ}{\beta }}$. We use the same weights given in Eq. (7) for observations of the regression model due to the non-linear behavior of the earlier convergence iterate estimates. Figure 4 demonstrates that linear relationship in Eq. (8) does in fact hold and that our weighted least square regression model predicts the number of convergence iterations $K_{\gamma_{t}}$ quite accurately. The estimated relative iterations is then $\hat{RI}={\hat{K}}_{\gamma_{t+1}}/\left(K_{\gamma_{t}}+K_{0}\right)$.

**Figure 5:**
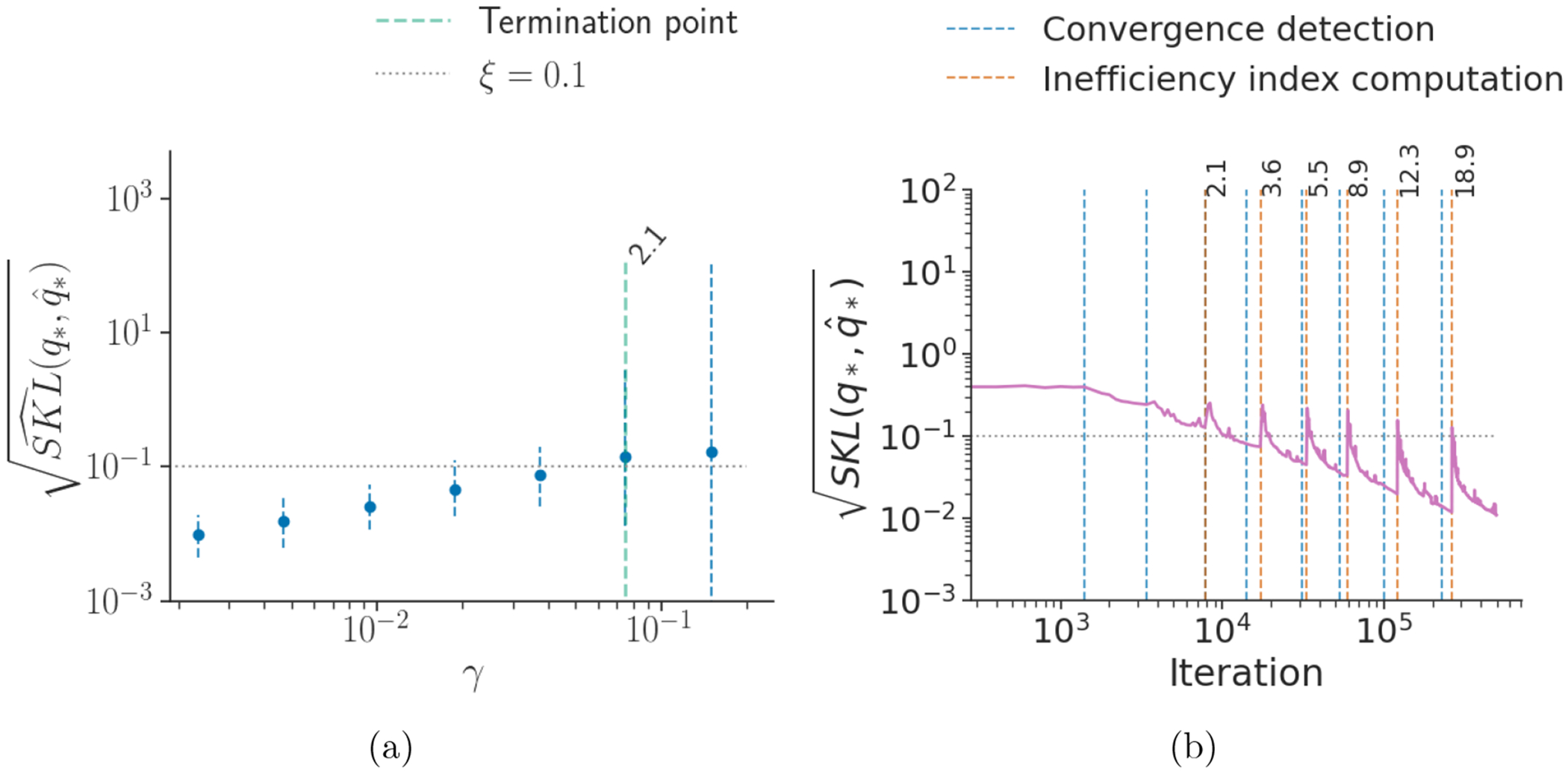
Results for the termination rule trigger point in the case of a Gaussian distribution 𝒩(0,V) with *d* = 100 and $V_{ij}=j𝟙[i=j]$ (diagonal non-identity covariance). **(a)** Learning rate versus square root of estimated symmetrized KL divergence with 95% credible interval (dashed blue line). The green vertical line indicates the termination rule trigger point with the corresponding $\overset{ˆ}{ℐ}$ value. **(b)** Iterations versus square root of symmetrized KL divergence between iterate average and optimal variational approximation. The vertical lines indicate the convergence detection points using $\overset{ˆ}{R}$ (blue) and inefficiency index computation ($\overset{ˆ}{ℐ}$) points (orange) with corresponding values.

Using the estimates $\hat{RSKL}$ and $\hat{RI}$ we obtain the termination rule $\hat{ℐ}=\hat{RSKL}\times \hat{RI}>\tau$. Figure 5 shows that when the user chosen target accuracy ξ=0.1, the termination rule triggers when the square root of the symmetrized KL divergence is approximately equal to ξ. Figures C.3 to C.5 shows similar results of other Gaussian targets and posteriordb models and data sets (see Section 7.2 for details).

## 5. Learning Rate Scheduler

For a fixed learning rate, computing the iterate average ${\overset{ˆ}{\lambda }}_{\gamma }$ defined in Eq. (3) requires determining the iteration $k^{\text{conv}}$ at which stationarity is reached and the number of iterations $k^{\text{avg}}$ to use for computing the average. We address each of these in turn.

### 5.1 Detecting Convergence to Stationarity

We investigate two approaches to detecting stationarity: the SASA+ algorithm of Zhang et al. (2020) and the $\hat{R}$-based criterion from Dhaka et al. (2020). We make several adjustments to both approaches to reduce the number of tuning parameters and to make the remaining ones more intuitive. In our empirical findings, we have observed that the $\hat{R}$ criterion outperforms the SASA+ criterion. Therefore, we describe the former here and the latter in Appendix B.3.

Let $\hat{R}(\lambda_{i}^{\left(k-W+1\right)},\ldots ,\lambda_{i}^{\left(k\right)})$ denotes the split-$\hat{R}$ of the *i*th component of the last *W* iterates and define

$${\hat{R}}_{\text{max}}(W):=\underset{1\leq i\leq m}{\text{max}}\hat{R}\left(\lambda_{i}^{\left(k-W+1\right)},\ldots ,\lambda_{i}^{\left(k\right)}\right).$$

An ${\hat{R}}_{\text{max}}(W)$ value close to 1 indicates the last *W* iterates are close to stationarity. In MCMC applications having ${\hat{R}}_{\text{max}}(W)\leq 1.01$ is desirable (Vats and Knudson, 2021; Vehtari et al., 2021). Dhaka et al. (2020) uses the weaker condition ${\hat{R}}_{max}(W)\leq 1.1$ since iterate averaging does not require the same level of precision as MCMC. Dhaka et al. (2020) take the window size *W* = 100, but in more challenging and high-dimensional problems a fixed smaller *W* is insufficient. Therefore, we instead search over window sizes between a minimum window size *W*_min_ and 0.95*k* to find the one that minimizes ${\hat{R}}_{max}(W)$. The minimum window size is necessary to ensure the $\hat{R}$ values are reliable. We use the upper bound 0.95*k* to always allow a small amount of “warm-up” without sacrificing more than 5% efficiency. Therefore, we estimate $W_{\text{opt}}=arg{min}_{W_{min}\leq W\leq 0.95k}{\hat{R}}_{max}(W)$ using a grid search over 5 equally spaced values ranging from $W_{min}$ to 0.95*k* and require ${\hat{R}}_{max}\left(W_{\text{opt}}\right)\leq 1.1$ as the stationarity condition.

**Figure 6:**
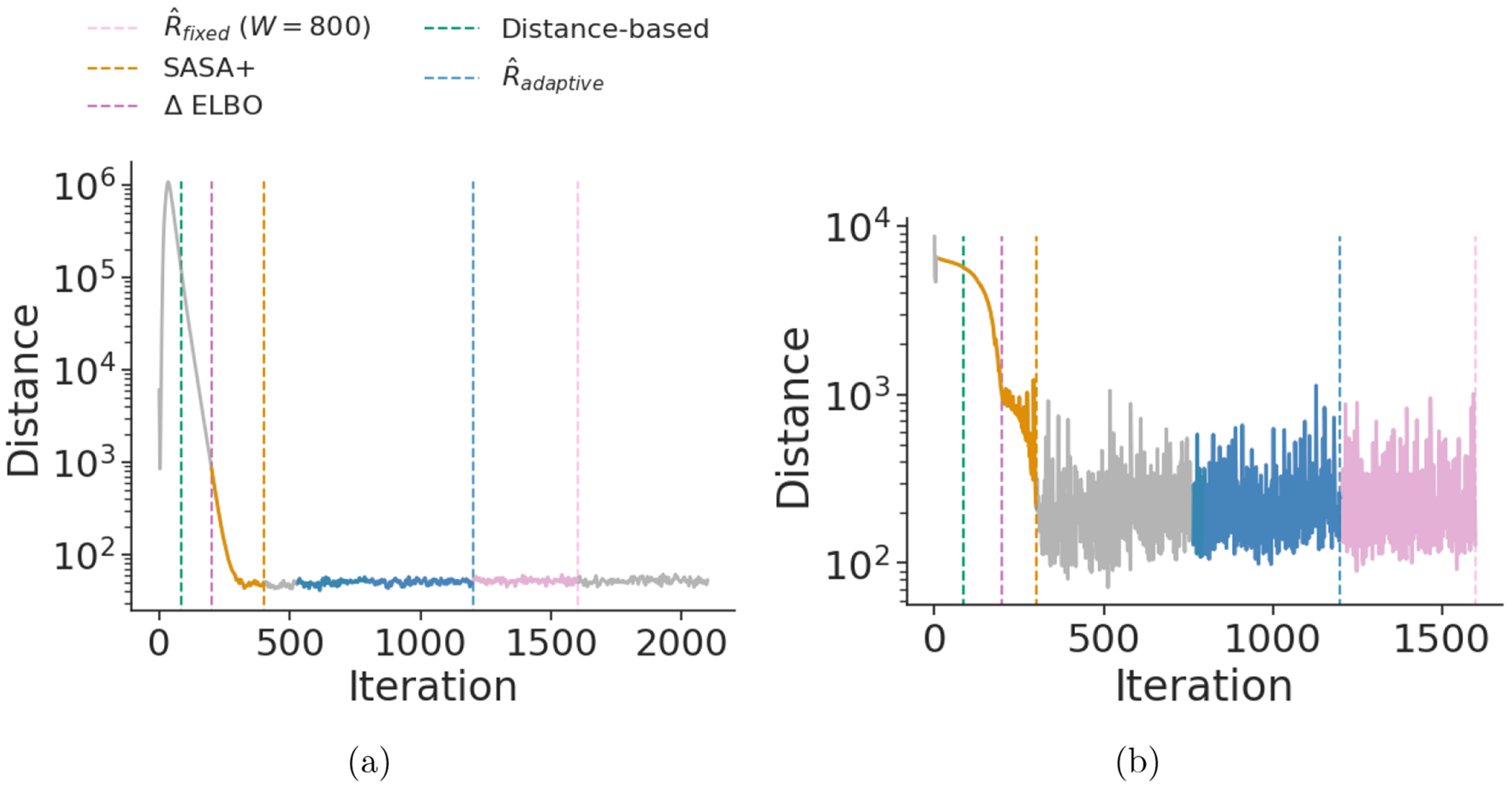
Iteration number versus distance between iterate average and current iterate. The vertical lines indicate convergence detection trigger points and (for SASA+ and $\hat{R}$) the colored portion of the accuracy values indicate they are part of the window used for convergence detection. **(a)** An uncorrelated Gaussian distribution 𝒩(0,V) with *d* = 500 and *V* = *I*. **(b)** A posteriordb data set/model *mcycle_gp* with *d* = 66.

**Figure C.1:**
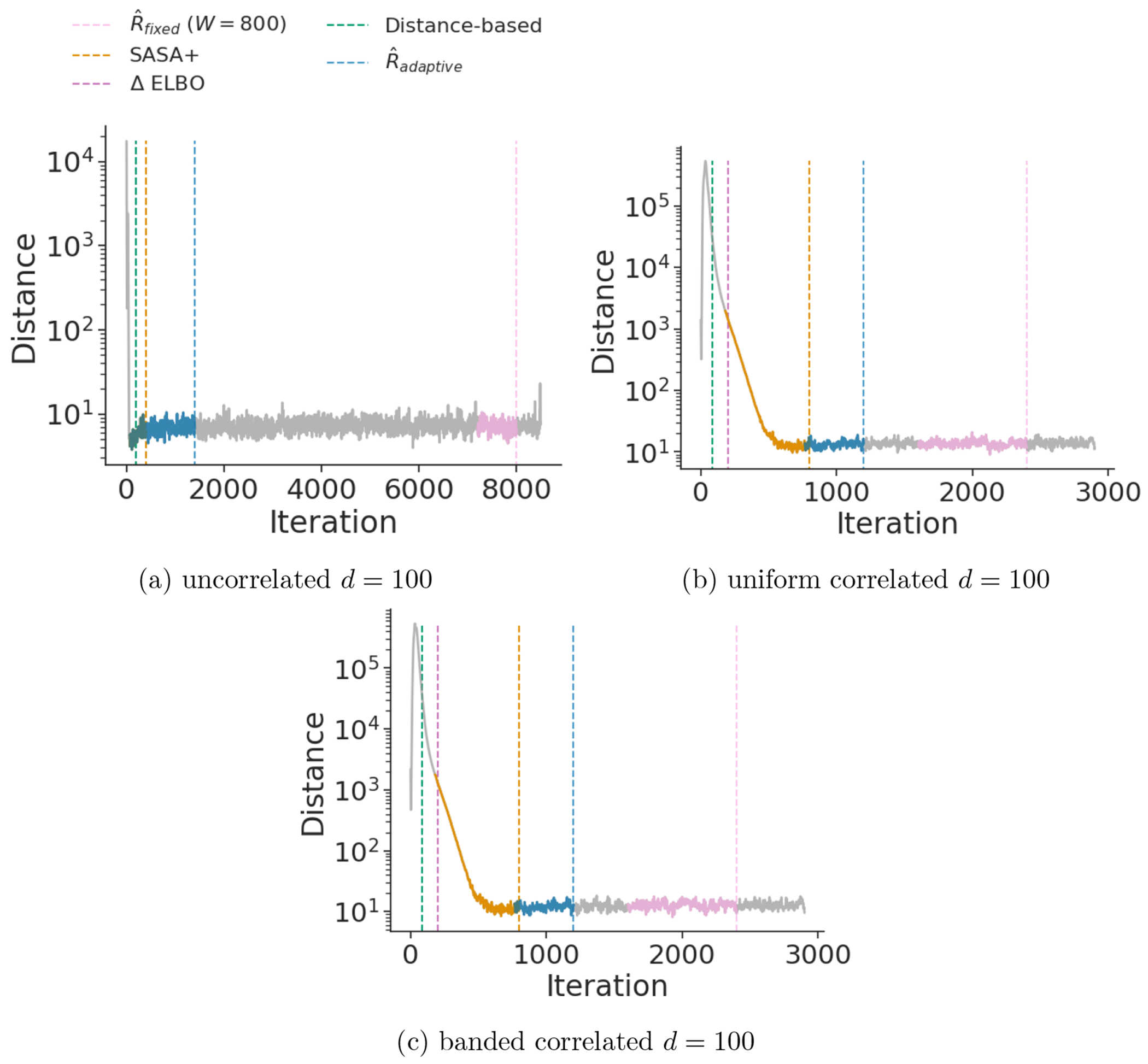
Iteration number versus distance between iterate average and current iterate for Gaussian targets. The vertical lines indicate convergence detection trigger points and (for SASA+ and $\hat{R}$) the colored portion of the accuracy values indicate they are part of the window used for convergence detection.

**Figure C.2:**
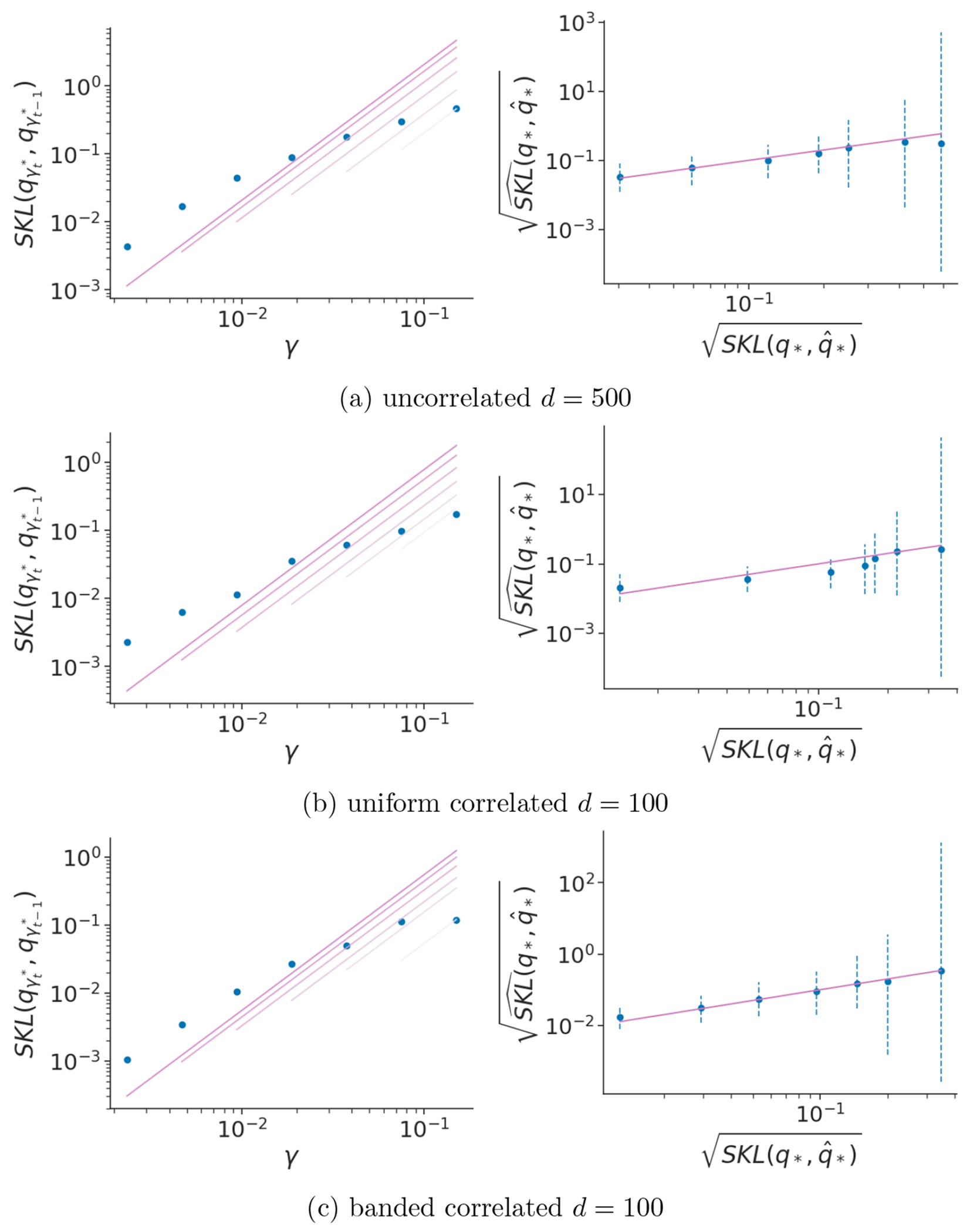
Results for estimating the symmetrized KL divergence with avgAdam. **(left)** Learning rate versus symmetrized KL divergence of adjacent iterate averaged estimates of optimal variational distribution. The lines indicate the linear regression fits, with setting *κ* = 1. **(right)** Square root of true symmetrized KL divergence versus the estimated value with 95% credible interval. The uncertainty of the estimates decreases and remains well-calibrated as the learning rate decreases.

**Figure C.3:**
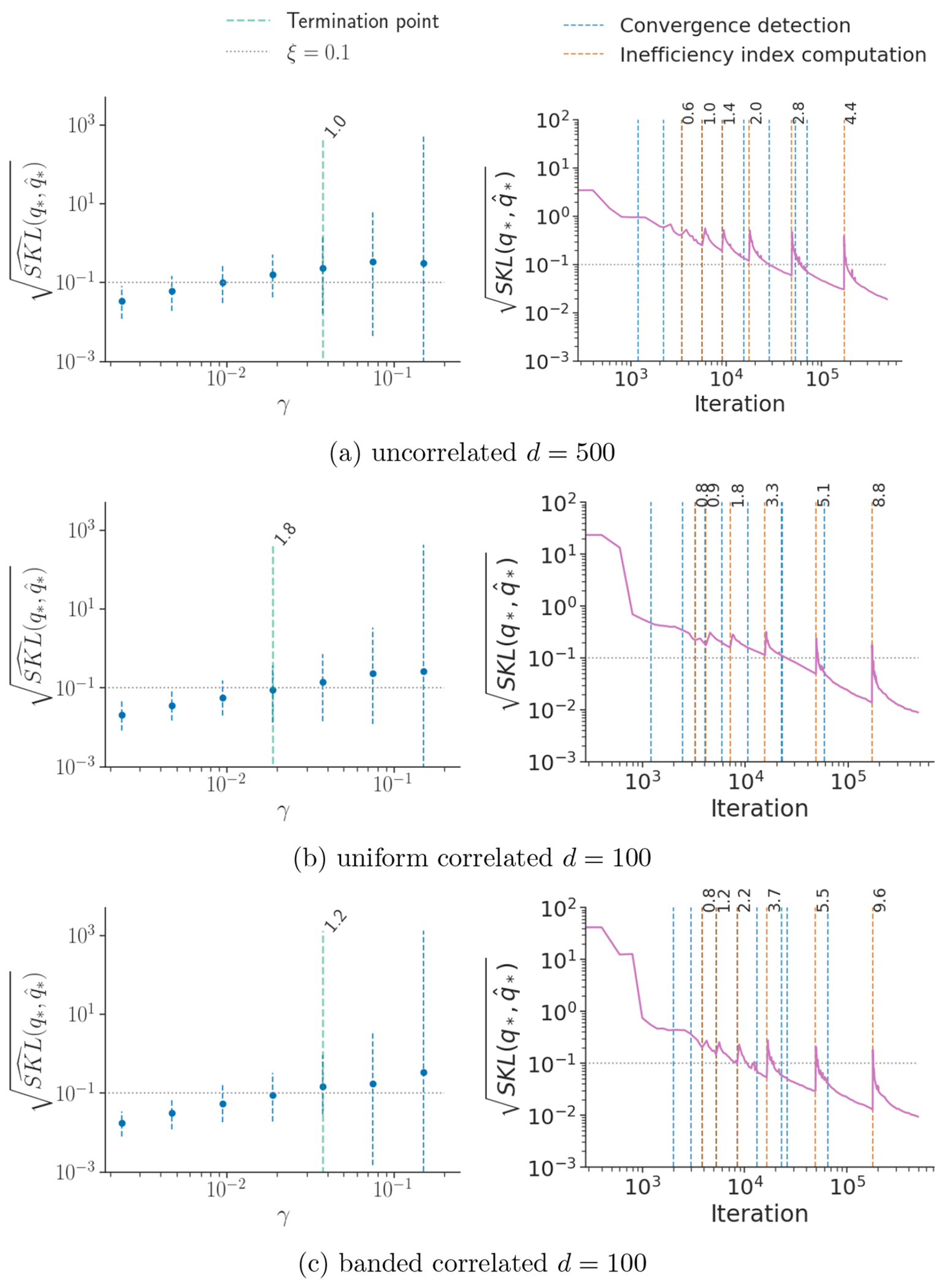
Results of termination rule trigger point of Gaussian targets. **(left)** Learning rate versus square root of estimated symmetrized KL divergence with 95% credible interval (dashed blue line). The green vertical line indicates the termination rule trigger point with corresponding $\overset{ˆ}{ℐ}$ value. **(right)** Iterations versus square root of symmetrized KL divergence between iterate average and optimal variational approximation. The vertical lines indicate the convergence detection points using $\overset{ˆ}{R}$ (blue) and inefficiency index computation $\left(\overset{ˆ}{ℐ}\right)$ points (orange) with corresponding values.

**Figure C.6:**
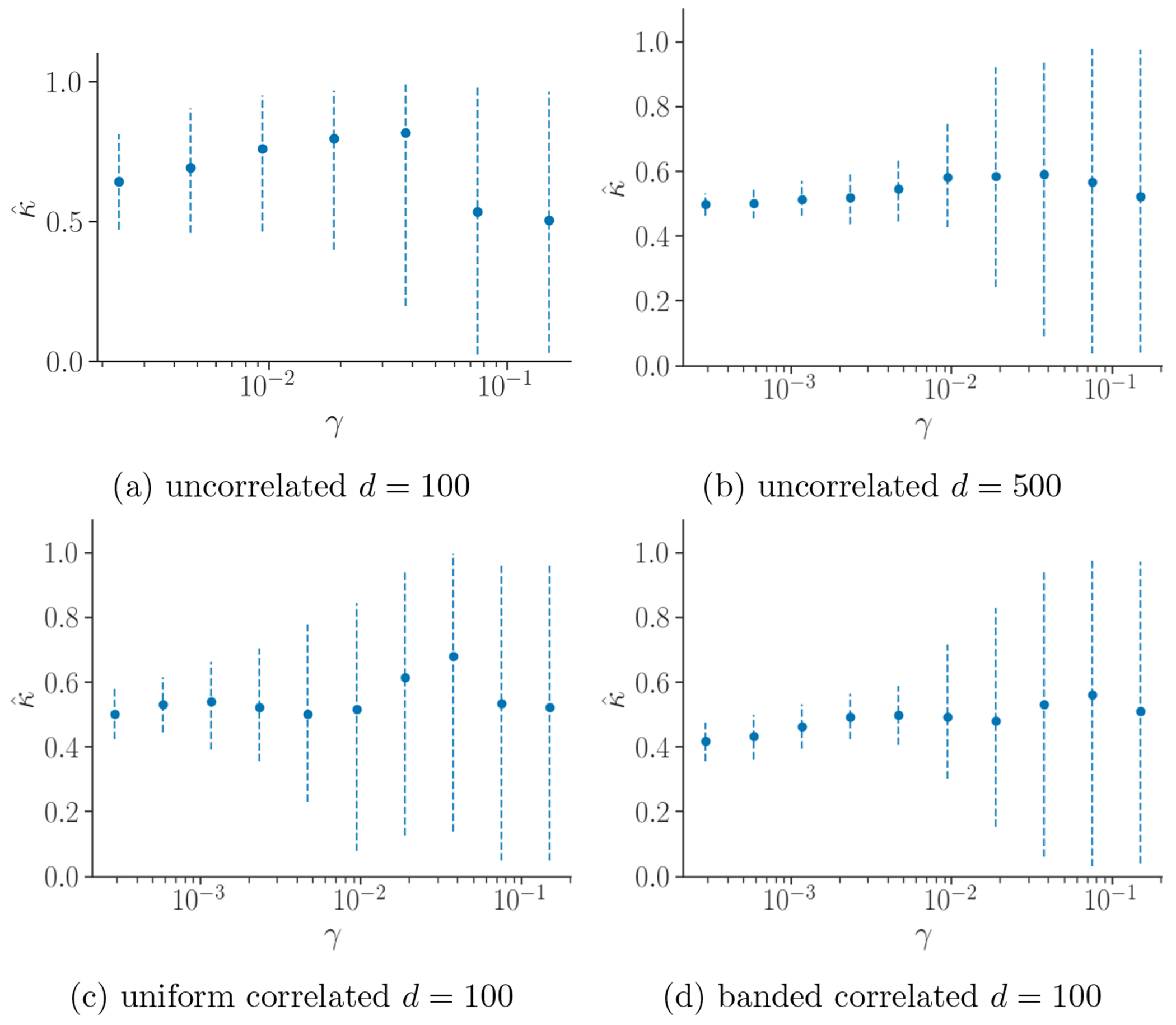
Learning rate versus $\overset{ˆ}{\kappa }$ for Gaussian targets using RMSProp with 95% credible interval. The estimates suggestion κ is approximately 0.5.

**Figure C.7:**
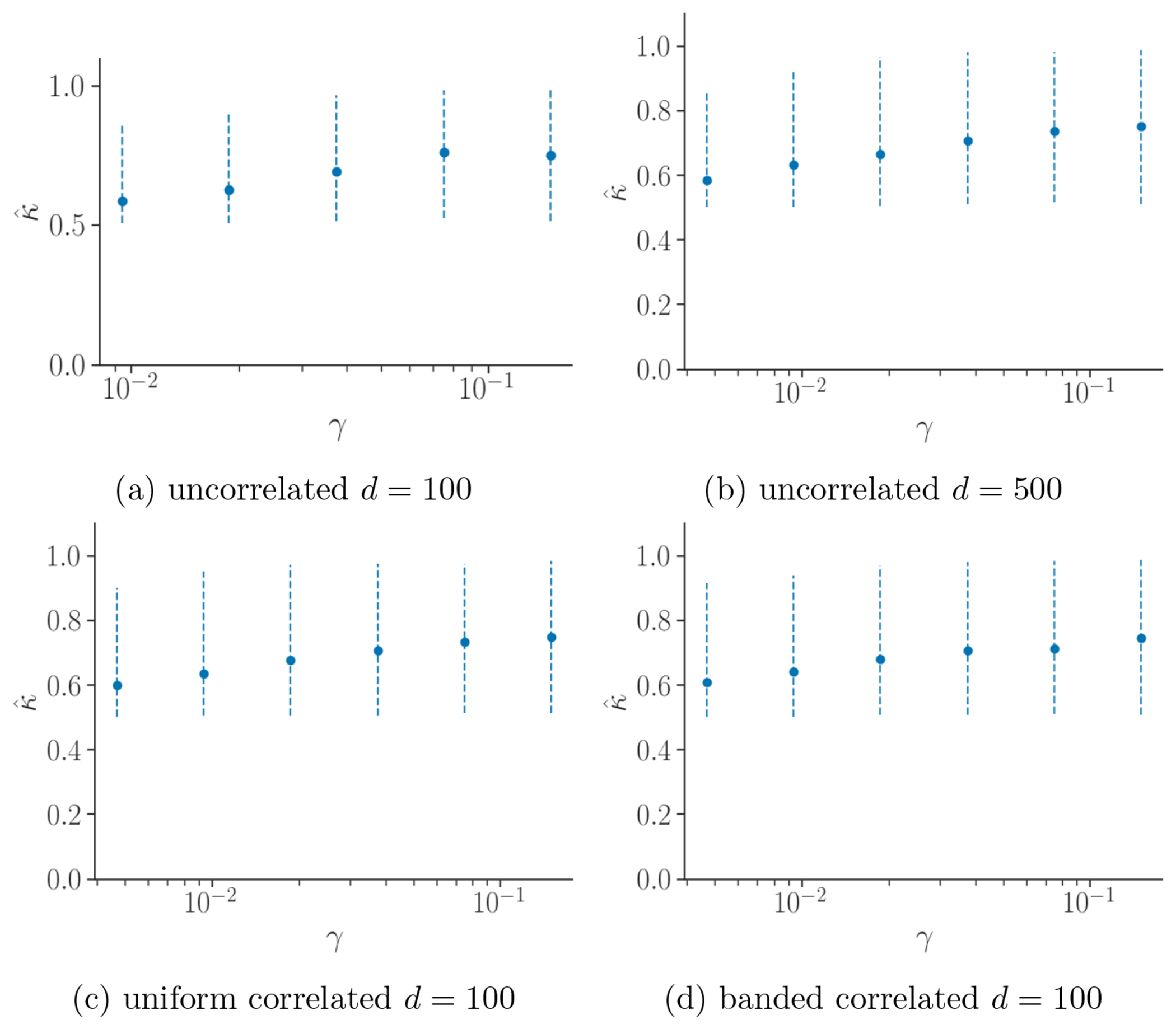
Learning rate versus $\overset{ˆ}{\kappa }$ for Gaussian targets using Adam with 95% credible interval. The estimates suggest κ is less than 0.8, with all point estimates close to 0.6.

Figure 6 compares our adaptive SASA+ and adaptive $\hat{R}$ criteria to the criterion used in Dhaka et al. (2020) with a fixed window size of *W* = 800 and ΔELBO rule from Kucukelbir et al. (2015), which is used Stan’s ADVI implementation (cf. the results of Dhaka et al. (2020)). We do not use *W* = 100 as is done by Dhaka et al. (2020) because it was too small to detect convergence. Additionally, Fig. 6 compares to another convergence detection approach proposed by Pesme et al. (2020) (described in Appendix B.4), where they use a distance-based statistic to detect convergence. While adaptive SASA+, ΔELBO, fixed window size $\hat{R}$, and the distance-based statistic approach sometimes trigger too early or too late or SASA+ use iterations before it reaches the convergence, adaptive $\hat{R}$ consistently triggers when the full window suggests convergence has been reached. See Fig. C.1 for additional Gaussian target examples.

### 5.2 Determining the Number of Iterates for Averaging

After detecting convergence to stationarity, we need to find $k^{\text{avg}}$ large enough to ensure the iterative average is sufficiently close to the mean ${\overset{‾}{\lambda }}_{\gamma t}$. But what is close enough? Building on our discussion in Section 3, we aim to ensure the error in the variational parameter estimates are small relative to the scale of uncertainty. For mean-field Gaussian distributions, the following result allows us to make such a guarantee precise.

**Proposition 8** *Let* 𝒬 *be the family of mean-field Gaussian distributions. Let* $\overset{ˆ}{\lambda }=(\overset{ˆ}{\tau },\overset{ˆ}{\psi })$ *denote an approximation to* $\overset{‾}{\lambda }=(\overset{‾}{\tau },\overset{‾}{\psi })$. *Define* $\overset{ˆ}{\sigma }:=exp(\overset{ˆ}{\psi })$ *and* $\overset{‾}{\sigma }:=exp(\overset{‾}{\psi })$. *If there exists* ε∈(0,1/2) *such that* $\left|{\overset{ˆ}{\tau }}_{i}-{\overset{‾}{\tau }}_{i}\right|\leq \varepsilon {\overset{ˆ}{\sigma }}_{i}$ *and* $\left|{\overset{ˆ}{\psi }}_{i}-{\overset{‾}{\psi }}_{i}\right|\leq \varepsilon$, *then*

$$\frac{\left|{\hat{\sigma }}_{i}-{\bar{\sigma }}_{i}\right|}{{\bar{\sigma }}_{i}}\leq 1.5\varepsilon \;and\;\frac{\left|{\hat{\tau }}_{i}-{\bar{\tau }}_{i}\right|}{{\bar{\sigma }}_{i}}\leq 1.75\varepsilon .$$

See Appendix A.4 for the proof. Based on Proposition 8, for mean-field Gaussian variational families we use the iterate average once the mean MCSEs $d^{-1}\sum_{i=1}^{d}MCSE\left({\overset{ˆ}{\tau }}_{\gamma ,i}\right)/{\overset{ˆ}{\sigma }}_{\gamma ,i}$ and $d^{-1}\sum_{i=1}^{d}MCSE({\overset{ˆ}{\psi }}_{\gamma ,i})$ are less than *ε*.For other variational families we rely on the less ε rigorous condition that $m^{-1}\sum_{i=1}^{m}MCSE({\overset{ˆ}{\lambda }}_{\gamma ,i})$ is less than ε. We also require the effective sample sizes of all parameters to be at least 50 to ensure the MCSE estimates are reliable.

Because the MCSE check requires computing *d* ESS values, it can be computationally expensive, especially for high-dimensional models. Therefore, it is important to optimize when conducting the checks.A well-known approach in such situations is the “doubling trick.” Let $W_{\text{conv}}$ denote the window size when convergence is detected, and Let $W_{\text{conv}}$ denote the window size when convergence is detected, and let $W_{\text{opt}}$ denote the minimal window size that satisfies the MCSE check. The doubling trick would suggest checking at iteration numbers $k^{\text{conv}}+2^{j}W_{\text{conv}}$ for j=0,1,…, in which case the total computational cost is within a factor of $4\;log\;W_{\text{opt}}$ of the optimal scenario in in which the check is only done at $k^{\text{conv}}+W_{\text{conv}}$ and $\left.k^{\text{conv}}+W_{\text{opt}}\right)$. However, we can potentially do substantially better by accounting for the different computational cost of the optimization versus the MCSE check.

**Proposition 9** *Assume that the cost of the MCSE check using* *K* *iterates is* $C_{E}K$ *and the cost of* *K* *iterations of optimization is* $C_{O}K$. *Let* $r:=C_{O}/C_{E}$, $\chi (r):=1+(1+r{)}^{-1/2}$, *and* $g(r):=(2+r+2(1+r{)}^{1/2})/(1+r)$. *If the MCSE check is done on iteration numbers* $k^{\text{conv}}+\chi (r{)}^{j}W_{\text{conv}}$ *for* j=0,1,…, *then the total computational cost will be within a factor of* g(r) *of optimal*.

See Appendix A.5 for the proof. Since g(0)=4 and g(r) is monotonically decreasing in *r*, when r≈0; that is, $C_{O}$ is negligible compared to $C_{E}$, we recover the doubling rule since χ(0)=2. However, as long as *r* is significantly greater than zero, the worst-case additional cost factor can be substantially less than 4. Therefore we carry out the MCSE check on iteration numbers $k^{\text{conv}}+\chi (r{)}^{j}W_{\text{conv}}$ with *r* estimated based on the actual runtimes of the optimization so far and the first MCSE check.

## 6. Complete Framework

Combining our innovations from Sections 4 and 5 leads to our complete framework. When γ is fixed, our proposal from Section 5 is summarized in Algorithm 1, which we call *fixed–learning-rate automated stochastic optimization* (FASO). Combining the termination rule from Section 4 with FASO, we get our complete framework, *robust and automated black-box variational inference (RABVI)*, which we summarize in Algorithm 2. We will verify the robustness of RABVI through numerical experiments. RABVI is automatic since the user is only required to provide a target distribution and the only tuning parameters we recommend changing from their defaults are defined on interpretable, intuitive scales:

- **accuracy threshold** ξ**:** The symmetrized KL divergence accuracy threshold can be set based on the expected accuracy of the variational approximation. If the user expects $KL\left(\pi |q_{*}\right)$ to be large, then we recommend choosing ξ∈[1,10]. If the user expects $KL\left(\pi |q_{*}\right)$ to be fairly small, then we recommend choosing ξ∈[.01,1]. Our experiments suggest ξ=0.1 is a good default value.
- **inefficiency threshold** *τ***:** We recommend setting the inefficiency threshold τ=1, as this weights accuracy and computation equally. A larger value (for example, 2) could be chosen if accuracy is more important while a smaller value (for example, 1/2) would be appropriate if computation is more of a concern.
- **maximum number of iterations** $K_{max}$**:** The maximum number of iterations can be set by the user based on their computational budget. RABVI will warn the user if the maximum number of iterations is reached without convergence, so the user can either increase $K_{max}$ or accept the estimated level of accuracy that has been reached.

We expect the remaining tuning parameters will typically not be adjusted by the user. We summarize our recommendations:

- **initial learning rate** $\gamma_{0}$**:** When using adaptive methods such as RMSProp or Adam, the initial learning rate can essentially be set in a problem-independent manner. We use $\gamma_{0}=0.3$ in all of our experiments. If using non-adaptive methods, a line search rule such as the one proposed in Zhang et al. (2020) could be used to find a good initial learning rate.
- **minimum window size** $W_{\text{min}}$**:** We recommend taking $W_{\text{min}}=200$ so that that each of the split-$\hat{R}$ values are based on at least 100 samples.
- small iteration number $K_{0}$**:** The value of $K_{0}$ should represent a number of iterations the user considers to be fairly small (that is, not requiring too much computational effort). We use $K_{0}=5W_{min}=1000$ for our experiments, but it could be adjusted by the user.
- **initial iterate average relative error threshold** $\varepsilon_{0}$**:** We recommend scaling $\varepsilon_{0}$ with ξ since more accurate iterate averages are required for sufficiently accurate symmetrized KL estimates. Therefore, we take $\varepsilon_{0}=\xi$ by default.
- **adaptation factor** *ρ***:** We recommend taking *ρ* = 0.5 because using a smaller *ρ* value could lead to too few $\delta_{t}$ values for the estimation of *C* (and *κ*) and using a larger *ρ* value would make the algorithm too slow.
- **Monte Carlo samples** *M***:** We find that *M* = 10 provides a good balance between gradient accuracy and computational burden but the performance is fairly robust to the choice of *M* as long as it is not too small.

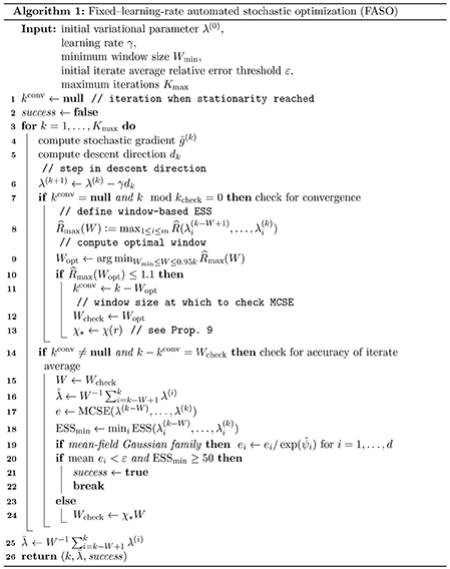



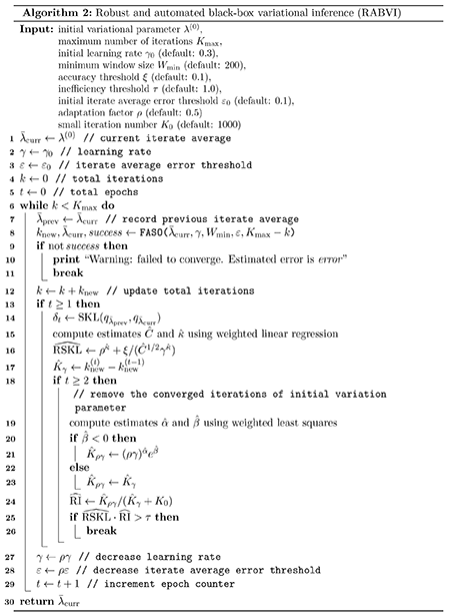


## 7. Experiments

Unless stated otherwise, all experiments use avgAdam to compute the descent direction, mean-field Gaussian distributions as the variational family, and the tuning parameters values recommended in Section 6. We fit the regression model for *C* (and *k*) in Stan, which result in extremely small computational overhead of less than 0.5%. We compare RABVI to FASO, Stan’s ADVI implementation, SGD using an exponential decay of the learning rate, and fixed-learning rate versions of RMSProp, Adam, and a windowed version of Adagrad (WAdagrad), which is the default optimizer in PyMC3. Moreover, we compare RABVI with exponential decay and cosine learning rate schedules using Adam and RMSProp optimization methods. We run all the algorithms that do not have a termination criterion for $K_{max}=100,000$ iterations and for the fixed–learning-rate algorithms we use learning rate γ=0.01 in an effort to balance speed with accuracy. For exponential decay, we use a learning rate of $\gamma =\gamma_{0}\delta^{\left\lfloor k/s\right\rfloor }$, where $\gamma_{0}$ denotes the initial learning rate, δ denotes the decay rate, *k* denotes the iteration, and *s* denotes the decay step. We choose $\gamma_{0}=0.01$, δ=0.96, and s=900 so that the final learning rate is approximately 0.0001 (Chen et al., 2017). For cosine schedule, we use a learning rate of $\gamma =\gamma_{\text{min}}+\frac{1}{2}(\gamma_{\text{max}}-\gamma_{\text{min}})(1+cos(\frac{k}{K}\pi ))$, where $\gamma_{\text{min}}$ and $\gamma_{\text{max}}$ denote the minimum and maximum values of learning rate, *k* denotes the current iteration, and *K* denotes the maximum number of iterations (Loshchilov and Hutter, 2017). We choose $\gamma_{min}=0.0001$, $\gamma_{max}=0.01$ to make it comparable with other methods.

We use symmetrized KL divergence as the accuracy measure when we can compute the ground-truth optimal variational approximation. Otherwise, we use the following metrics (where μ and σ are, respectively, the posterior mean and standard deviation vectors):

- *Relative mean error* $\|(\mu -\overset{ˆ}{\mu })/\sigma {\|}_{2}$, where $\overset{ˆ}{\mu }$ is the variational approximation to μ.
- *Relative standard deviation error* $\|\overset{ˆ}{\sigma }/\sigma -1{\|}_{2}$, where $\overset{ˆ}{\sigma }$ is the variational approximation to σ.
- *Under coverage error* of the variational approximation to the 95% credible intervals $min\left(0,\left|.95-c_{i}\right|\right)$, where $c_{i}:=\Pi \left(\left\{\theta :\theta_{i}\in \left(a_{i},b_{i}\right)\right\}\right)$ and $\left(a_{i},b_{i}\right)$ is the variational estimate of the central 95% credible interval for parameter $\theta_{i}$.
- *Maximum mean discrepancy (MMD)* ${MMD}^{2}(P,Q):=E\left[k\left(x,x^{\prime }\right)-2k(x,y)+k\left(y,y^{\prime }\right)\right]$, where $x,x^{\prime }∼P$ and $y,y^{\prime }∼Q$ are independent and $k(x,y)=exp\left\{-\frac{1}{2}{\left\|\frac{x-y}{\sigma }\right\|}_{2}^{2}\right\}$ is the squared exponential kernel (Gretton et al., 2006).

### 7.1 Accuracy with Gaussian Targets

First, to explore optimization accuracy relative to the optimal variational approximation, we consider Gaussian targets of the form π=𝒩(0,V). In such cases, we can compute the ground-truth optimal variational approximation either analytically (because the distribution belongs to the mean-field variational family and hence $q_{*}=\pi$) or numerically using deterministic optimization (since the KL divergence between Gaussians is available in closed form). Specifically, we consider the following covariances that aid in assessing our framework across a range of condition numbers from 1 to around 9000:

- Identity covariance: V=I
- Diagonal non-identity covariance: $V_{ij}=j𝟙[i=j]$
- Uniform covariance with correlation $0.8:V_{ij}=𝟙[i=j]+0.8𝟙[i\neq j]$
- Banded covariance with maximum correlation $0.8:V_{ij}=𝟙[i=j]+0.8^{\left|i-j\right|}𝟙[i\neq j]$
- Diagonal non-identity banded covariance with maximum correlation $0.8:V_{ij}=j𝟙[i=j]+0.8^{\left|i-j\right|}𝟙[i\neq j]$
- Diagonal identity (except first entry) uniform covariance with maximum correlation $0.8:V_{ij}=1000𝟙[i=j=1]+𝟙[i=j\neq 1]+0.8𝟙[i\neq j]$
- Diagonal identity (except first entry) banded covariance with maximum correlation $0.8:V_{ij}=1000𝟙[i=j=1]+𝟙[i=j\neq 1]+0.8^{\left|i-j\right|}𝟙[i\neq j]$

In our selection, we specifically included diagonal identity matrices (with the exception of the first entry) combined with either uniform or banded covariance structures, showcasing a maximum covariance of 0.8. This setting results in weaker correlation between the first component and the others. This choice was strategic to achieve higher condition numbers (around 5000 and 9000 respectively), given that correlations set at 0.8 or $0.8^{\left|i-j\right|}𝟙[i\neq j]$ yield condition numbers around 400 and 80, respectively.

**Figure 7:**
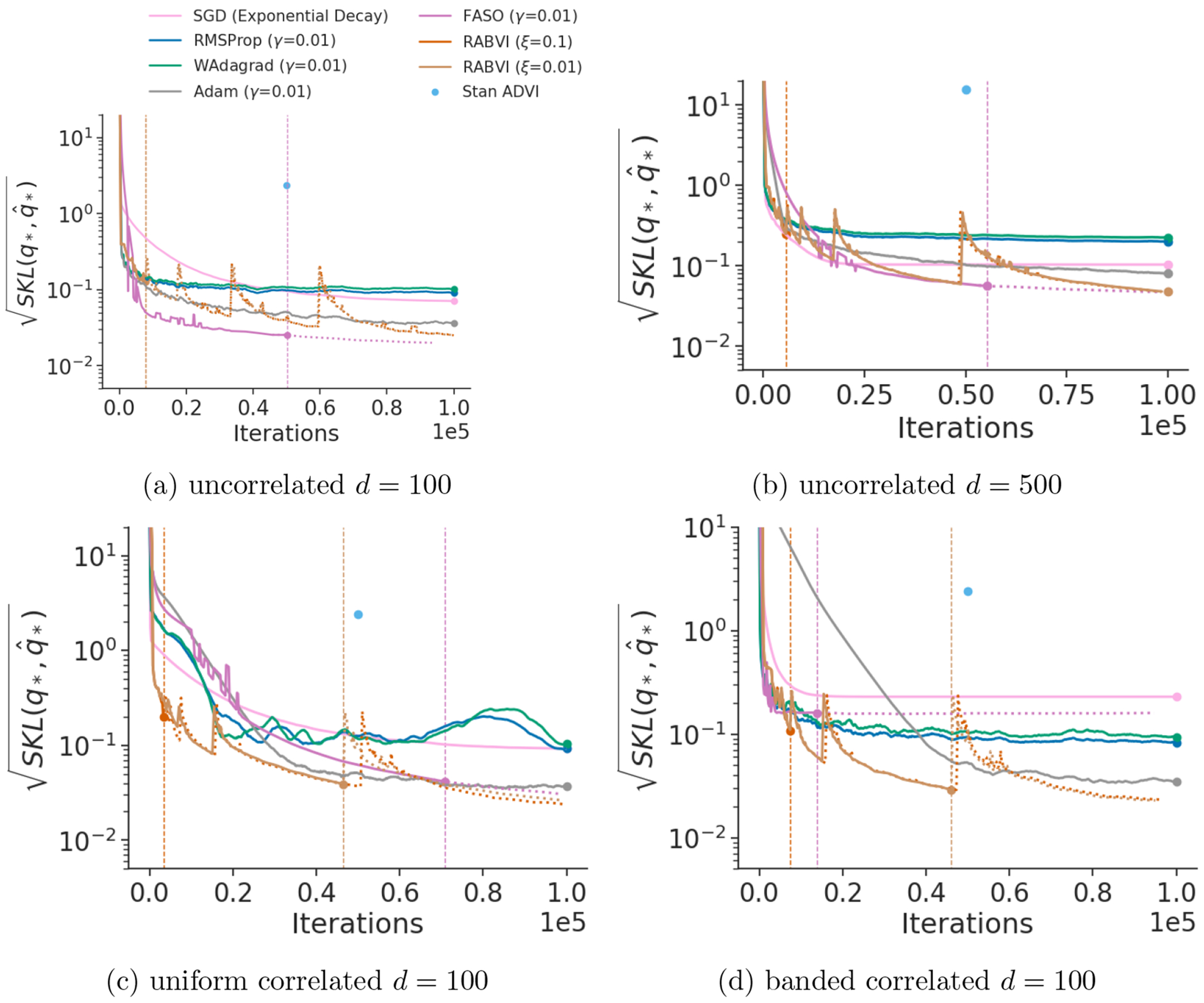
Accuracy comparison of variational inference algorithms using Gaussian targets, where accuracy is measured in terms of the square root of symmetrized KL divergence between iterate average and optimal variational approximation. The vertical lines indicate the termination rule trigger points of FASO and RABVI. Iterate averages for Adam, RMSProp, and WAdagrad computed at every 200th iteration using a window size of 20% of iterations.

Figures 7, C.8 and C.9 show the comparison of RABVI to FASO, Stan’s ADVI implementation, SGD with exponential decay learning rate (SGD-ED), Adam with exponential decay and cosine learning rates (Adam-ED, Adam-C), RMSProp with exponential decay and cosine learning rates (RMSProp-ED, RMSProp-C), and fixed-learning rate versions of RMSProp, Adam, Windowed Adagrad (WAdagrad). The findings demonstrate that RABVI consistently outperforms ADVI, SGD-ED, both adaptive learning rate versions of RMSProp, and the fixed-learning rate methods in a majority of the cases. While Adam and SGD-ED occasionally reach performance levels similar to RABVI, they tend to converge more slowly and with less reliability. Additionally, despite Adam-ED and Adam-C closely matching RABVI’s performance on most problems, they lack a dependable mechanism for determining when to terminate the optimization at a desired accuracy level. Although a validation data set can be used, this requires the availability of such a data set and would allow for control of the approximation accuracy. On the other hand, by varying the accuracy threshold ξ, the quality of the final RABVI approximation ${\overset{ˆ}{q}}_{*}$ also varies such that $SKL{\left(q_{*},{\overset{ˆ}{q}}_{*}\right)}^{1/2}\approx \xi$.

**Figure C.10:**
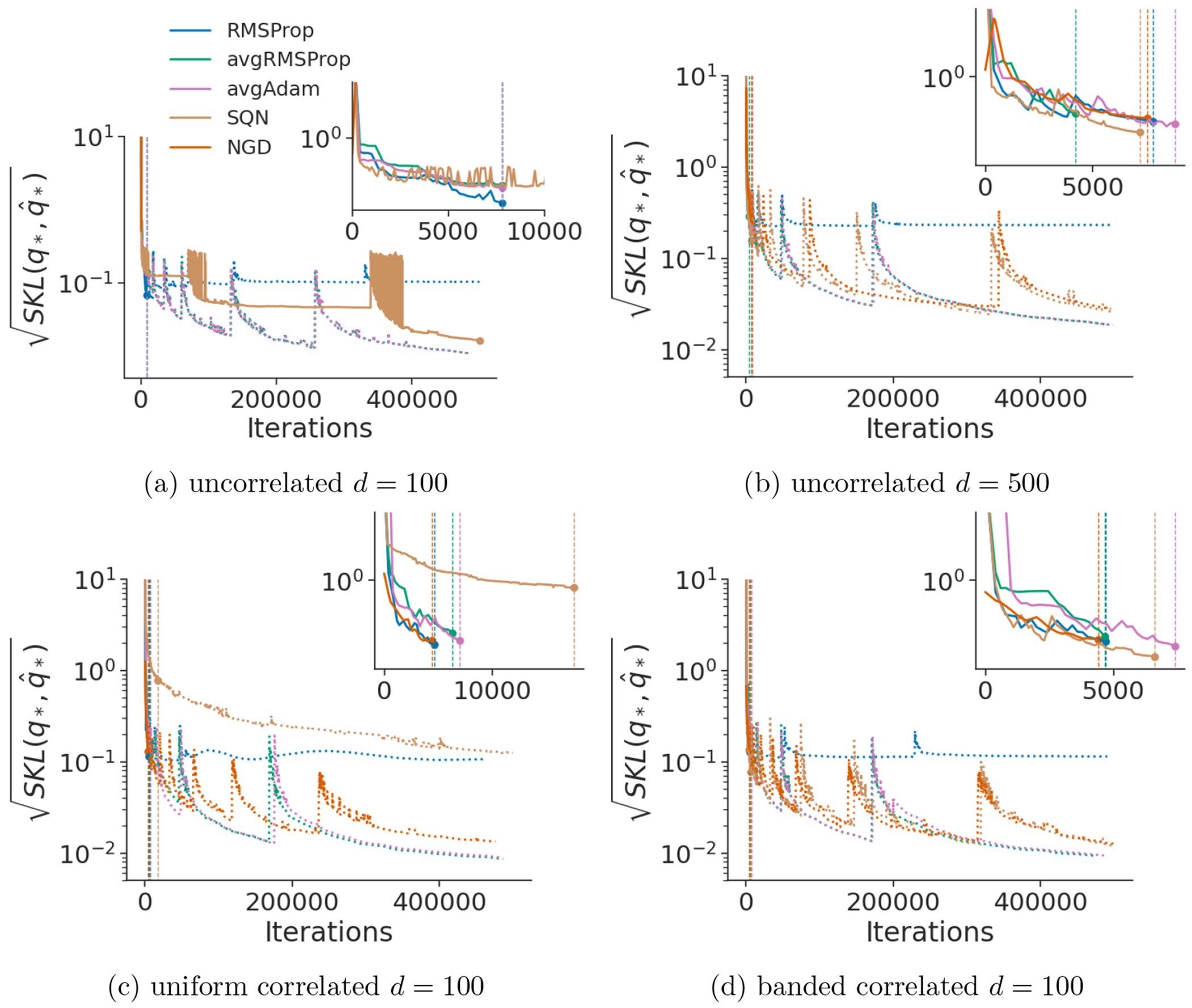
Accuracy comparison of *RMSProp, avgRMSProp, avgAdam, SQN*, and *NGD* optimization methods in RABVI using Gaussian targets where accuracy is measured in terms of square root of symmetrized KL divergence between iterate average and optimal variational approximation. The vertical lines indicate the termination rule trigger points and the behavior of optimization methods at the trigger points showed in inset plots.

To demonstrate the flexibility of our framework, we used RABVI with a variety of optimization methods: RMSProp, avgRMSProp, avgAdam, natural gradient descent (NGD), and stochastic quasi-Newton (SQN). See Appendices B.5 and B.6 for details. Figure C.10 shows that avgAdam and avgRMSProp optimization methods have a similar improvement in symmetrized KL divergence between optimal and estimated variational approximation for all cases. NGD is not stable for the diagonal non-identity covariance structure and SQN does not perform well with uniform covariance structure. Even though RMSProp shows an improvement in accuracy for large step sizes, accuracy does not improve as the step size decreases.

### 7.2 Reliability Across Applications

**Figure C.11:**
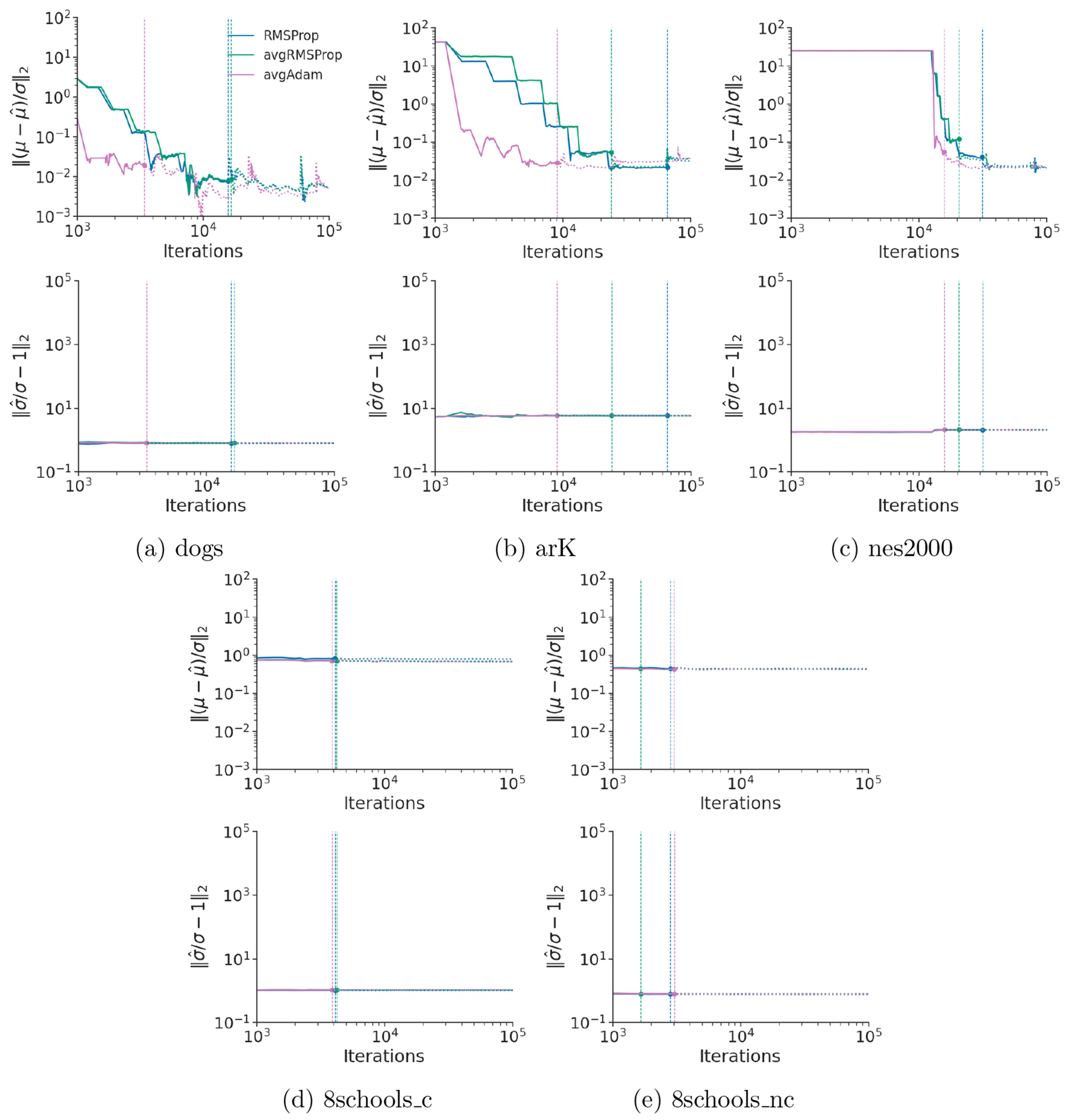
Accuracy comparison of *RMSProp, avgRMSProp*, and *avgAdam* optimization methods in RABVI using *posteriordb* datasets, where accuracy measured in terms of relative mean error (top) and relative standard deviation error (bottom). The vertical lines indicate the termination rule trigger points.

To validate the robustness and reliability of RABVI across realistic use cases, we consider 18 diverse data set/model pairs found in the posteriordb package^3^ (see Appendix C for details). The posteriordb package contains a wide range of real-world data and models and is specifically designed to provide realistic performance evaluations of approximate posterior inference algorithms. The accuracy was computed based on ground-truth estimates obtained using the posterior draws included in posteriordb package if available. Otherwise, we ran Stan’s dynamic HMC algorithm (Stan Development Team, 2020) to obtain the ground truth (4 chains for 50,000 iterations each). To stabilize the optimization, we initialize the variational parameter estimates using RMSProp for the initial learning rate only. A comparison across optimization methods validates our choice of avgAdam over alternatives (Fig. C.11).

#### Comparison to alternative optimization methods.

**Figure 8:**
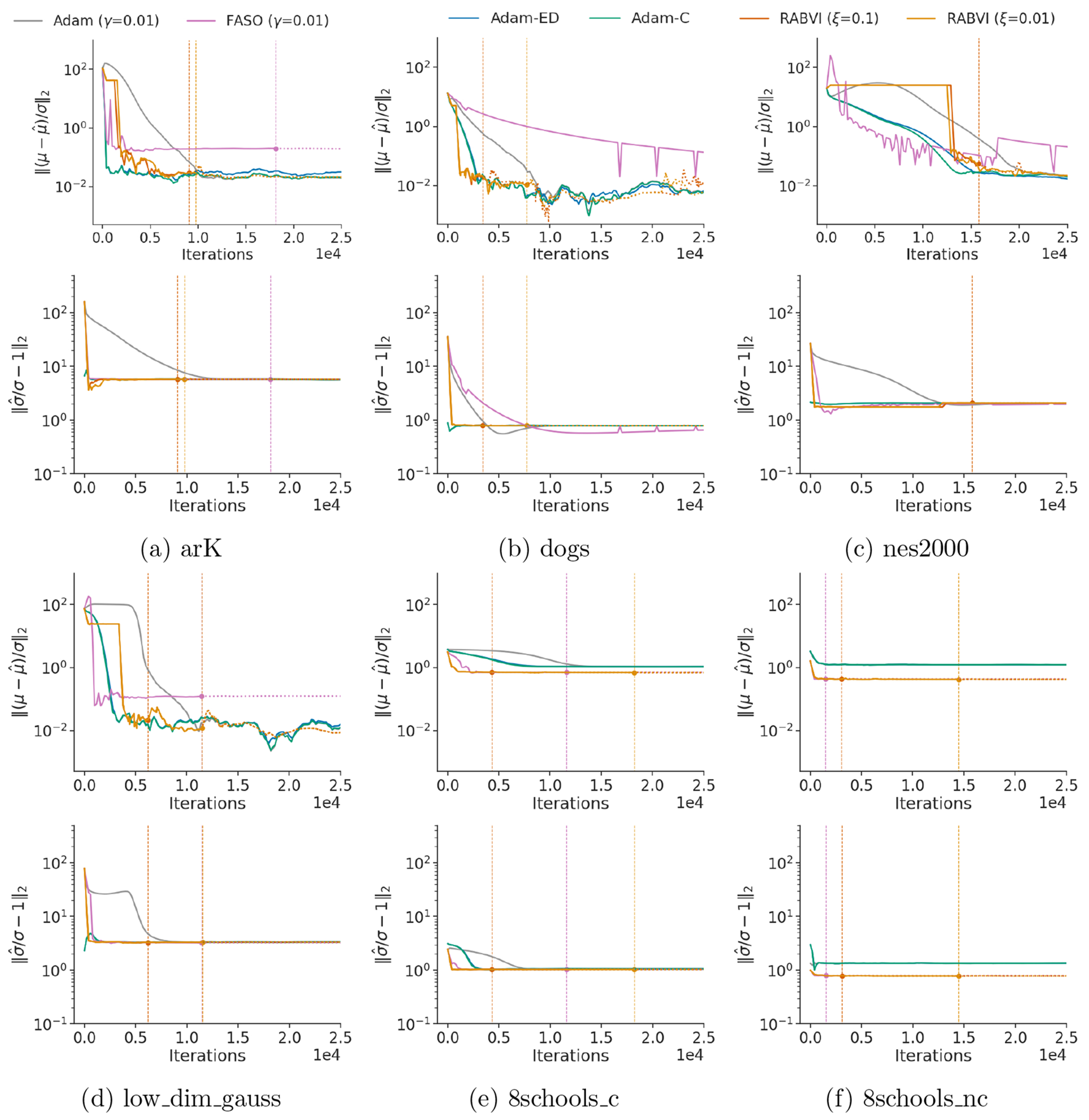
Accuracy comparison of variational inference algorithms using posteriordb models and data sets, where accuracy is measured in terms of relative mean error (top) and relative standard deviation error (bottom). The vertical lines indicate the termination rule trigger points of FASO and RABVI. The iterate average for Adam is computed at every 200^*th*^ iteration using a window size of 20% of iterations.

To evaluate RABVI’s effectiveness in real-world applications, we compared it against alternative optimization methods with both fixed and adaptive learning rate schedules. Based on the results described in Section 7.1, we opt to compare to Adam using either a fixed, exponential decay, or cosine learning rate schedule since they perform best overall in the Gaussian target experiments. Additionally, we include FASO, which used avgAdam, as another benchmark. Figure 8 shows RABVI is more consistent than all the alternative methods. While these methods sometimes matched RABVI’s performance, RABVI’s ability to identify an appropriate stopping point contributes to its overall efficiency, setting it apart from the competition.

#### Accuracy and robustness.

**Figure 9:**
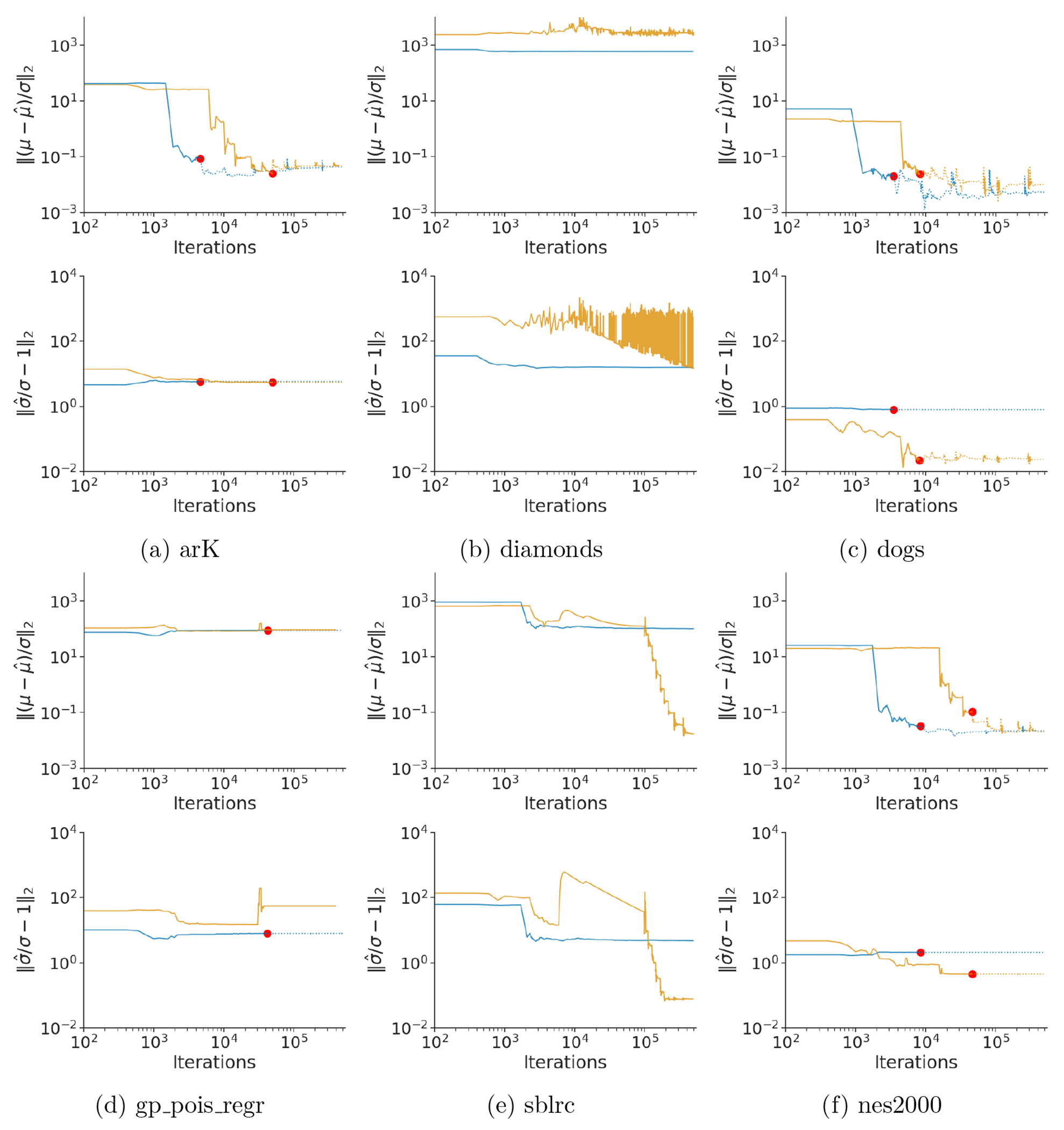
Accuracy of mean-field (blue) and full-rank (orange) Gaussian family approximations for selected posteriordb data/models, where accuracy is measured in terms of relative mean error (top) and relative standard deviation error (bottom). The red dots indicate where the termination rule triggers.

First, we investigate the accuracy and algorithmic robustness of RABVI. In terms of robustness, Figures 9, C.12 and C.13 validate our termination criteria since after reaching the termination point there is no considerable improvement in the accuracy for most of the models and data sets. While in many cases the mean estimates are quite accurate, the standard deviation estimates tended to be poor, which is consistent with typical behavior of mean-field approximations. To examine whether RABVI can achieve more accurate results with more flexible variational families, we conduct the same experiment using multivariate Gaussian approximation family and normalizing flow family using real-NVP flow with 2 hidden layers, 8 hidden units, and 3 coupling layers (Dinh et al., 2017). We employ FASO in our real-NVP experiments because the complexity of the approximation family prevents us from obtaining a closed form for the SKL divergence, which is necessary for computing the termination rule in RABVI. In some cases the accuracy of the mean and/or standard deviations estimates improve (*bball_0, dogs_log, 8schools_c, hudson_lynx, hmm_example, nes2000*, and *sblrc*). However, the results are inconsistent and sometimes worse due to the higher-dimensional, more challenging optimization problem. Our findings underscore the necessity of supplementing an improved optimization framework like RABVI with diagnostics for assessing the accuracy of the posterior approximation (Yao et al., 2018; Huggins et al., 2020; Wang et al., 2023c).

#### Comparison to MCMC.

**Figure 10:**
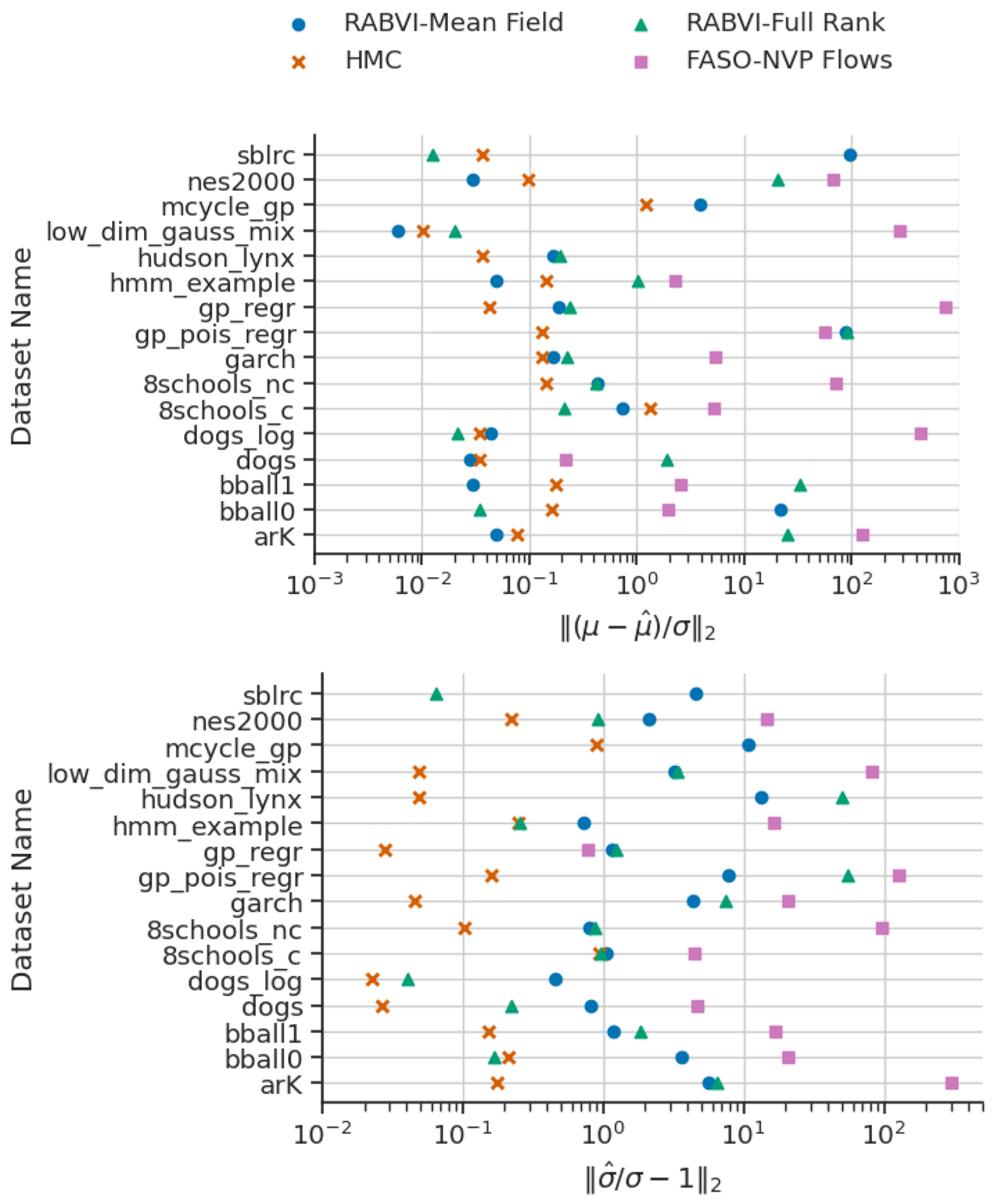
Results of RABVI with mean-field Gaussian and full-rank Gaussian family and FASO with real NVP flows comparison to dynamic HMC at the same computational cost (likelihood evaluations) in terms of relative mean error (top) and relative standard deviation error (bottom).

**Figure C.17:**
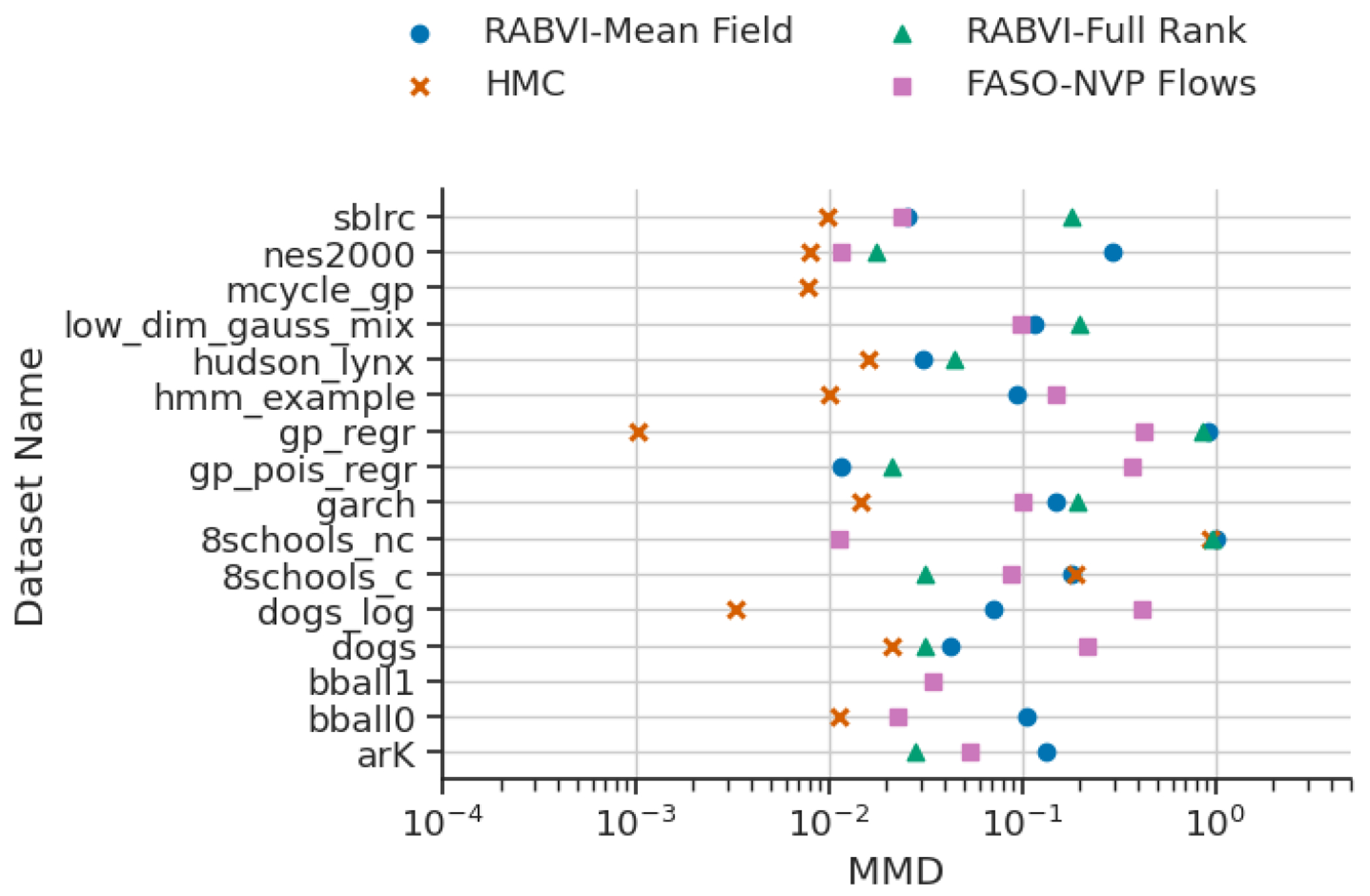
Results of RABVI with mean-field Gaussian and full-rank Gaussian family and FASO with NVP flows comparison to dynamic HMC at the same computational cost (likelihood evaluations) in terms of MMD.

We additionally benchmarked the runtime and accuracy of RABVI to Stan’s dynamic HMC algorithm, for which we ran 1 chain for 25,000 iterations including 5,000 warmup iterations. We measure runtime in terms of the number of likelihood evaluations and compared the relative error between the methods at the RABVI termination rule trigger point or final likelihood evaluation of HMC (whichever comes first). Figures 10, C.15 and C.16 show that RABVI tends to provide similar or better posterior mean estimates (the exceptions are *gp_pois_regr, hudson_lynx*, and *sblrc*). However, the RABVI standard deviation estimates tend to be less accurate even when using the full-rank Gaussian variational family. This could be because the optimization of full-rank Gaussian is more challenging having more variational parameters to estimate (Bhatia et al., 2022). Figure C.17 shows that, in terms of the MMD, the HMC approximation is closer to the target than BBVI as one would expect. Overall, the MMD values for RABVI are reasonably small.

**Figure C.18:**
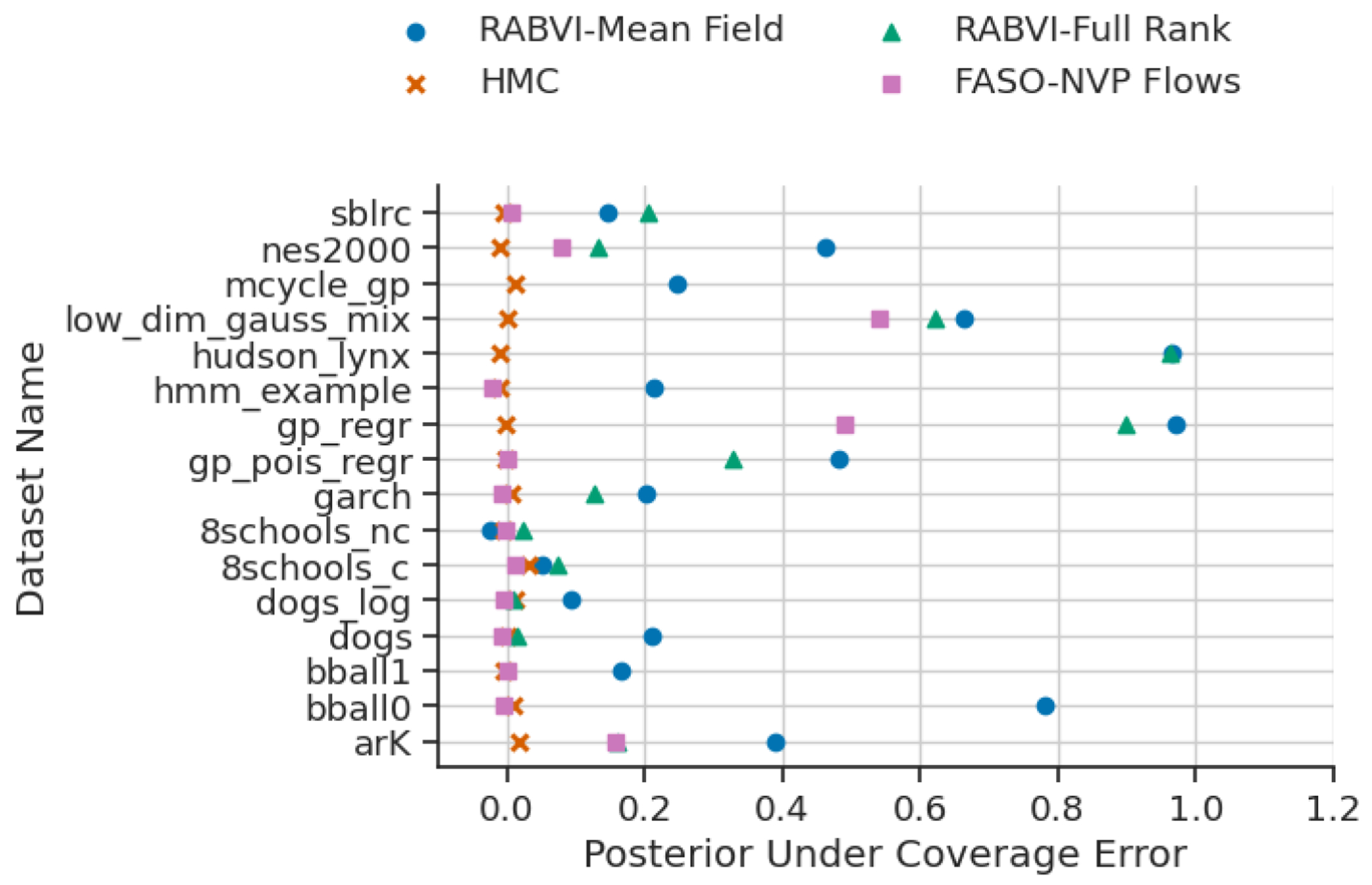
Results of RABVI with mean-field Gaussian and full-rank Gaussian family and FASO with NVP flows comparison to dynamic HMC at the same computational cost (likelihood evaluations) in terms of posterior under coverage error of 95% quantiles.

We also compared the 95% quantiles posterior under coverage error between RABVI and FASO methods using different approximation families and MCMC. Figure C.18 shows that HMC and real-NVP flows do not undercover the posterior. However, the mean-field and full-rank Gaussian families do.

### 7.3 Robustness to Tuning Parameters: Ablation Study

To validate the robustness of RABVI to different choices of algorithm tuning parameters, we consider the Gaussian targets and two *posteriordb data set/model pairs: dogs (logistic mixed-effects model) and arK (AR(5) time-series model)*. We vary one tuning parameter while keeping the recommended default values for all others. We consider the following values for each parameter (default in bold):

- initial learning rate $\gamma_{0}:0.01,0.1,0.3,0.5$
- minimum window size $W_{\text{min}}:100,200,300,500$
- initial iterate average relative error threshold $\varepsilon_{0}:0.05,0.1,0.2,0.5$
- inefficiency threshold τ:0.1,0.5,1.0,1.2
- Monte Carlo samples M:1,5,10,15,25.

**Figure 11:**
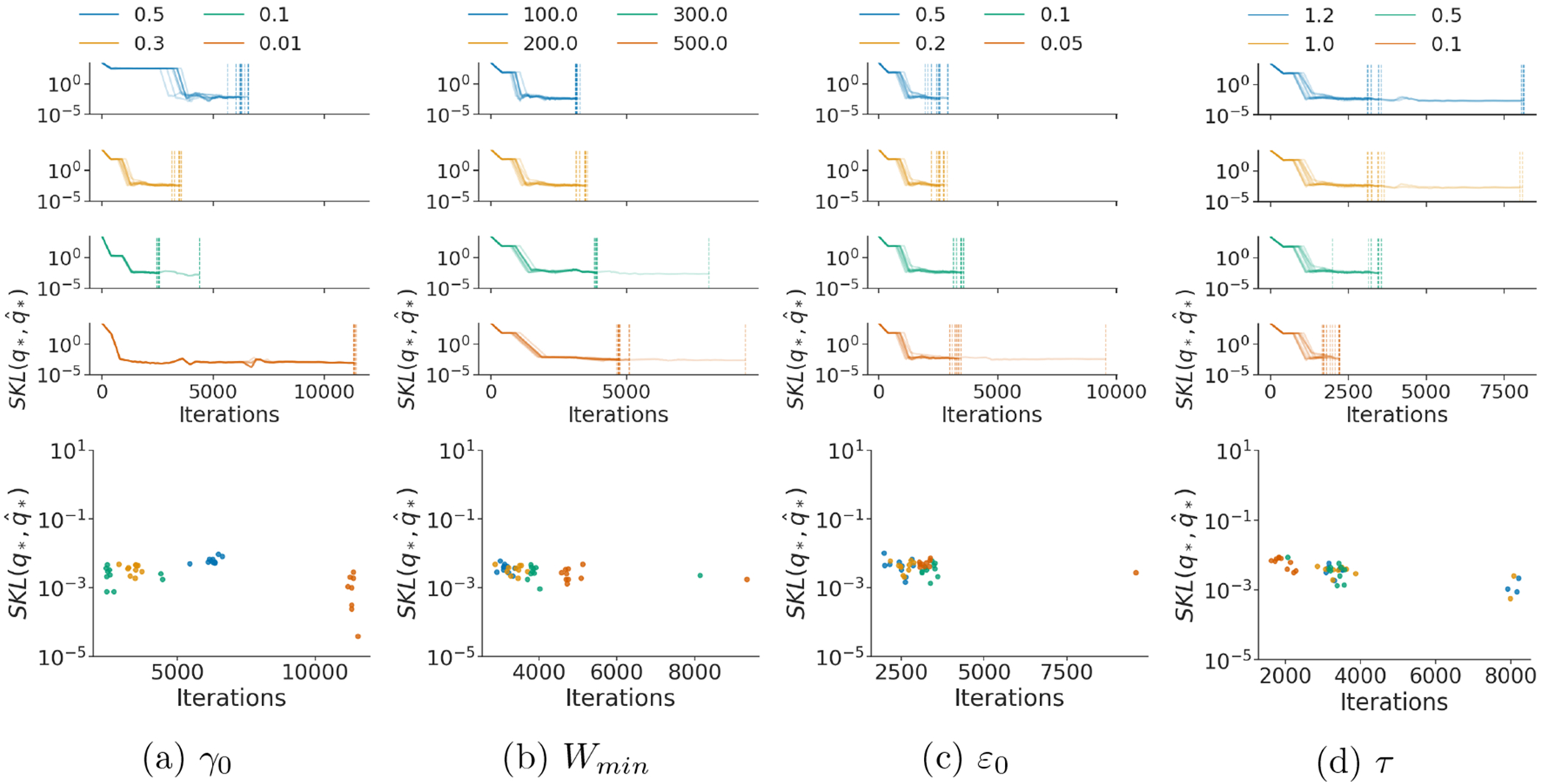
Robustness to tuning parameters (a) initial learning rate *γ*_0_, (b) minimum window size *W*_min_, (c) initial iterate average relative error threshold *ε*_0_, and (d) inefficiency threshold *τ* using *dogs* data set from posteriordb package. **(top)** Iterations versus symmetrized KL divergence between iterate average and optimal variational approximation. The transparent lines represent repeated experiments and the vertical lines indicate the termination rule trigger points. **(bottom)** Iterations versus symmetrized KL divergence between iterate average and optimal variational approximation at the termination rule trigger point.

We repeat each experimental condition 10 times to verify the robustness of different initializations of the variational parameters. Figures 11 and C.19 to C.25 suggest that overall the accuracy and runtime of RABVI is not too sensitive to the choice of the tuning parameters. However, extreme tuning parameter choices (for example, γ=0.01 or M≤5) can lead to longer runtimes.

## 8. Discussion

As we have shown through both theory and experiments, RABVI, our stochastic optimization framework for black-box variational inference, delivers a number of benefits compared to existing approaches:

- The user only needs to, at most, adjust a small number of tuning parameters which empowers the user to intuitively control and trade off computational cost and accuracy. Moreover, RABVI is robust, both in terms of accuracy and computational cost, to small changes in all tuning parameters.
- Our framework can easily incorporate different stochastic optimization methods such as adaptive versions, natural gradient descent, and stochastic quasi-Newton methods. In practice, we found that the averaged versions of RMSProp and Adam we propose perform particularly well. However the performance of RABVI will benefit from future innovations in stochastic optimization methodology.
- RABVI allows for any choice of tractable variational family and stochastic gradient estimator. For example, in many cases we find accuracy improves when using the full-rank Gaussian variational family rather than the mean-field one.

Our empirical results also highlight some of the limitations of BBVI, which sometimes is less accurate than dynamic HMC when given equal computational budgets. However, BBVI can be further sped up using, for example, data subsampling when the data set size is large (which was not the case for the posteriodb data sets from our experiments).

## Figures and Tables

**Figure C.4:**
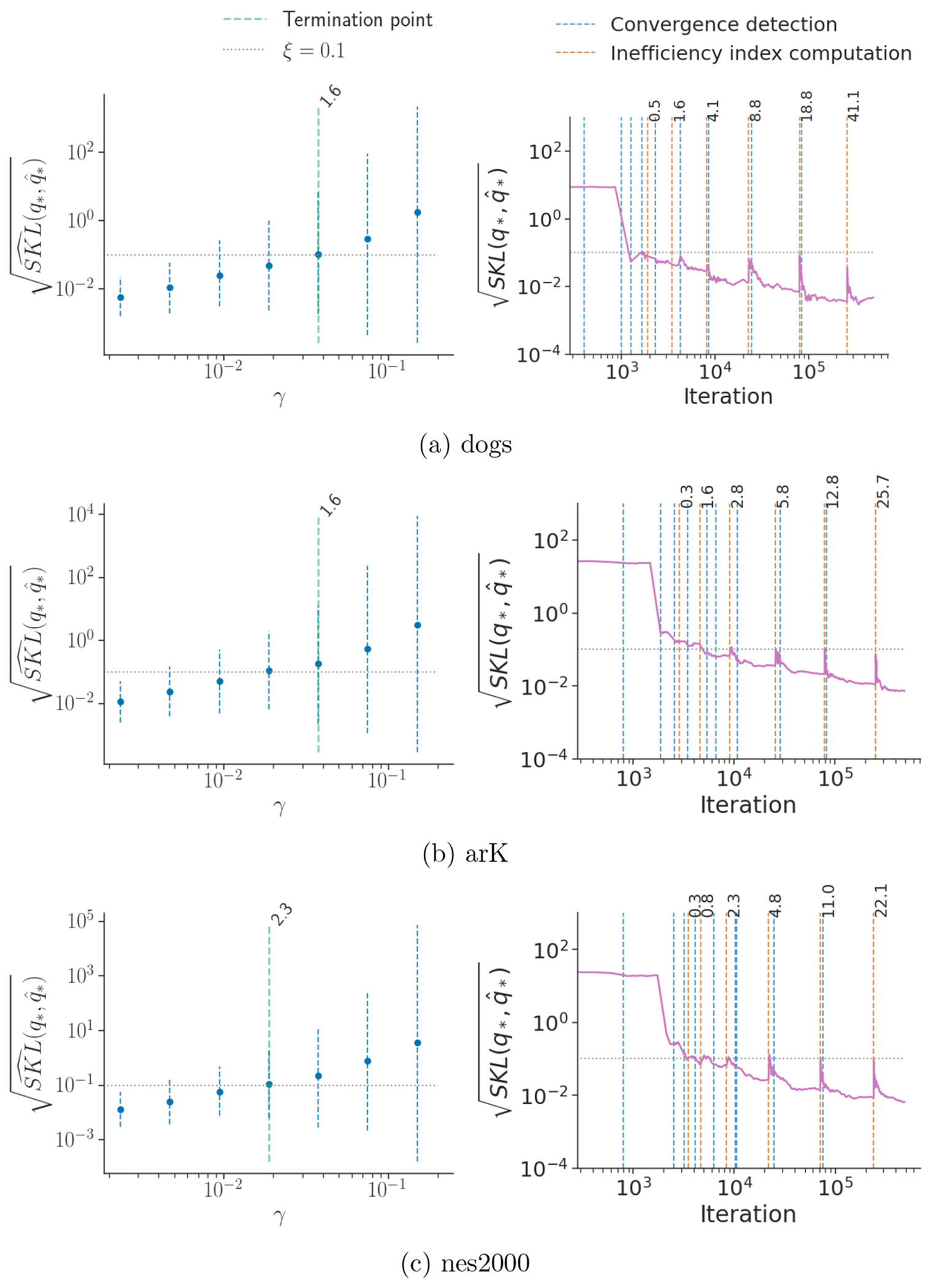
Results of termination rule trigger point of posteriordb package datasets/models. **(left)** Learning rate versus square root of estimated symmetrized KL divergence with 95% credible interval (dashed blue line). The green vertical line indicates the termination rule trigger point with corresponding $\overset{ˆ}{ℐ}$ value. **(right)** Iterations versus square root of symmetrized KL divergence between iterate average and optimal variational approximation. The vertical lines indicate the convergence detection points using $\overset{ˆ}{R}$ (blue) and inefficiency index computation ($\overset{ˆ}{ℐ}$) points (orange) with corresponding values.

**Figure C.5:**
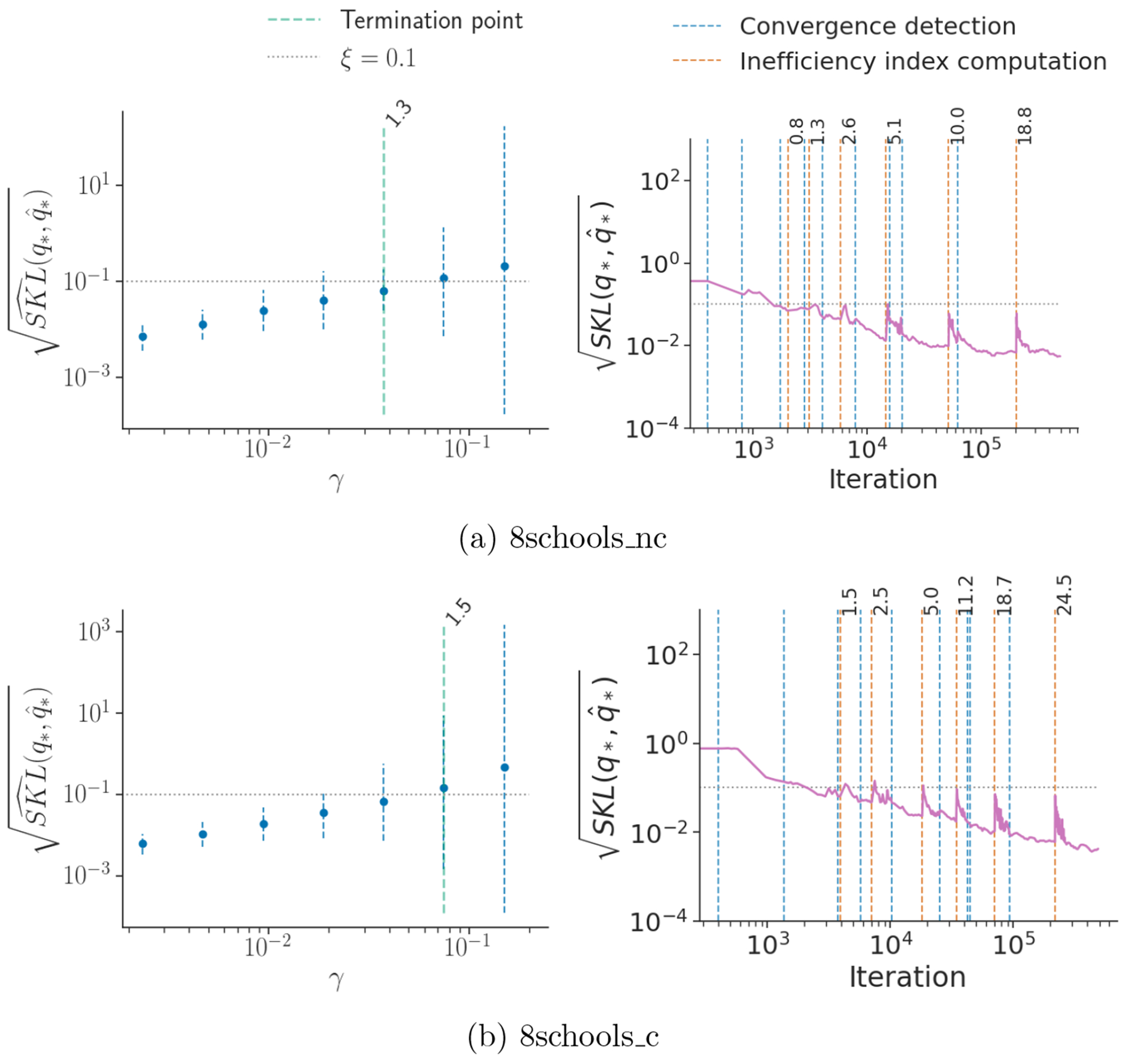
Results of termination rule trigger point of posteriordb package datasets/models. **(left)** Learning rate versus square root of estimated symmetrized KL divergence with 95% credible interval (dashed blue line). The green vertical line indicates the termination rule trigger point with corresponding $\overset{ˆ}{ℐ}$ value. **(right)** Iterations versus square root of symmetrized KL divergence between iterate average and optimal variational approximation. The vertical lines indicate the convergence detection points using $\overset{ˆ}{R}$ (blue) and inefficiency index computation ($\overset{ˆ}{ℐ}$) points (orange) with corresponding values.

**Figure C.8:**
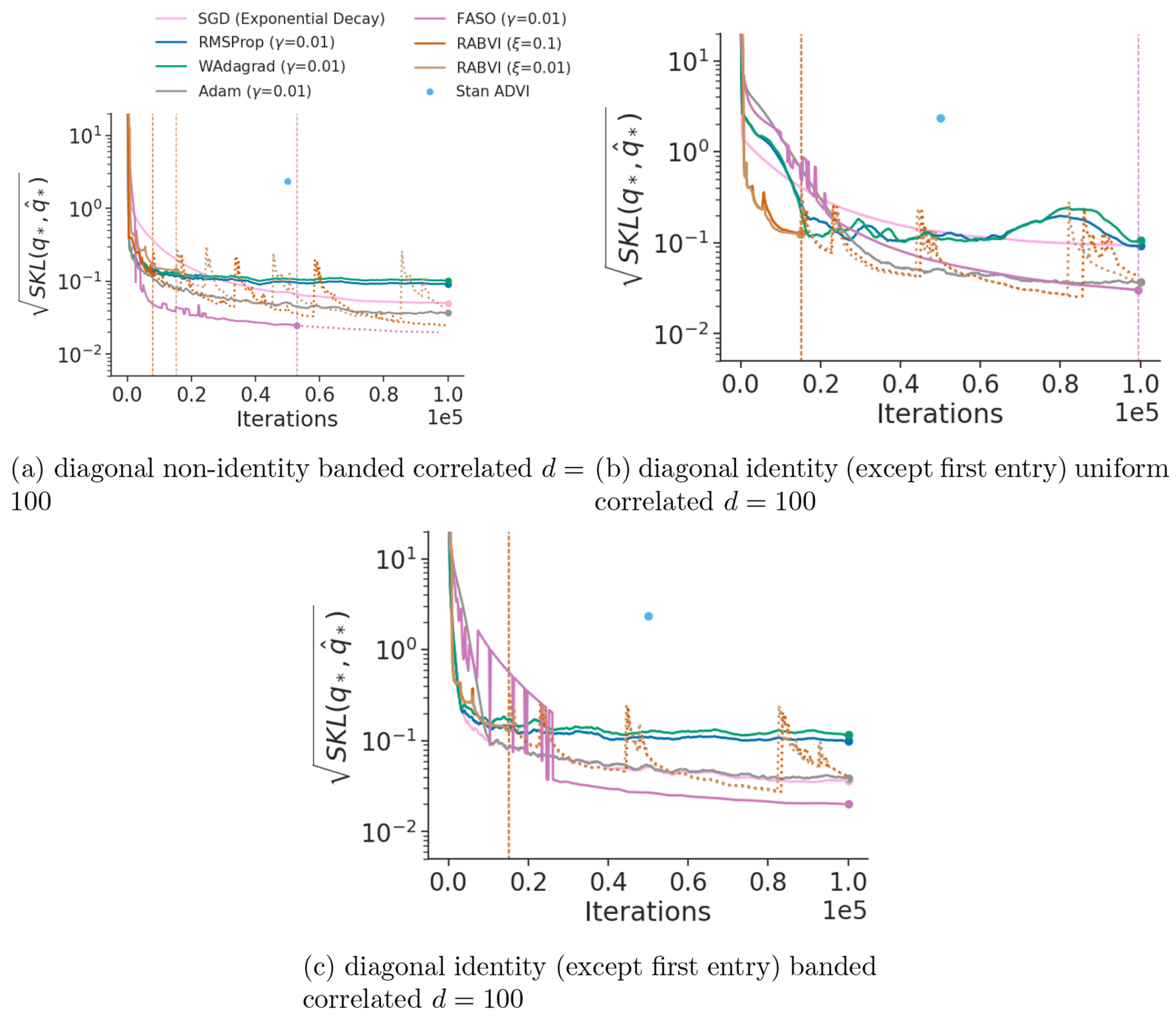
Accuracy comparison of variational inference algorithms using Gaussian targets, where accuracy is measured in terms of the square root of symmetrized KL divergence between iterate average and optimal variational approximation. The vertical lines indicate the termination rule trigger points of FASO and RABVI. Iterate averages for Adam, RMSProp, and WAdagrad computed at every 200th iteration using a window size of 20% of iterations.

**Figure C.9:**
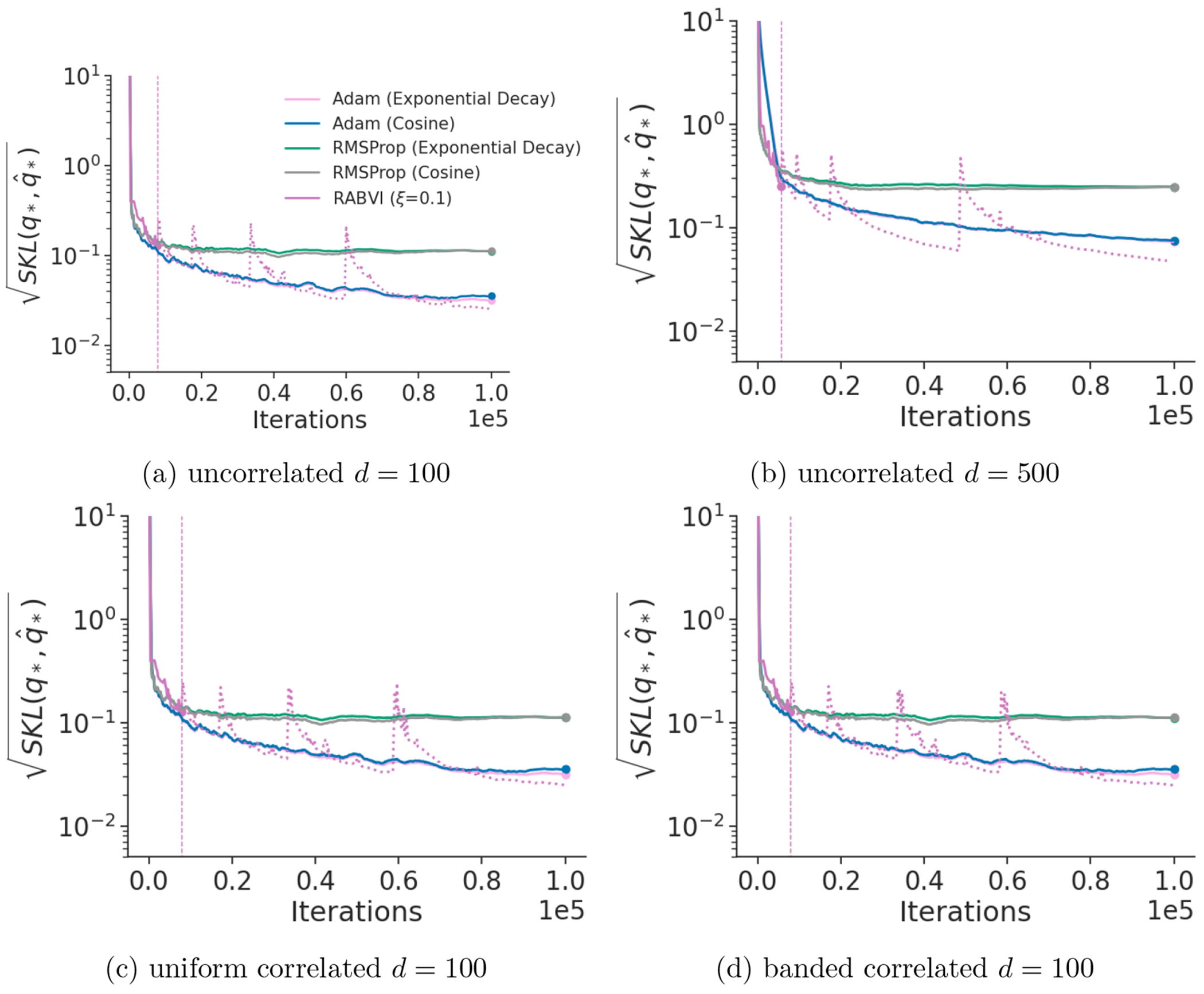
Accuracy comparison across learning rate schedules with Gaussian targets where accuracy is measured in terms of the square root of symmetrized KL divergence between iterate average and optimal variational approximation. The vertical lines indicate the termination rule trigger points of RABVI.

**Figure C.12:**
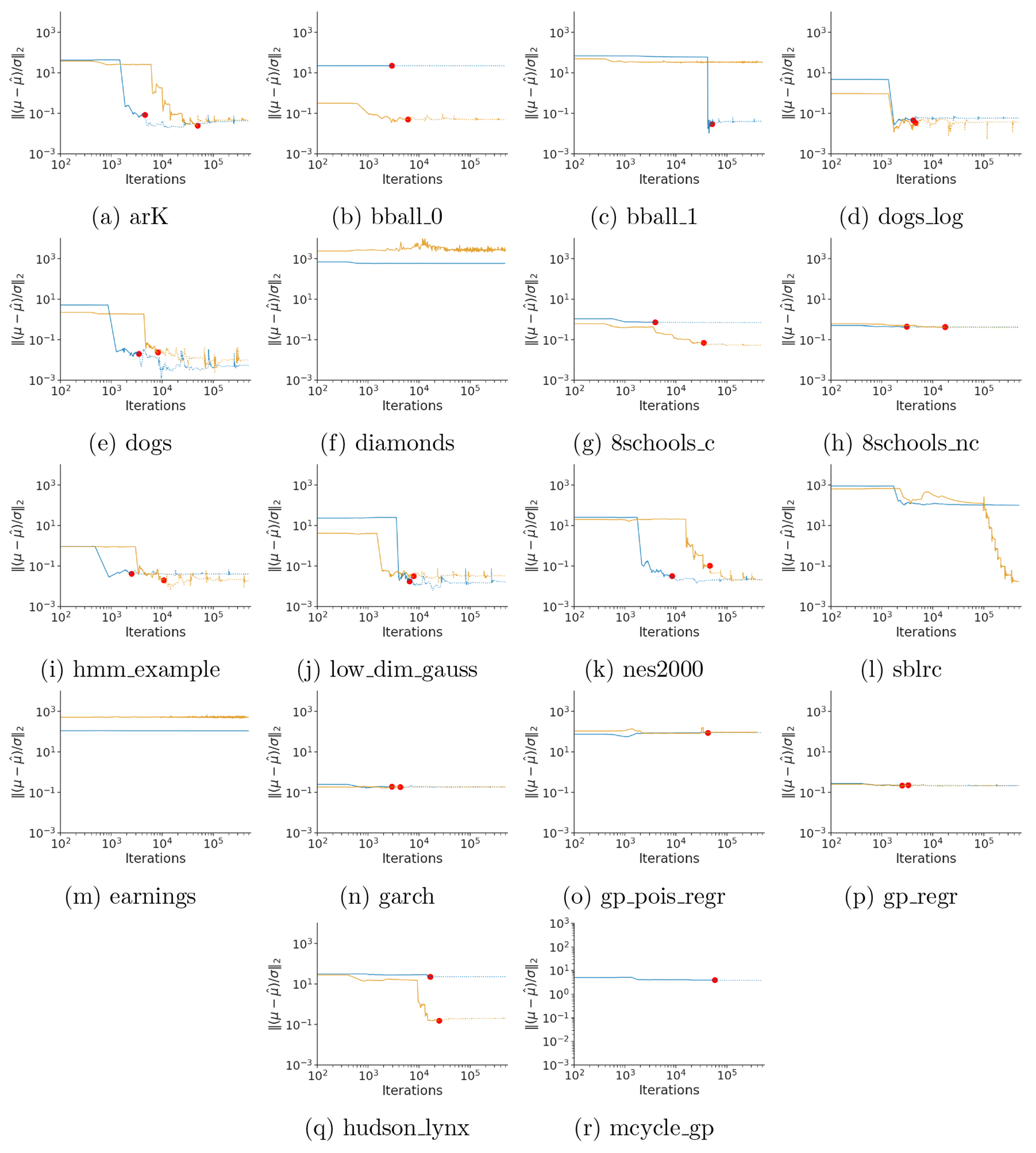
Accuracy of mean-field (blue) and full-rank (orange) Gaussian family approximations for selected posteriordb data/models, where accuracy is measured in terms of relative mean error. The red dots indicate where the termination rule triggers

**Figure C.13:**
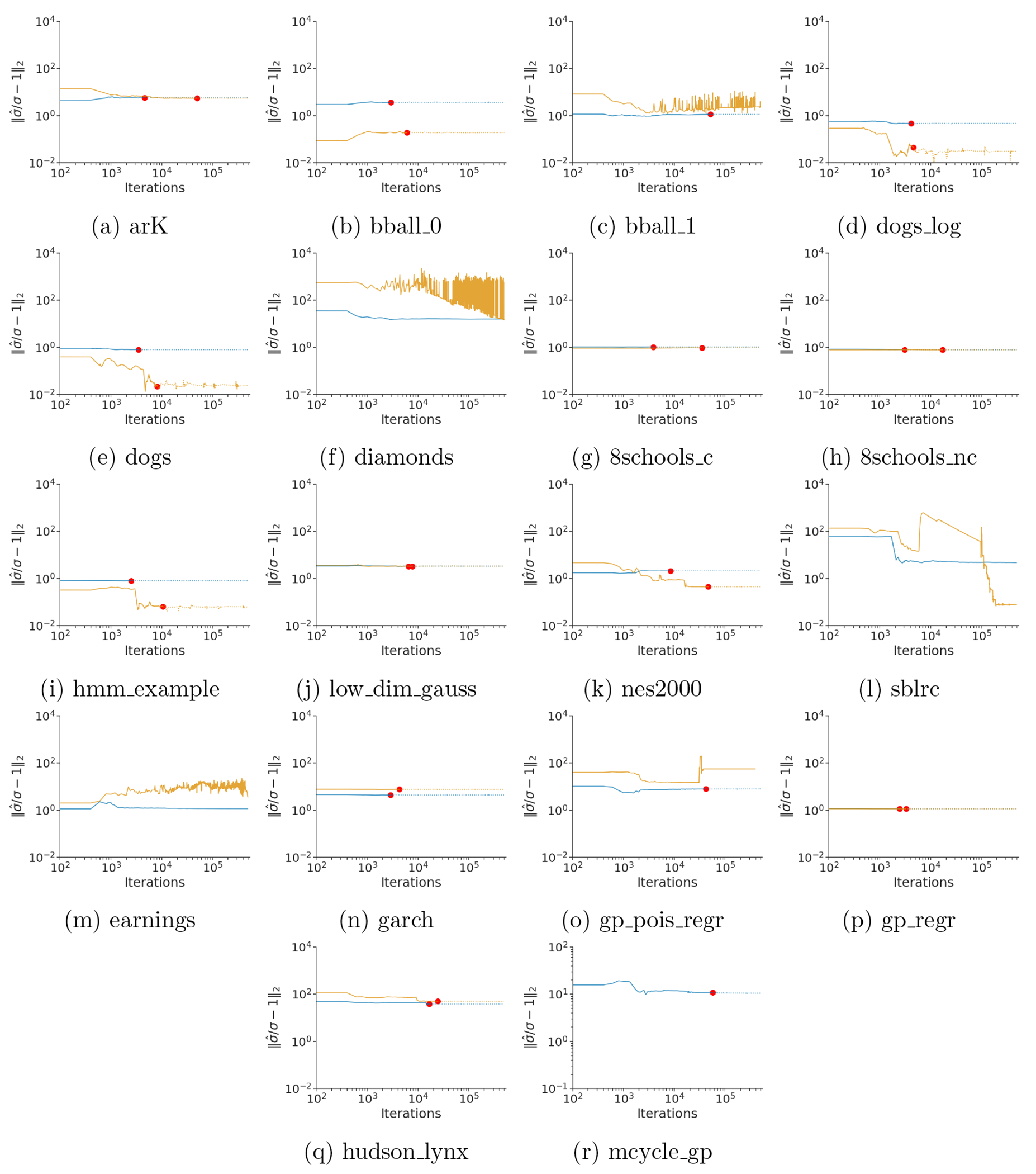
Accuracy of mean-field (blue) and full-rank (orange) Gaussian family approximations for selected posteriordb data/models, where accuracy is measured in terms of relative standard deviation error. The red dots indicate where the termination rule triggers

**Figure C.14:**
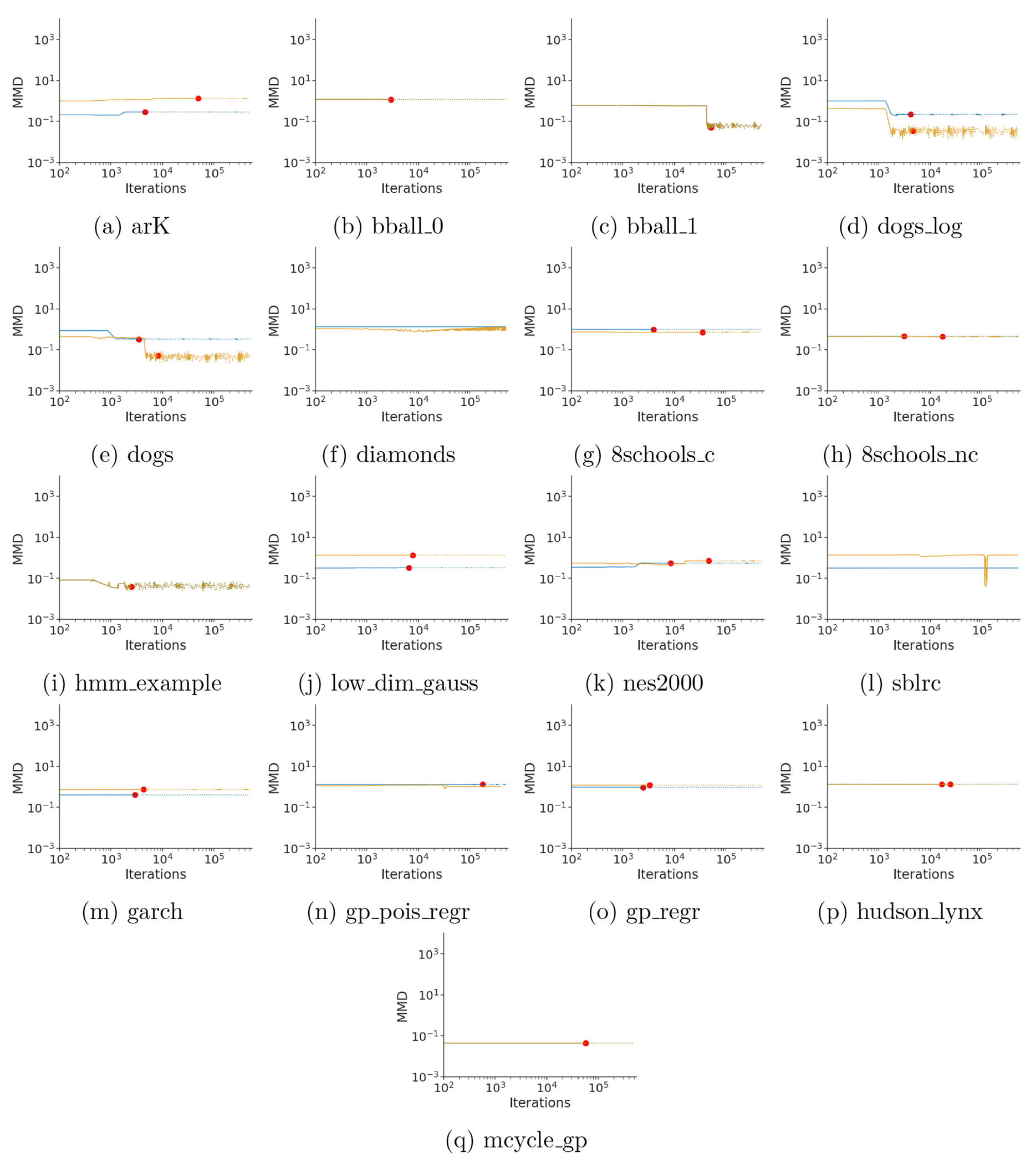
Accuracy of mean-field (blue) and full-rank (orange) Gaussian family approximations for selected posteriordb data/models, where accuracy is measured in MMD. The red dots indicate where the termination rule triggers

**Figure C.15:**
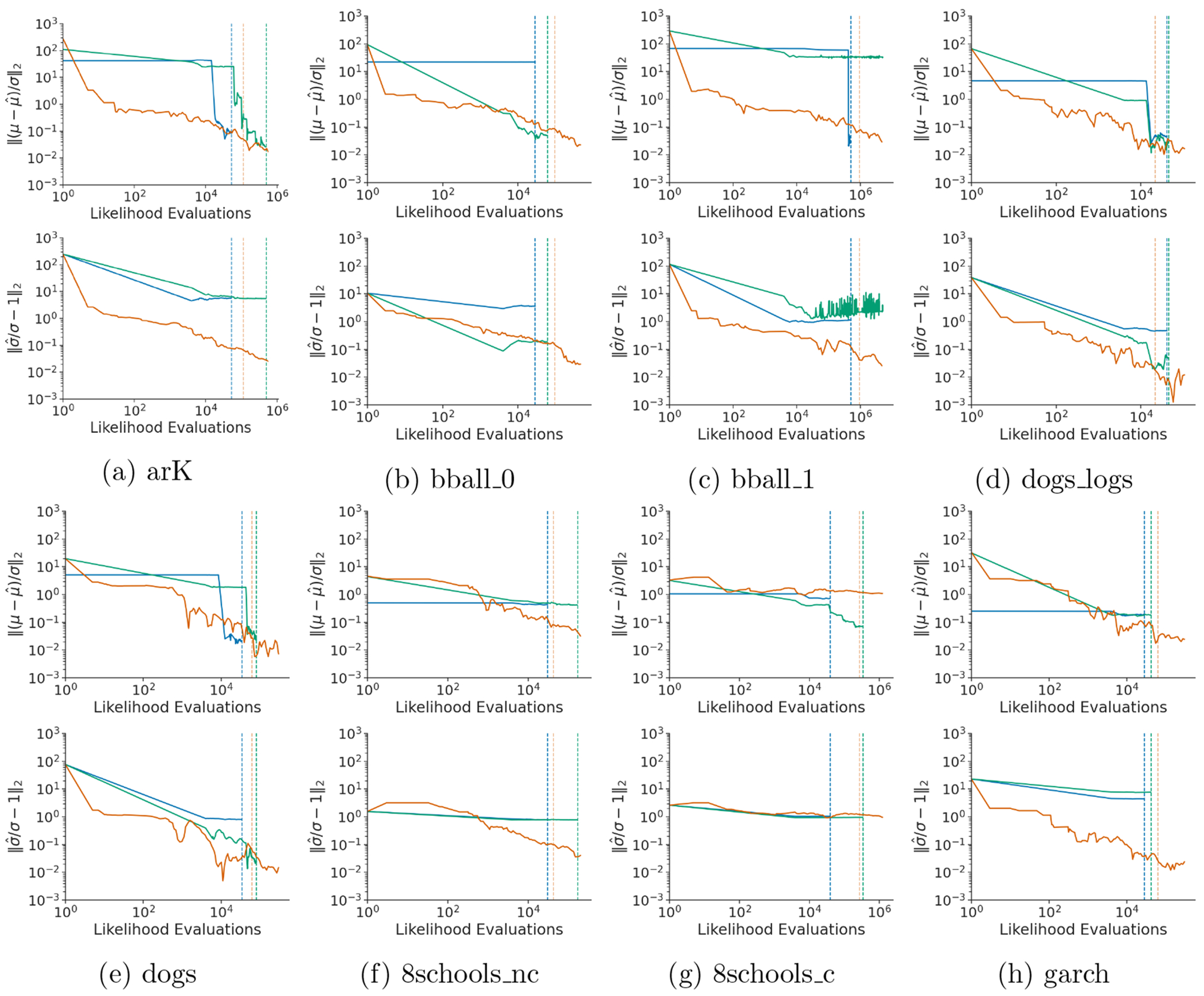
Results of RABVI with mean-field Gaussian (blue) and full-rank Gaussian (green) family comparison to dynamic HMC (orange) in terms of relative mean error (top) and relative standard error (bottom). Blue and green vertical lines show the termination rule triggering points in RABVI and orange vertical line shows the end of warm-up period in dynamic HMC.

**Figure C.16:**
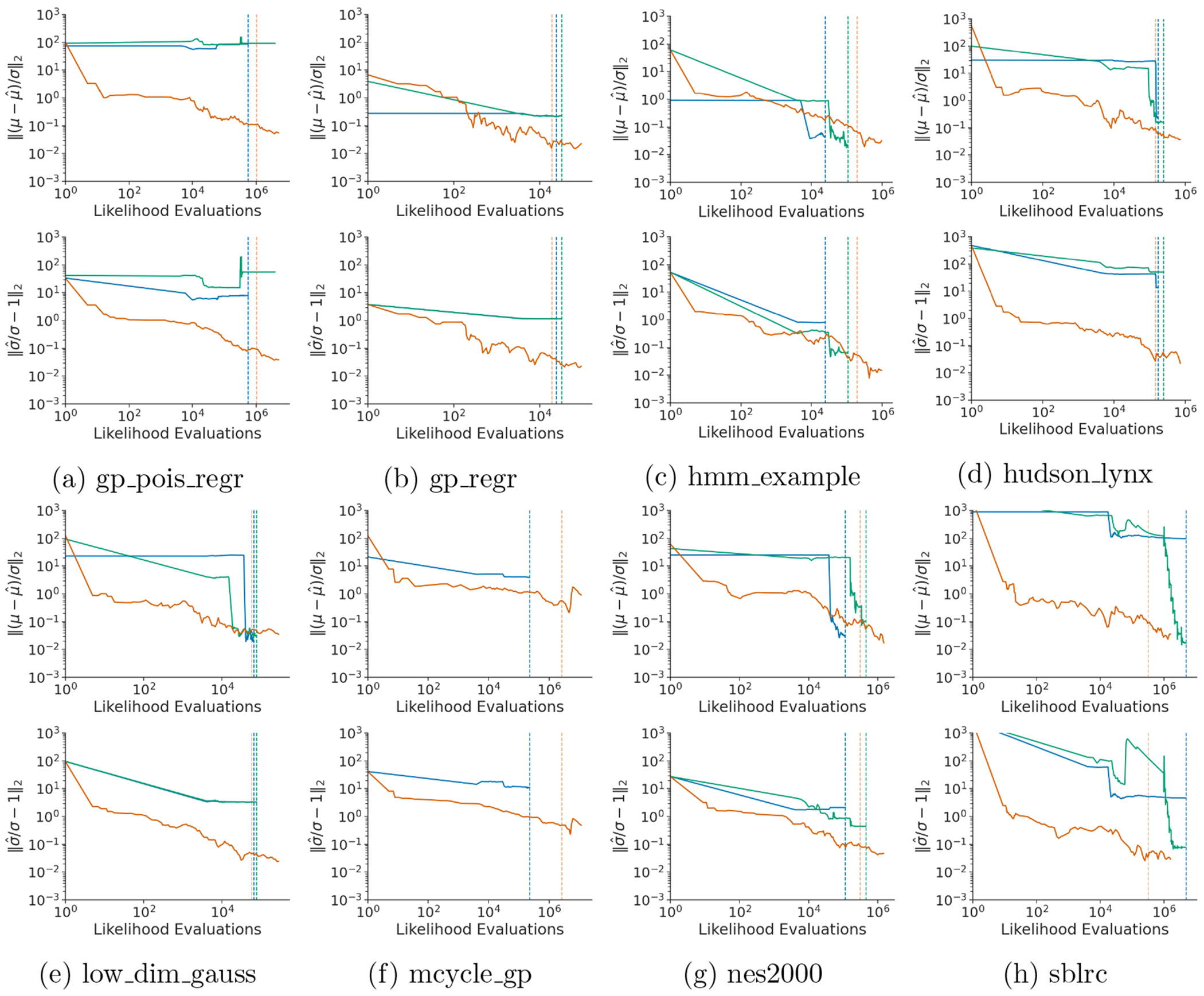
Results of RABVI with mean-field Gaussian (blue) and full-rank Gaussian (green) family comparison to dynamic HMC (orange) in terms of relative mean error (top) and relative standard error (bottom). Blue and green vertical lines show the termination rule triggering points in RABVI and orange vertical line shows the end of warm-up period in dynamic HMC.

**Figure C.19:**
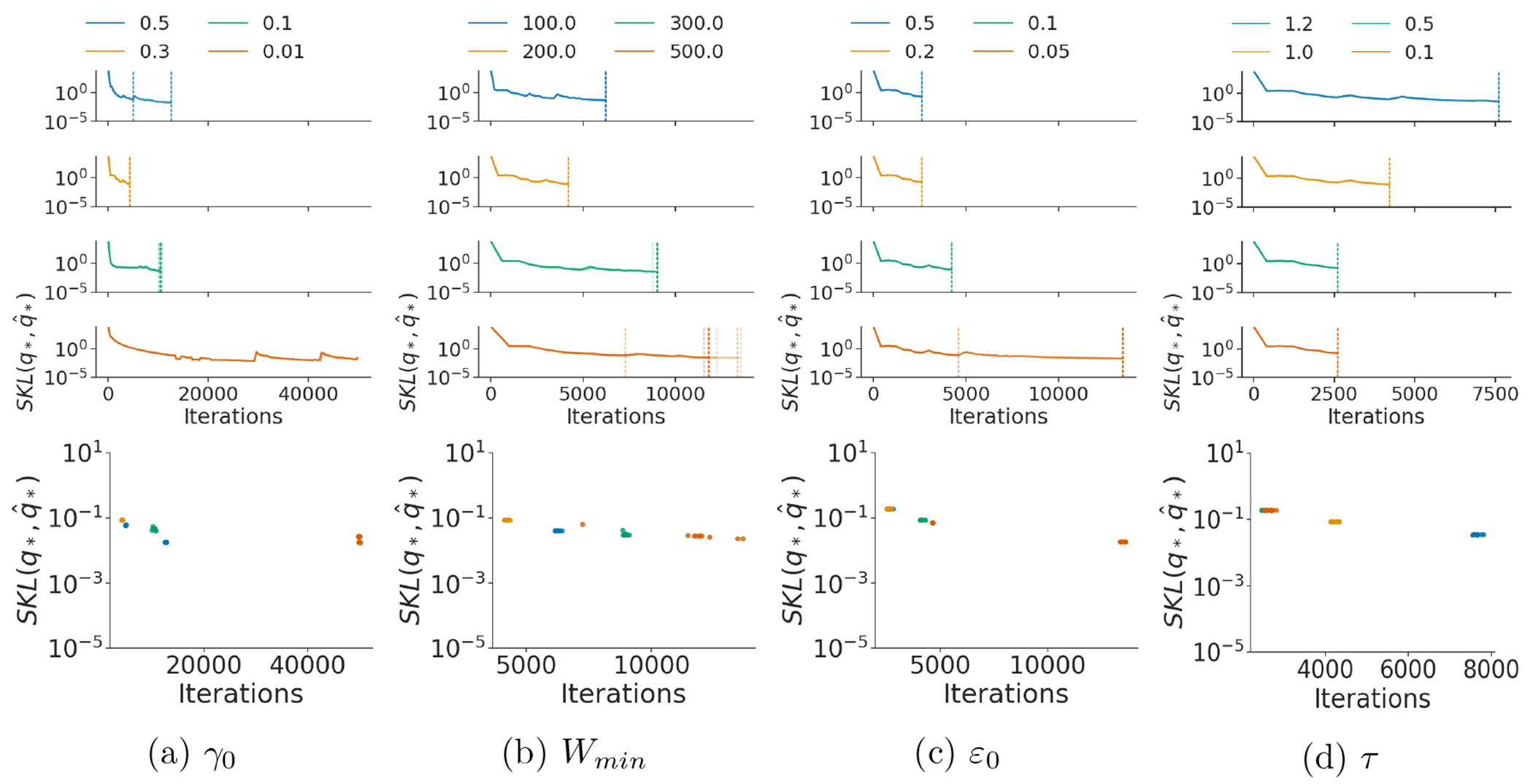
Robustness to tuning parameters **(a)** initial learning rate $\gamma_{0}$, **(b)** minimum window size $W_{\text{min}}$, **(c)** initial iterate average relative error threshold $\varepsilon_{0}$, and **(d)** inefficiency threshold τ. Results use Gaussian target 𝒩(0,V) with d=100 and V=I (identity covariance). **(top)** Iterations versus symmetrized KL divergence between iterate average and optimal variational approximation. The distinct lines represent repeated experiments and the vertical lines indicate the termination rule trigger points. **(bottom)** Iterations versus symmetrized KL divergence between iterate average and optimal variational approximation at the termination rule trigger point.

**Figure C.20:**
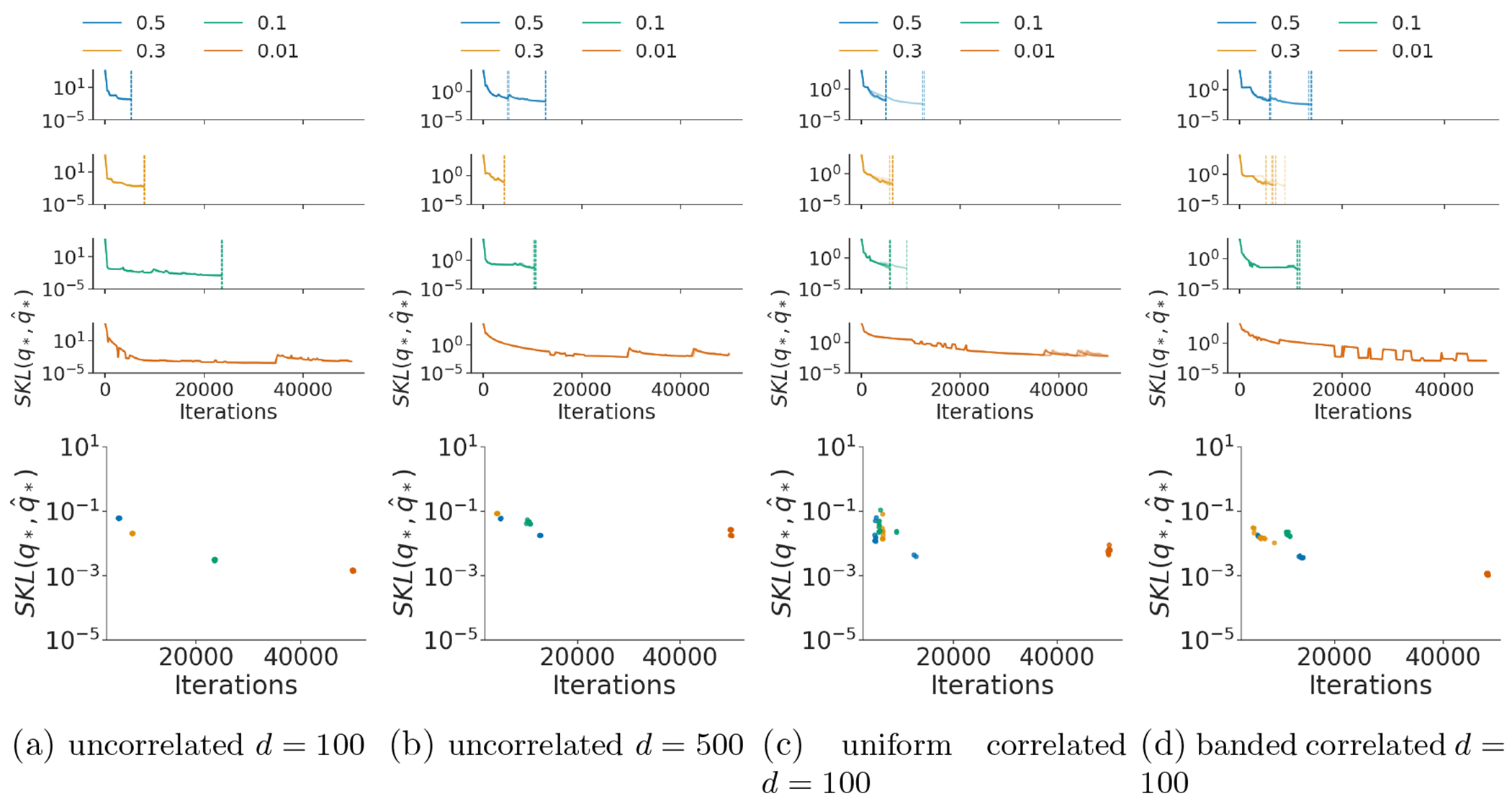
Robustness to initial learning rate $\gamma_{0}$ using Gaussian targets. **(top)** Iterations versus symmetrized KL divergence between iterate average and optimal variational approximation. The distinct lines represent repeated experiments and the vertical lines indicate the termination rule trigger points. **(bottom)** Iterations versus symmetrized KL divergence between iterate average and optimal variational approximation at the termination rule trigger point.

**Figure C.21:**
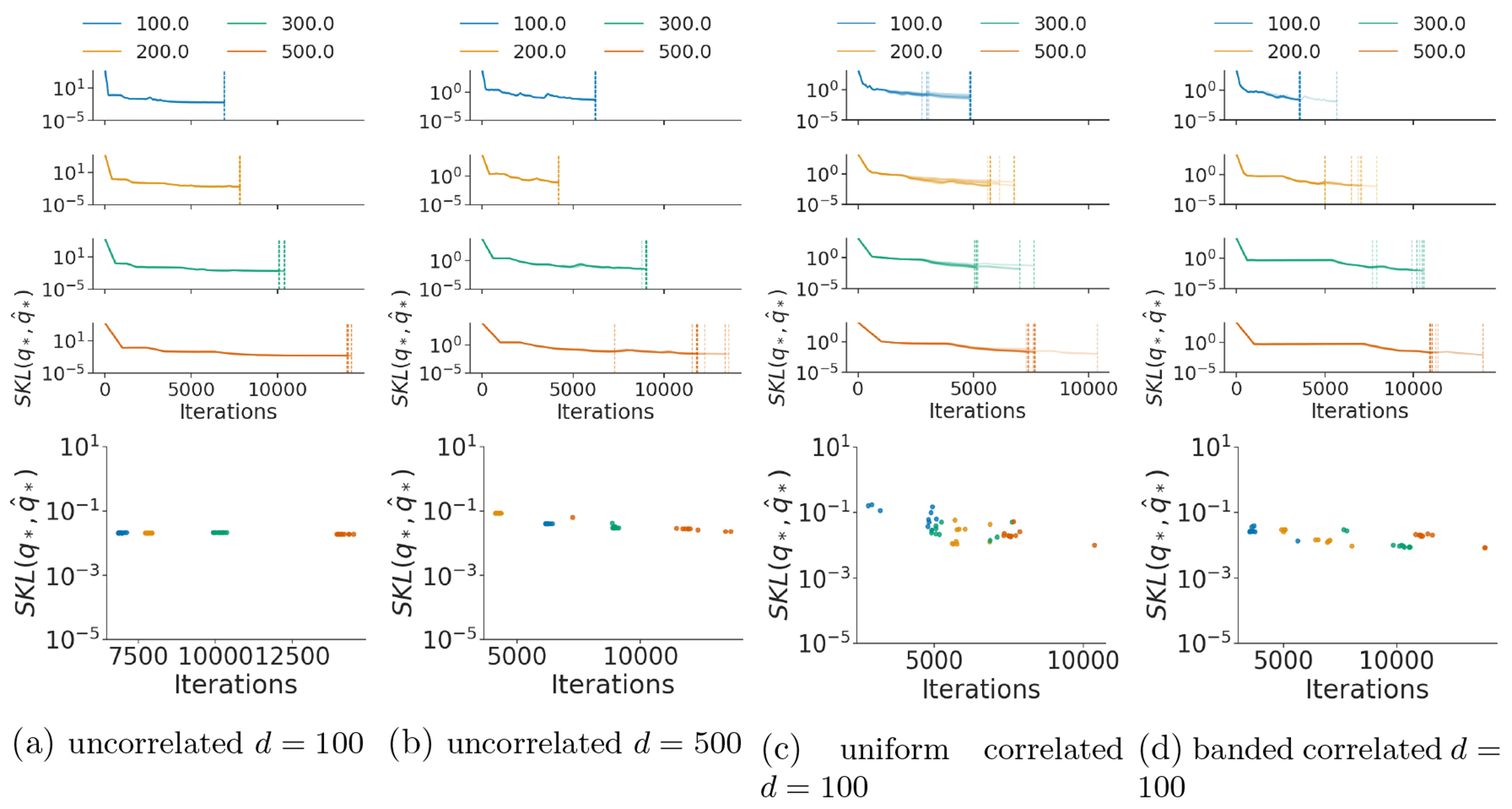
Robustness to minimum window size $W_{\text{min}}$,, using Gaussian targets. **(top)** Iterations versus symmetrized KL divergence between iterate average and optimal variational approximation. The distinct lines represent repeated experiments and the vertical lines indicate the termination rule trigger points. **(bottom)** Iterations versus symmetrized KL divergence between iterate average and optimal variational approximation at the termination rule trigger point.

**Figure C.22:**
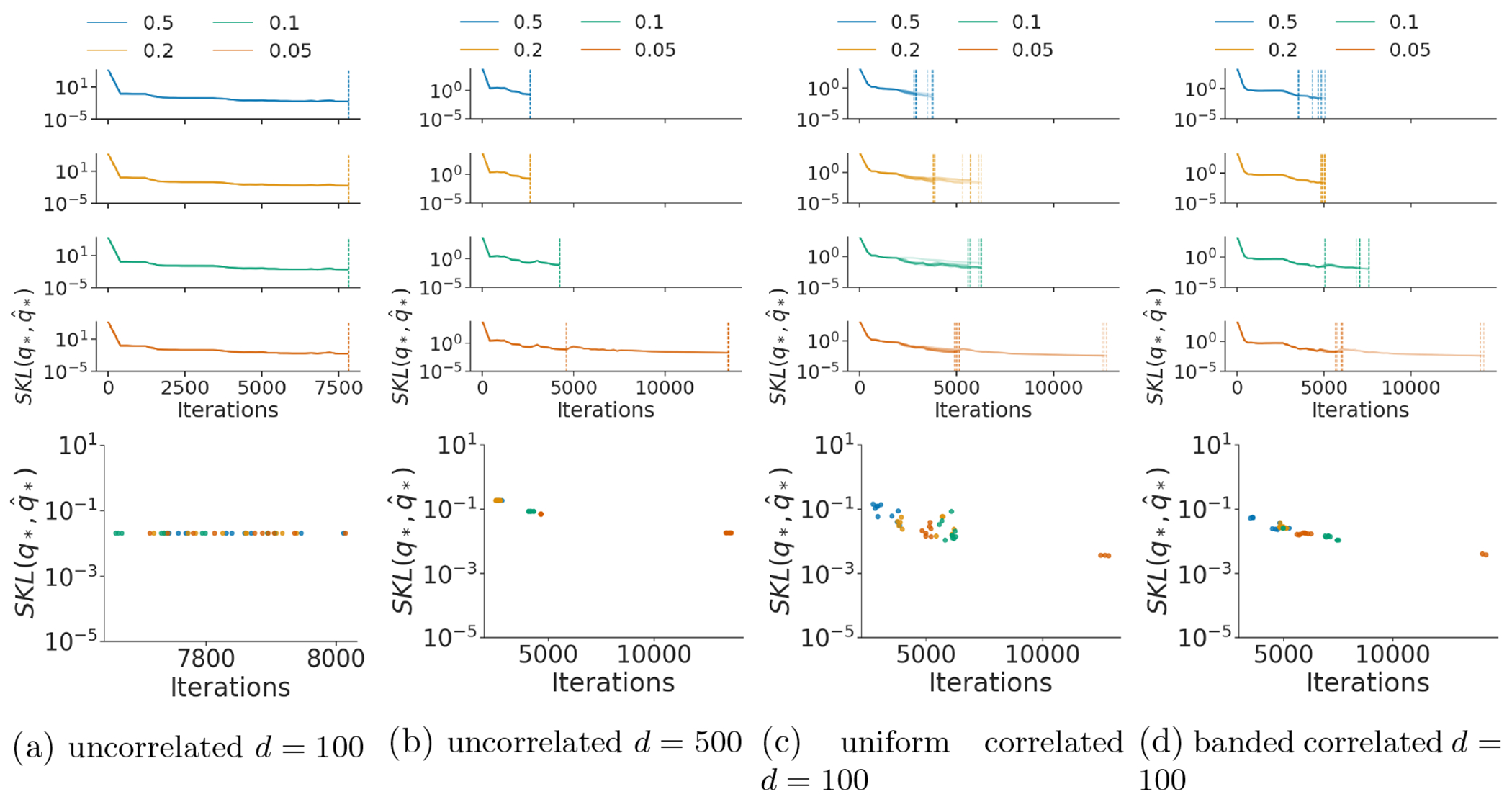
Robustness to initial iterate average relative error threshold *ε* using Gaussian targets. **(top)** Iterations versus symmetrized KL divergence between iterate average and optimal variational approximation. The distinct lines represent repeated experiments and the vertical lines indicate the termination rule trigger points. **(bottom)** Iterations versus symmetrized KL divergence between iterate average and optimal variational approximation at the termination rule trigger point.

**Figure C.23:**
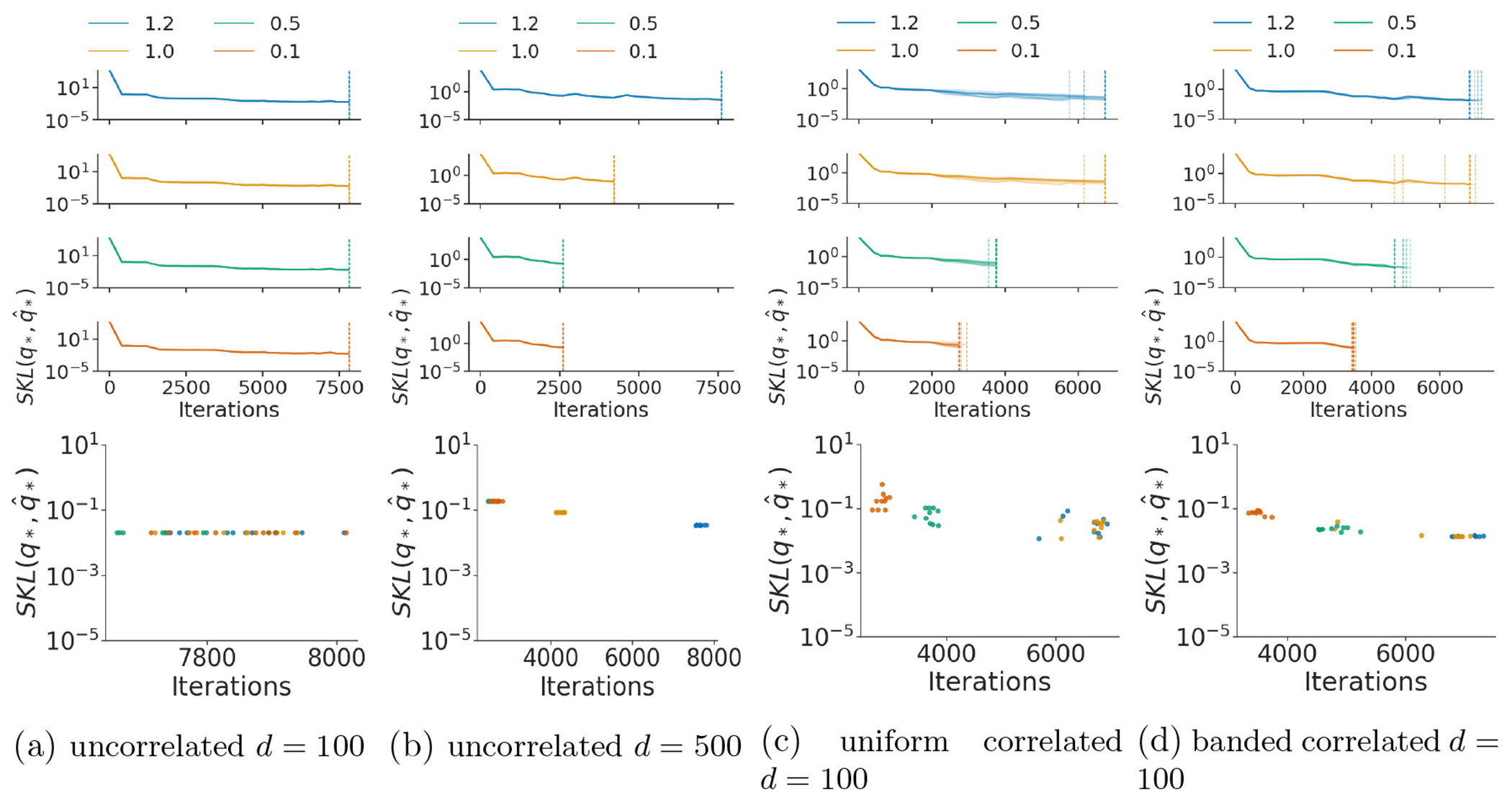
Robustness to inefficiency threshold *τ* using Gaussian targets. **(top)** Iterations versus symmetrized KL divergence between iterate average and optimal variational approximation. The distinct lines represent repeated experiments and the vertical lines indicate the termination rule trigger points. **(bottom)** Iterations versus symmetrized KL divergence between iterate average and optimal variational approximation at the termination rule trigger point.

**Figure C.24:**
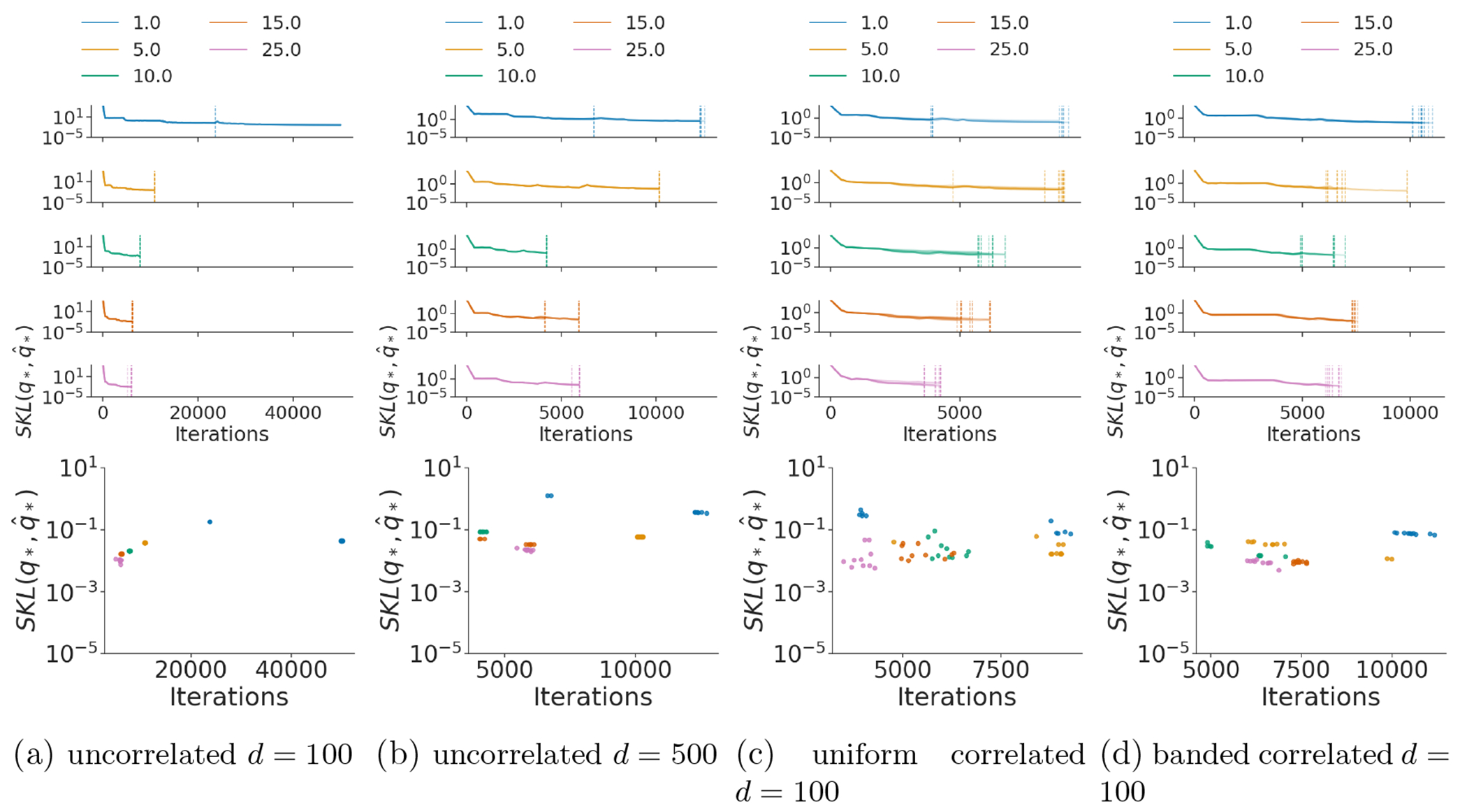
Robustness to Monte Carlo samples *M* using Gaussian targets. **(top)** Iterations versus symmetrized KL divergence between iterate average and optimal variational approximation. The distinct lines represent repeated experiments and the vertical lines indicate the termination rule trigger points. **(bottom)** Iterations versus symmetrized KL divergence between iterate average and optimal variational approximation at the termination rule trigger point.

**Figure C.25:**
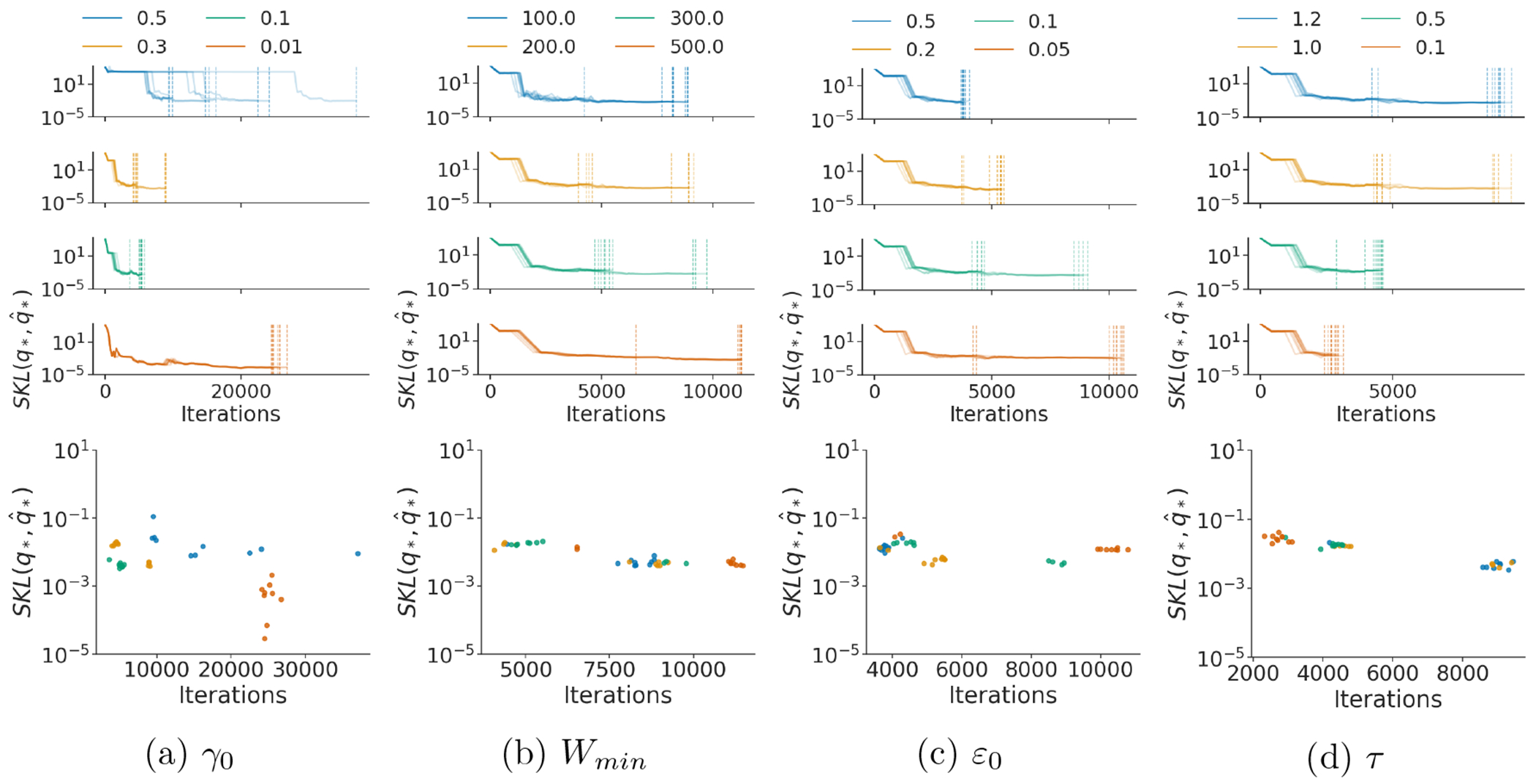
Robustness to tuning parameters (a) initial learning rate $\gamma_{0}$, (b) minimum window size $W_{\text{min}}$, (c) initial iterate average relative error threshold $\varepsilon_{0}$, and (d) inefficiency threshold τ using *arK* dataset from posteriordb package. **(top)** Iterations versus symmetrized KL divergence between iterate average and optimal variational approximation. The transparent lines represent repeated experiments and the vertical lines indicate the termination rule trigger points. **(bottom)** Iterations versus symmetrized KL divergence between iterate average and optimal variational approximation at the termination rule trigger point.

## Footnotes

1.
https://github.com/jhuggins/viabel

2. As stated in Dieuleveut et al. (2020), the $B\gamma^{2}$ term is written as $O\left(\gamma^{2}\right)$. However, the fact that this term is of the form $B\gamma^{2}+o\left(\gamma^{2}\right)$ can be extracted from the proof.

3.
https://github.com/stan-dev/posteriordb

## Appendix A. Proofs

### A.1 Proof of Proposition 6

Since the KL divergence of mean-field Gaussians factors across dimensions, without loss of generality we consider the case of dim=1. The symmetrized KL divergence between two Gaussians is

$$\text{SKL}\left(\lambda_{1},\lambda_{2}\right)=\frac{1}{2}\left\{e^{2\psi_{1}-2\psi_{2}}+e^{2\psi_{2}-2\psi_{1}}+{\left(\tau_{1}-\tau_{2}\right)}^{2}\left(e^{-2\psi_{1}}+e^{-2\psi_{2}}\right)-2\right\}\;\;=\frac{1}{2}\left\{e^{2\psi_{1}-2\psi_{2}}+e^{2\psi_{2}-2\psi_{1}}+{\left(\tau_{1}-\tau_{2}\right)}^{2}e^{-2\psi_{1}}\left(1+e^{2\psi_{1}-2\psi_{2}}\right)-2\right\}.$$

Recall our assumption that for some constants $A=\left(A_{\tau },A_{\psi }\right)$ and $B=\left(B_{\tau },B_{\psi }\right)$,

$${\bar{\lambda }}_{\gamma }=\lambda_{\text{*}}+A\text{γ}+B{\text{γ}}^{2}+o\left({\text{γ}}^{2}\right).$$

Hence

$$e^{2{\bar{\psi }}_{\gamma }-2\psi_{\text{*}}}=1+2A_{\psi }\gamma +2B_{\psi }\gamma^{2}+\frac{1}{2}{\left(2A_{\psi }\gamma +2B_{\psi }\gamma^{2}\right)}^{2}+o\left(\gamma^{2}\right)\;\;=1+2A_{\psi }\gamma +2B_{\psi }\gamma^{2}+2A_{\psi }^{2}\gamma^{2}+o\left(\gamma^{2}\right)$$

and similarly $e^{2\psi_{*}-2{\overset{‾}{\psi }}_{\gamma }}=1-2A_{\psi }\gamma -2B_{\psi }\gamma^{2}+2A_{\psi }^{2}\gamma^{2}+o\left(\gamma^{2}\right)$. Therefore $e^{2{\overset{‾}{\psi }}_{\gamma }-2\psi_{*}}+e^{2\psi_{*}-2{\overset{‾}{\psi }}_{\gamma }}-2=4A_{\psi }^{2}\gamma^{2}+o\left(\gamma^{2}\right)$ and ${\left({\overset{‾}{\tau }}_{\gamma }-\tau_{*}\right)}^{2}=A_{\tau }^{2}\gamma^{2}+o\left(\gamma^{2}\right)$. Putting these results together, we have

$$\text{SKL}\left({\bar{\lambda }}_{\gamma },{\text{λ}}_{\text{*}}\right)=\frac{1}{2}\left[4A_{\psi }^{2}\gamma^{2}+o\left(\gamma^{2}\right)+A_{\tau }^{2}\gamma^{2}e^{-\psi_{\text{*}}}\left\{2-2A_{\psi }\gamma -2B_{\psi }\gamma^{2}+2\gamma^{2}A_{\psi }^{2}+o\left(\gamma^{2}\right)\right\}\right]\;\;=\left(2A_{\psi }^{2}+A_{\tau }^{2}e^{-2\psi_{\text{*}}}\right)\gamma^{2}+o\left(\gamma^{2}\right).$$

Similarly, $SKL\left({\overset{‾}{\lambda }}_{\gamma },{\overset{‾}{\lambda }}_{\gamma^{\prime }}\right)={\left(\gamma -\gamma^{\prime }\right)}^{2}\left(2A_{\psi }^{2}+A_{\tau }^{2}e^{-2\psi_{*}}\right)+o\left(\gamma^{2}\right)$ as long as $\gamma^{\prime }=O(\gamma )$. In other words, letting ${\overset{‾}{\lambda }}_{0}:=\lambda_{*}$, we have the relation

$$SKL\left({\overset{‾}{\lambda }}_{\gamma },{\overset{‾}{\lambda }}_{\gamma^{\prime }}\right)=C{\left(\gamma -\gamma^{\prime }\right)}^{2}+o\left({\text{γ}}^{\text{2}}\right)$$

for some unknown constant *C*.

### A.2 Proof of Proposition 7

Define the Fisher information matrix $F_{\lambda }=E_{\theta ∼q_{\lambda }}\left[-\nabla_{\lambda }^{2}log\left\{q_{\lambda }(\theta )\right\}\right]$. Since $\lambda \mapsto q_{\lambda }(\theta )$ is three-times differentiable, locally the KL divergence behaves in a quadratic form (Amari, 2016):

$$\text{SKL}\left(\lambda_{1},\lambda_{2}\right)=\frac{1}{2}\left\{{\left(\lambda_{1}{-\lambda }_{2}\right)}^{⊤}F_{\lambda_{2}}\left(\lambda_{1}{-\lambda }_{2}\right)+{\left(\lambda_{2}{-\lambda }_{1}\right)}^{⊤}F_{\lambda_{1}}\left(\lambda_{1}{-\lambda }_{2}\right)\right\}+o\left({\left\|\lambda_{1}{-\lambda }_{2}\right\|}^{2}\right).$$

Moreover, by taking first-order Taylor expansion, we have

$$F_{\lambda_{i}}=F_{\lambda_{\text{*}}}+O\left({\left\|\lambda_{*}{-\lambda }_{i}\right\|}_{2}\right).$$

If κ=1, recall that for some constant vectors *A* and *B*,

$${\bar{\lambda }}_{\gamma }=\lambda_{*}+A\gamma +B\gamma^{2}+o\left(\gamma^{2}\right).$$

Assume that $\gamma_{1}=\gamma$ and $\gamma_{2}=O(\gamma )$, so ${\left\|\lambda_{*}-{\overset{‾}{\lambda }}_{\gamma_{i}}\right\|}_{2}=O(\gamma )$. For $\kappa^{\prime }>0$, let $\epsilon_{\kappa^{\prime }}:=\gamma_{1}^{\kappa^{\prime }}-\gamma_{2}^{\kappa^{\prime }}$. Then we have

$$\text{SKL}\left({\bar{\lambda }}_{\gamma_{1}},{\bar{\lambda }}_{\gamma_{2}}\right)=\frac{1}{2}[{\left(A\epsilon_{1}+B\epsilon_{2}+o\left(\gamma^{2}\right)\right)}^{⊤}\left(F_{\lambda_{*}}+O(\gamma )\right)\left(A\epsilon_{1}+B\epsilon_{2}+o\left(\gamma^{2}\right)\right)+{\left(-A\epsilon_{1}-B\epsilon_{2}-o\left(\gamma^{2}\right)\right)}^{⊤}\left(F_{\lambda_{*}}+O(\gamma )\right)\left(-A\epsilon_{1}-B\epsilon_{2}-o\left(\gamma^{2}\right)\right)]+o\left(\|A\epsilon_{1}+B\epsilon_{2}+o{\left(\gamma^{2})\right\|}_{2}^{2}\right)\;\;=A^{⊤}F_{\lambda_{*}}A\epsilon_{1}^{2}+o\left(\gamma^{2}\right)\;\;=C{\left(\gamma_{1}-\gamma_{2}\right)}^{2}+o\left(\gamma^{2}\right),$$

where $C=A^{⊤}F_{\lambda_{*}}A$.

If κ∈[1/2,1), recall that for some constant vectors Λ and *A*,

$${\bar{\lambda }}_{\gamma }=\lambda_{*}+\Lambda \gamma^{\kappa }+A\gamma +o\left(\gamma^{2\kappa }\right).$$

Assume that $\gamma_{1}=\gamma$ and $\gamma_{2}=O(\gamma )$, so ${\left\|\lambda_{*}-{\overset{‾}{\lambda }}_{\gamma_{i}}\right\|}_{2}=O\left(\gamma^{\kappa }\right)$. Then we have

$$\text{SKL}\left({\bar{\lambda }}_{\gamma_{1}},{\bar{\lambda }}_{\gamma_{2}}\right)=\frac{1}{2}[{\left(\text{Λ}\epsilon_{\kappa }+A\epsilon_{1}+o\left(\gamma^{2\kappa }\right)\right)}^{⊤}\left(F_{\lambda_{*}}+O\left(\gamma^{\kappa }\right)\right)\left(\text{Λ}\epsilon_{\kappa }+A\epsilon_{1}+o\left(\gamma^{2\kappa }\right)\right)+{\left(-\text{Λ}\epsilon_{\kappa }-A\epsilon_{1}-o\left(\gamma^{2\kappa }\right)\right)}^{⊤}\left(F_{\lambda_{*}}+O\left(\gamma^{\kappa }\right)\right)\left(-\text{Λ}\epsilon_{\kappa }-A\epsilon_{1}-o\left(\gamma^{2\kappa }\right)\right)]+o\left(\|\Lambda \epsilon_{\kappa }+A\epsilon_{1}+o{\left(\gamma^{2\kappa })\right\|}_{2}^{2}\right)\;\;=\Lambda^{⊤}F_{\lambda_{*}}\text{Λ}\epsilon_{\kappa }^{2}+2{\text{Λ}}^{⊤}F_{\lambda_{*}}A\epsilon_{\kappa }\epsilon_{1}+A^{⊤}F_{\lambda_{*}}A\epsilon_{1}^{2}+o\left(\gamma^{2\kappa }\right)\;\;=\Lambda^{⊤}F_{\lambda_{*}}\text{Λ}\epsilon_{\kappa }^{2}+O\left(\gamma^{1+\kappa }\right)+o\left(\gamma^{2\kappa }\right)\;\;=C{\left(\gamma_{1}^{\kappa }-\gamma_{2}^{\kappa }\right)}^{2}+o\left(\gamma^{2\kappa }\right),$$

where $C=\Lambda^{⊤}F_{\lambda_{*}}\Lambda$.

### A.3 Symmetric KL Divergence Termination Rule

We can use Proposition 7 to derive a termination rule. If γ is the current learning rate, the previous learning rate was γ/ρ. Ignoring $o\left(\gamma^{2\kappa }\right)$ terms, we have

$$\delta_{\gamma }:=\text{SKL}\left({\bar{\lambda }}_{\gamma /\rho },{\bar{\lambda }}_{\gamma }\right)=C\gamma^{2\kappa }{\left(1/\rho^{\kappa }-1\right)}^{2}.$$

Therefore, we can estimate *p* and *C* using a regression model of the form

$$\text{log}\;\delta_{\gamma }=\text{log}\;C+2\;\text{log}\;\left(1/\rho^{\kappa }-1\right)+2p\;\text{log}\gamma .$$

Given estimates $\overset{ˆ}{\kappa }$ and $\overset{ˆ}{C}$, we can estimate $SKL\left({\overset{‾}{\lambda }}_{\gamma },\lambda_{*}\right)\approx \overset{ˆ}{C}\gamma^{2\overset{ˆ}{\kappa }}$.

### A.4 Proof of Proposition 8

Since for |x|<1/2 it holds that |exp(x)−1|≤1.5|x|, we have

$$\frac{\left|{\hat{\sigma }}_{i}-{\bar{\sigma }}_{i}\right|}{{\bar{\sigma }}_{i}}=\left|\text{exp}\left({\hat{\psi }}_{i}-{\bar{\psi }}_{i}\right)-1\right|\leq 1.5\varepsilon$$

and

$$\frac{\left|{\hat{\tau }}_{i}-{\bar{\tau }}_{i}\right|}{{\bar{\sigma }}_{i}}\leq \varepsilon^{\prime }{\hat{\sigma }}_{i}/{\bar{\sigma }}_{i}\leq \varepsilon^{\prime }(1+1.5\varepsilon )\leq 1.75\varepsilon .$$

### A.5 Proof of Proposition 9

In the optimal case, the total cost for the iterations used for iterate averaging is

$$\text{OPT}=C_{O}W_{\text{opt}}+C_{E}W_{\text{conv}}+C_{E}W_{\text{opt}}\;\;=C_{E}\left(rW_{\text{opt}}+W_{\text{conv}}+W_{\text{opt}}\right).$$

On the other hand, if checking at window sizes $W_{j}:=\chi^{j}W_{\text{conv}}(j=1,2,\ldots )$, then the window size at which convergence will be detected is $j_{*}:=\left\lceil log\left(W_{\text{opt}}/W_{\text{conv}}\right)/log(\chi )\right\rceil$. In particular,

$$W_{j_{*}}\leq \chi W_{\text{opt}}.$$

Therefore we can bound the actual computational cost as

$$C_{O}W_{j_{*}}+C_{E}W_{\text{conv}}\text{log}W_{\text{conv}}+C_{E}\overset{j_{*}}{\underset{j=1}{\sum }}W_{j}\;\;\leq C_{O}\chi W_{\text{opt}}+C_{E}W_{\text{conv}}\text{log}W_{\text{conv}}+C_{E}\overset{j_{*}}{\underset{j=1}{\sum }}\chi^{j}W_{\text{conv}}\;\;\leq C_{O}\chi W_{\text{opt}}+C_{E}W_{\text{conv}}\text{log}W_{\text{conv}}+C_{E}\frac{\chi \left(\chi^{j_{*}}-1\right)W_{\text{conv}}}{\chi -1}\;\;\leq C_{O}\chi W_{\text{opt}}+C_{E}W_{\text{conv}}\text{log}W_{\text{conv}}+C_{E}\frac{\chi^{2}W_{\text{opt}}}{\chi -1}\;\;=C_{E}\left[\chi rW_{\text{opt}}+W_{\text{conv}}+\frac{\chi^{2}}{\chi -1}W_{\text{opt}}\right].$$

If χ=2, then the actual computational cost is bounded by

$$C_{E}\left[2rW_{\text{opt}}+W_{\text{conv}}+4W_{\text{opt}}\right]\leq 4C_{E}\left[rW_{\text{opt}}+W_{\text{conv}}+W_{\text{opt}}\right]\;\;=4\text{OPT}.$$

On the other hand, we can minimize the total cost by solving $\chi_{*}:=arg\;{min}_{\chi >1}\{r\chi +\chi^{2}/(\chi -1)\}=\chi (r)$. Plugging this back in, we get the bound

$$C_{E}\frac{2+r+2\sqrt{1+r}}{1+r}\left(rW_{\text{opt}}+W_{\text{conv}}\text{log}W_{\text{conv}}+W_{\text{opt}}\right)\;\;=\frac{2+r+2\sqrt{1+r}}{1+r}\text{OPT}\;\;<4\text{OPT}.$$

## Appendix B. Further Details

### B.1 Effective Sample Size (ESS) and Monte Carlo Standard Error (MCSE)

Let $v^{\left(1\right)},v^{\left(2\right)},\ldots$ denote a stationary Markov chain, let $\overset{‾}{v}:=E[v^{\left(1\right)}]$ denote the mean at stationarity, and let $\overset{ˆ}{v}:=K^{-1}\sum_{k=1}^{K}v^{\left(k\right)}$ denote the Monte Carlo estimate for $\overset{‾}{v}$. The (ideal) effective sample size is defined as

$$\text{ESS}(K):=K/\left(1+\overset{\infty }{\underset{k=1}{\sum }}\rho_{k}\right),$$

where $\rho_{k}$ is the autocorrelation of the Markov chain at lag *k*. The ESS can be efficiently estimated using a variety of methods (Geyer, 1992; Vehtari et al., 2021). We write $\hat{ESS}\left(v^{\left(1:K\right)}\right)$ to denote an estimator for ESS(K) based on the sequence $v^{\left(1:K\right)}:=\left(v^{\left(1\right)},\ldots ,v^{\left(K\right)}\right)$. The Monte Carlo standard error of $\overset{ˆ}{v}$ is given by

$$\mathrm{MCSE}\left(\hat{v}\right):=\sigma \left(v^{\left(1\right)}\right)/\text{ESS}(K),$$

where $\sigma \left(v^{\left(1\right)}\right)$ denote the standard deviation of the random variable $v^{\left(1\right)}$. Given the empirical standard deviation of $v^{\left(1\right)},\ldots ,v^{\left(K\right)}$, which we denote $\overset{ˆ}{\sigma }\left(v^{\left(1:K\right)}\right)$, the MCSE can be approximated by

$$\hat{\text{MCSE}}\left(v^{\left(1:K\right)}\right):=\hat{\sigma }\left(v^{\left(1:K\right)}\right)/\hat{\text{ESS}}\left(v^{\left(1:K\right)}\right).$$

### B.2 Total Variation Distance and KL Divergence

For distributions η and ζ, $\left|I_{\eta ,i,a,b}-I_{\zeta ,i,a,b}\right|\leq \sqrt{KL(\eta |\zeta )/2}$ for all a<b and *i*. This guarantee follows from Pinsker’s inequality, which relates the KL divergence to the total variation distance

$$d_{\text{TV}}(\eta ,\zeta ):=\underset{f:\|f{\|}_{\infty }\leq 1}{\text{sup}}\left|\int f(\theta )\eta (\text{d}\theta )-\int f(\theta )\zeta (\text{d}\theta )\right|,$$

where $\left\|f{\|}_{\infty }:={sup}_{\theta }f(\theta )-{inf}_{\theta }f(\theta \right)$. Specifically, Pinsker’s inequality states that $d_{TV}(\eta ,\zeta )\leq \sqrt{KL(\eta |\zeta )/2}$. Thus, small KL divergence implies small total variance distance, which implies the difference between expectations for any function *f* such that $\|f{\|}_{\infty }$ is small. In the case of interval probabilities, since $\|𝟙(\cdot \in [a,b]){\|}_{\infty }=1$, it follows that

$$\left|I_{\eta ,i,a,b}-I_{\zeta ,i,a,b}\right|=\left|\int 𝟙(\theta \in [a,b])\eta (\text{d}\theta )-\int 𝟙(\theta \in [a,b])\zeta (\text{d}\theta )\right|\;\;\leq \|𝟙(\cdot \in [a,b]){\|}_{\infty }d_{\text{TV}}(\eta ,\zeta )\;\;\leq \sqrt{\text{KL}(\eta |\zeta )/2}.$$

### B.3 Adaptive SASA+

The SASA+ algorithm of Zhang et al. (2020) generalizes the approach of Yaida (2019). The main idea is to find an appropriate *invariant function* Δ(d,λ) that satisfies $\int \Delta (d,\lambda )\mu_{\gamma }(dd,d\lambda )=0$. Yaida (2019) derived valid forms of Δ for specific choices of the descent direction, while Zhang et al. (2020) showed that for any optimizer of the form Eq. (1) that is time-homogenous with $\gamma^{\left(k\right)}=\gamma$, the map $(d,\lambda )\mapsto 2\langle d,\lambda \rangle -\gamma \|d{\|}^{2}$ is a valid invariant function. The SASA+ algorithm proposed by Zhang et al. (2020) uses a hypothesis test to determine when the iterates are sufficiently close to stationarity. Let $\Delta^{\left(k\right)}:=2\langle d^{\left(k\right)},\lambda^{\left(k\right)}\rangle -\gamma {\left\|d^{\left(k\right)}\right\|}^{2}$ and let W=⌈ϱk⌉ denote the window size to use for checking stationarity. Once *W* is at least equal to a minimum window size $W_{min}$, SASA+ uses $\Delta^{\left(k-W+1\right)},\ldots ,\Delta^{\left(k\right)}$ to carry out a hypothesis test, where the null hypothesis is that $E[\Delta^{\left(k\right)}]=0$.

We make several adjustments to reduce the number of tuning parameters and to make the remaining ones more intuitive. Note that the SASA+ convergence criterion requires the choice of three parameters: ϱ, $W_{min}$, and the size of the hypothesis test α. Zhang et al. (2020) showed empirically and our numerical experiments confirmed that the choice of α has little effect and therefore does not need to be adjusted by the user. The choices for ϱ and $W_{min}$, however, have a substantial effect on efficiency. If ϱ is too big, then early iterations that are not at stationarity will be included, preventing the detection of convergence. On the other hand, if ϱ is too small, then the total number of iterations must be large (specifically, greater than $W_{min}/\varrho$) before the window size is large enough to trigger the first check for stationarity. Moreover, the correct choice of $W_{min}$ will vary depending on the problem. If the iterates have large autocorrelation then $W_{min}$ should be large, while if the autocorrelation is small or negative, then $W_{min}$ can be small.

Our approach to determining the optimal window size instead relies on the effective sample size (ESS). $W_{\text{opt}}$ that maximizes $\hat{ESS}(W):=\hat{ESS}(\Delta^{\left(k-W+1\right)},\ldots ,\Delta^{\left(k\right)})$, where *k* is the current iteration. To ensure reliability, we impose additional conditions on $W_{\text{opt}}$. First, we require that $\hat{ESS}(W_{\text{opt}})\geq N_{min}$, a user-specified minimum effective sample size. Unlike $W_{\text{min}}$, $N_{\text{min}}$ has an intuitive and direct interpretation. The second condition is, when finding $W_{\text{opt}}$, the search over values of *W* is constrained to the lower bound of $N_{min}$ (to ensure the estimator is sufficiently reliable) and the upper bound of 0.95*k* (to always allow for some “burn-in”). In practice we do not check all $W\in \left\{N_{min},\ldots ,0.95k\right\}$, but rather perform a grid search over 5 equally spaced values ranging from $N_{\text{min}}$ to 0.95*k*.

A slightly different version which may improve power is to instead define the multivariate invariant function $\vec{\Delta }(d,\lambda )=2d⊙\lambda -d⊙d$, where we recall that ⊙ denotes component-wise multiplication. In this case a multivariate hypothesis test such as Hotelling’s $T^{2}$ test or the multivariate sign test, where a single effective sample size (for example, the median component-wise ESS) could be used. Alternatively, a separate hypothesis test for each of the *m* components could be used, with stationarity declared once all tests confirm stationarity (for example using a test size of α/m). While this approach might be more computationally efficient when *m* is large, it could come at the cost of test power.

### B.4 Distance Based Convergence Detection

Pesme et al. (2020) proposed a distance-based diagnostic algorithm to detect the stationarity of the SGD optimization algorithm. The main idea behind this algorithm is to find the distance between the current iterate $\lambda_{k}$ and the optimal variational parameter $\lambda_{*}$, $\left\|\eta_{k}\right\|:=\left\|\lambda_{k}-\lambda_{*}\right\|$. Since the optimal variational parameter, $\lambda_{*}$ is unknown, this distance cannot be directly observed. Therefore, they suggested using the distance between the current iterate $\lambda_{k}$ and the initial iterate of the current learning rate, $\left\|\Omega_{k}\right\|:=\left\|\lambda_{k}-\lambda_{0}\right\|$ and showed that $\left\|\eta_{k}\right\|$ and $\left\|\Omega_{k}\right\|$ have a similar behavior.

Under the setting of the quadratic objective function with additive noise, they computed the behavior of the expectation of ${\left\|\Omega_{k}\right\|}^{2}$ in closed-form to detect the convergence of ${\left\|\lambda_{k}-\lambda_{0}\right\|}^{2}$. With the result, they have shown the asymptotic behavior of the $E[{\left\|\Omega_{k}\right\|}^{2}]$ in the transient and stationary phases, where $E[{\left\|\Omega_{k}\right\|}^{2}]$ has a slope greater than 1 and slope of 0 in a log-log plot respectively. Hence, the slope $S:=\frac{log{\left\|\lambda_{k}-\lambda_{0}\right\|}^{2}-log{\left\|\lambda_{k}/q-\lambda_{0}\right\|}^{2}}{logk-logk/q}$ computed between iterations $q^{n}$ and $q^{n+1}$ for q>1 and $n\geq n_{0}$ where $n_{0}\in {𝒩}^{*}$ and if *S* < thresh (thresh∈(0,2]) then the convergence will be detected.

### B.5 Stochastic Quasi-Newton Optimization

The algorithm of Liu and Owen (2021) provides a randomized approach of the classical quasi-Newton (QN) method that is known as the stochastic quasi-Newton (SQN) method. Even though, Liu and Owen (2021) uses randomized quasi-Monte Carlo (RMCQ) samples we use Monte Carlo (MC) samples to compute the gradient. In classical quasi-Newton optimization, the Newton update is

$$\lambda^{\left(k+1\right)}\leftarrow \lambda^{\left(k\right)}-{\left(\nabla_{\lambda^{\left(k\right)}}^{2}D_{\pi }\left(q_{\lambda^{\left(k\right)}}\right)\right)}^{-1}\nabla_{\lambda^{\left(k\right)}}D_{\pi }\left(q_{\lambda^{\left(k\right)}}\right),$$

where $\nabla_{\lambda^{\left(k\right)}}^{2}D_{\pi }\left(q_{\lambda^{\left(k\right)}}\right)$ is the Hessian matrix of $D_{\pi }\left(q_{\lambda^{\left(k\right)}}\right)$. However, the computation cost of the Hessian matrix and its inverse is high, and it also requires a large amount of space. Therefore, we can use BFGS (discovered by Broyden, Fletcher, Goldfarb, and Shanno) method where it approximate the inverse of $\nabla_{\lambda^{\left(k\right)}}^{2}D_{\pi }\left(q_{\lambda^{\left(k\right)}}\right)$ using $H_{k}$ at the *k*th iteration by initializing it with an identity matrix. Then the update is modified by

$$\lambda^{\left(k+1\right)}\leftarrow \lambda^{\left(k\right)}-\gamma^{\left(k\right)}H^{\left(k\right)}\nabla_{\lambda^{\left(k\right)}}D_{\pi }\left(q_{\lambda^{\left(k\right)}}\right),$$

where

$$H^{\left(k+1\right)}\leftarrow \left(I-\frac{s_{k}y_{k}^{⊤}}{s_{k}^{⊤}y_{k}}\right)H^{\left(k\right)}\left(I-\frac{y_{k}s_{k}^{⊤}}{s_{k}^{⊤}y_{k}}\right)+\frac{s_{k}s_{k}^{⊤}}{s_{k}^{⊤}y_{k}},$$

$s_{k}=\lambda^{\left(k+1\right)}-\lambda^{\left(k\right)}$, and $y_{k}=\nabla_{\lambda^{\left(k+1\right)}}D_{\pi }\left(q_{\lambda^{\left(k+1\right)}}\right)-\nabla_{\lambda^{\left(k\right)}}D_{\pi }\left(q_{\lambda^{\left(k\right)}}\right)$. Even using the above Hessian approximation $H^{\left(k\right)}$ will require a large space for storage. To overcome that problem we can use Limited-memory BFGS (L-BFGS) (Nocedal and Wright, 2006) that computes $H^{\left(k\right)}\nabla_{\lambda^{\left(k\right)}}D_{\pi }\left(q_{\lambda^{\left(k\right)}}\right)$ using *m* most recent $\left(s_{k},y_{k}\right)$ correction pairs. Liu and Owen (2021) use the stochastic quasi-Newton approach proposed by Chen et al. (2019) and they compute the correction pairs after every *B* iterations by computing the iterate average of parameters using the most recent *B* iterations. To compute the $y_{k}$, the gradients of the objective function are estimated using Monte Carlo samples that are independent of the samples used to compute the gradient in the update step.

### B.6 Natural Gradient Descent Optimization

Khan and Lin (2017) proposed a natural gradient descent (NGD) approach for the variational inference that uses the information geometry of the variational distribution. Given the variational distribution is an exponential family distribution

$$q_{\lambda }=h\;\text{exp}\left(\phi^{⊤}\lambda -A(\lambda )\right)$$

the NGD update is

$$\lambda^{\left(k+1\right)}\leftarrow \lambda^{\left(k\right)}-\gamma^{\left(k\right)}{\left(F\left(\lambda^{\left(k\right)}\right)\right)}^{-1}\nabla_{\lambda^{\left(k\right)}}D_{\pi }\left(q_{\lambda^{\left(k\right)}}\right),$$

where $F(\lambda^{\left(k\right)})$ denotes the Fisher information matrix (FIM) of the distribution and λ denotes its natural parameter. Without directly computing the inverse of FIM Khan and Lin (2017) simplified the above update by using the relationship between natural parameter λ and expectation parameter $m=E_{q}[\phi ]$ of the exponential family

$$F{\left(\lambda \right)}^{-1}\nabla_{\lambda }D_{\pi }\left(q_{\lambda }\right)=\nabla_{m}D_{\pi }\left(q_{m}\right).$$

Therefore, the natural-gradient update can be simplified as

$$\lambda^{\left(k+1\right)}\leftarrow \lambda^{\left(k\right)}-\gamma^{\left(k\right)}\nabla_{m^{\left(k\right)}}D_{\pi }\left(q_{m^{\left(k\right)}}\right).$$

This can be applied for the mean-field Gaussian variational family $q=\prod_{i=1}^{d}𝒩\left(\mu_{i},\sigma_{i}^{2}\right)$ where we can define the natural parameters $\lambda_{i}=\left(\mu_{i}/\sigma_{i}^{2},-0.5/\sigma_{i}^{2}\right)$ and expectation parameters $m=\left(\mu_{i},\mu_{i}^{2}+\sigma_{i}^{2}\right)$.

## Appendix C. Additional Experimental Details and Results

**Table C.1:** Datasets of PosteriorDB Package

Dataset	Short Name	D	Model Description
arK-arK	arK	7	AR(5) time series
bball_drive_event_0-hmm_drive_0	bball_0	8	Hidden Markov model
bball_drive_event_1-hmm_drive_1	bball_1	8	Hidden Markov model
diamonds-diamonds	diamonds	26	Log-Log
dogs-dogs	dogs	3	Logistic mixed-effects
dogs-dogs_log	dogs_log	2	Logarithmic mixed-effects
earnings-logearn_interaction	earnings	5	Log-linear
eight_schools-eight_schools_noncentered	8schools_nc	10	Non-centered hierarchical
eight_schools-eight_schools_centered	8schools_c	10	Centered hierarchical
garch-garch11	garch	4	GARCH(1,1) time series
gp_pois_regr-gp_pois_regr	gp_pois_regr	13	Gaussian process Poisson regression
gp_pois_regr-gp_regr	gp_regr	3	Gaussian process regression
hmm_example-hmm_example	hmm_example	6	Hidden Markov model
hudson_lynx-hare_lotka_volterra	hudson_lynx	8	Lotka-Volterra error
low_dim_gauss_mix-low_dim_gauss_mix	low_dim_gauss_mix	5	Two-dimensional Gaussian mixture
mcycle_gp-accel_gp	mcycle_gp	66	Gaussian process
nes2000-nes	nes2000	10	Multiple predictor linear
sblrc-blr	sblrc	6	Linear
